# Pyrrole-based inhibitors of RND-type efflux pumps reverse antibiotic resistance and display anti-virulence potential

**DOI:** 10.1371/journal.ppat.1012121

**Published:** 2024-04-09

**Authors:** Nisha Mahey, Rushikesh Tambat, Ritu Kalia, Rajnita Ingavale, Akriti Kodesia, Nishtha Chandal, Srajan Kapoor, Dipesh Kumar Verma, Krishan Gopal Thakur, Sanjay Jachak, Hemraj Nandanwar

**Affiliations:** 1 Clinical Microbiology & Antimicrobial Research Laboratory, CSIR-Institute of Microbial Technology, Sector 39-A, Chandigarh, India; 2 Academy of Scientific & Innovative Research (AcSIR), Ghaziabad, Uttar Pradesh, India; 3 Department of Natural Products, National Institute of Pharmaceutical Education and Research, Mohali, India; 4 Structural Biology Laboratory, CSIR-Institute of Microbial Technology, Chandigarh, India; Universite Paris Descartes Faculte de Medecine, FRANCE

## Abstract

Efflux pumps of the resistance-nodulation-cell division (RND) superfamily, particularly the AcrAB-TolC, and MexAB-OprM, besides mediating intrinsic and acquired resistance, also intervene in bacterial pathogenicity. Inhibitors of such pumps could restore the activities of antibiotics and curb bacterial virulence. Here, we identify pyrrole-based compounds that boost antibiotic activity in *Escherichia coli* and *Pseudomonas aeruginosa* by inhibiting their archetype RND transporters. Molecular docking and biophysical studies revealed that the EPIs bind to AcrB. The identified efflux pump inhibitors (EPIs) inhibit the efflux of fluorescent probes, attenuate persister formation, extend post-antibiotic effect, and diminish resistant mutant development. The bacterial membranes remained intact upon exposure to the EPIs. EPIs also possess an anti-pathogenic potential and attenuate *P*. *aeruginosa* virulence *in vivo*. The intracellular invasion of *E*. *coli* and *P*. *aeruginosa* inside the macrophages was hampered upon treatment with the lead EPI. The excellent efficacy of the EPI-antibiotic combination was evidenced in animal lung infection and sepsis protection models. These findings indicate that EPIs discovered herein with negligible toxicity are potential antibiotic adjuvants to address life-threatening Gram-negative bacterial infections.

## Author summary

Bacteria evolved numerous mechanisms to stop the action of antibiotics. Efflux pumps are such vital molecular machinery that constantly exports antibiotics out of the bacterial cell. As a result, the antibiotics fail to accumulate sufficiently, thereby decreasing the bacteria’s susceptibility to antibiotics. One excellent strategy to tackle Multi-drug resistant (MDR) pathogens is to inhibit these efflux pumps and thereby increase the pathogen sensitivity to existing/obsolete antibiotics. This study identified pyrrole derivatives as efflux pump inhibitors and highlights their potential to increase the susceptibility of MDR *E*. *coli*, *P*. *aeruginosa*, and *K*. *pneumoniae* to multiple antibiotics *in vitro*. Moreover, we observed that the combination displayed remarkable efficacy in reducing bacterial burden *in vivo*. Additionally, this study provides a glance at the accessory anti-virulence properties of the EPIs as a secondary effect of efflux pump inhibition. Altogether, the present study provides relevant clues to consider combination therapy as a go-to solution for MDR bacterial infections.

## Introduction

The current antimicrobial resistance (AMR) crisis is worsened by multi-drug resistant (MDR) Gram-negative bacterial infections. Since it is challenging to find antimicrobial agents targeting Gram-negative bacteria, infections pertaining to *Escherichia coli*, *Pseudomonas aeruginosa*, and *Klebsiella pneumoniae* are the worst nightmares. These superbugs have acquired resistance to most antibiotics (all antibiotics in some cases) available clinically and are categorized as critical priority pathogens by the World Health Organization [1]. A recent study estimates that each of these pathogens was responsible for more than 250000 AMR-associated deaths alone in 2019 [2].

Over-expression of efflux pumps, particularly those belonging to the resistance nodulation cell division (RND) family, are key determinants of multi-drug resistance in Gram-negative bacteria. These pumps can recognize and expel a range of chemically diverse compounds, including antibiotics, biocides, and detergents, to the exterior of the cell [3] by a coupled proton or ion exchange. Moreover, the polyspecificity of the RND transporters is known to contribute to the acquisition of additional resistance mechanisms along with the efflux pump-based resistance phenotype [4]. These pumps possess a tripartite structure, e.g., AcrAB-TolC of *E*. *coli* comprises of AcrB (the integral membrane transporter), AcrA (the periplasmic adapter), and the outer membrane channel TolC [3]. The main archetypes of RND family transporters in clinical isolates are the AcrAB-TolC in *Enterobacteriaceae* and its homologue MexAB-OprM in *P*. *aeruginosa* [4]. Moreover, the *mexAB* genes of *P*. *aeruginosa* are highly similar to *acrAB* of *E*. *coli* [5] (69.8% amino acid identity and 83.2% similarity) [6]. It is noteworthy that the RND pumps of bacteria and humans share low homology (16% identity), which makes them an "excellent" therapeutic target [7].

The combination therapy with existing/obsolete antibiotics has gained considerable attention due to its sustainable approach to antibiotic revival. Multiple RND efflux pump inhibitors (EPIs) have been identified earlier, including phenylalanine arginine-β-naphthylamide (PAβN) [8] and 1-(1-naphthylmethyl)-piperazine (NMP) [9]. These two possess the most potent efflux inhibitory ability but could not be used clinically due to their nephrotoxicity and serotonin agonist properties [9,10]. Since RND efflux pumps, particularly in *P*. *aeruginosa*, are known to extrude some bacterial molecules necessary for virulence [11,12], and other quorum-sensing signals [13], EPIs could hamper pathogen virulence as an adjunctive anti-virulence agents [14,15]. Also, a well-known EPI PAβN is previously reported to exhibit anti-virulence activity against *P*. *aeruginosa* [12]. Nonetheless, *P*. *aeruginosa* is a critical priority pathogen responsible for creating havoc in burn units of hospitals, patients with cystic fibrosis, implanted medical devices (stents, catheters, and intubation tubes) by its extensive repertoire of virulence factors, biofilm formation potential [16]. The strategy could be relevant because EPIs and anti-virulence agents may not impose intense selective pressure on bacteria and contribute to resistance insurgence as they do not affect bacterial viability [15]. Besides, a compound possessing dual properties both of an efflux inhibitor and an anti-virulence agent can be an excellent drug candidate with enhanced therapeutic potential.

This study describes the synthesis, screening, and identification of new pyrrole derivatives as EPIs targeting the RND family of efflux transporters. Also, pyrrole compounds have previously been known to possess RND efflux pump inhibitory potential [17,18]. Initially, we synthesized 24 derivatives by modifications around the pyrrole scaffold. Further, the compounds were screened for efflux pump inhibitory potential against the AcrB over-expressed *E*. *coli* to discover putative EPI lead. Our study demonstrates inhibitors exhibiting efflux pump inhibition activity displaying strong synergism with existing antibiotics. The compounds also display anti-virulence potential by reducing the invasiveness of *E*. *coli* and *P*. *aeruginosa* in macrophages and attenuating *P*. *aeruginosa* virulence in *Caenorhabditis elegans* by inhibiting various virulence factors. Our best EPI exhibits remarkable *in vivo* efficacy by reducing the bacterial load in the murine lung infection model and protecting sepsis-induced mice. The study provides evidence for compounds’ safety *in vitro* and in murine acute toxicity studies. Conclusively, the study identifies a lead molecule that can be considered for expanding the drug armamentarium against drug-resistant Gram-negative pathogens.

## Results

### Pyrrole derivatives potentiate antibiotic activity *in vitro*

**Fig 1:**
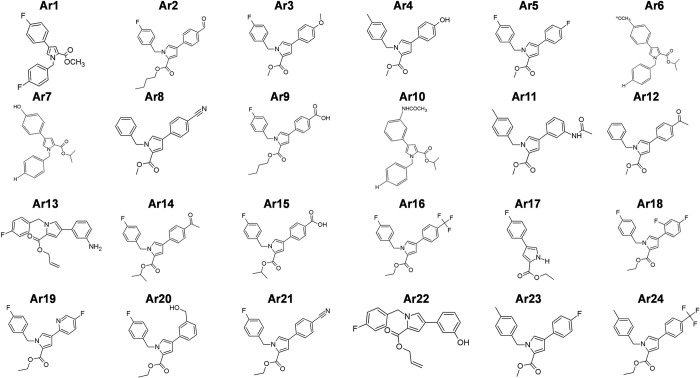
Representative 2D structures of the 24 pyrrole-based derivatives synthesized for screening. The structures were drawn using ACD/ChemSketch 2016 2.2 freeware.

**Fig 2:**
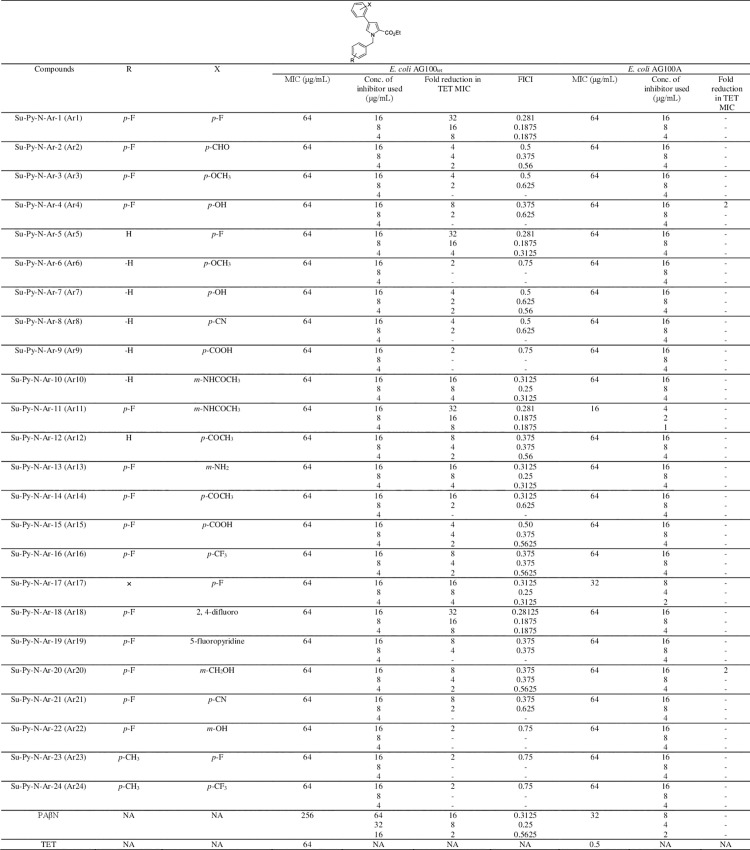
The structure of the parent pyrrole and tetracycline boosting activities of compounds Ar1-Ar24 in *E*. *coli* AcrB over-expressing (AG100_tet_) and AcrAB deletion (AG100A) strains as determined by using the broth microdilution checkerboard synergy method. The assay was performed in three biological replicates having two technical replicates. MIC, Minimum Inhibitory Concentration; TET, Tetracycline; FICI, Fractional Inhibitory Concentration Index; ’-’ represents no fold reduction in tetracycline MIC; PAβN, Phenylalanine-arginine-β-naphthylamide.

Initially, we assessed the synergistic potential of synthesized pyrrole derivatives (Fig 1) with tetracycline (AcrB efflux pump substrate) against the *E*. *coli* AG100_tet_ (over-expressing AcrB) and the *E*. *coli* AG100A (AcrAB pump-deficient) using checkerboard broth microdilution assay. The minimum inhibitory concentrations (MIC) of compounds were found 64 μg/mL. At concentrations (sub-inhibitory) used to potentiate antibiotic efficacy, compounds alone had no detectable growth inhibition activity against all the tested strains. Most of the compounds enhanced the activity of tetracycline by 2–32 folds in a concentration-dependent manner against the *E*. *coli* AG100_tet_ strain (Fig 2). Remarkably, the combination of Ar1, Ar5, Ar11, and Ar18 at 16 μg/mL and 8 μg/mL with tetracycline possessed the prominent synergism [Fractional inhibitory concentration index (FICI) = 0.281, and 0.1875 i.e., FICI ≤0.5], which enabled the minimum inhibitory concentration (MIC) value of tetracycline to decrease from 64 to 2 μg/mL and 4 μg/mL, respectively. Most importantly, these four most active compounds displayed a better potentiation effect than PAβN, a known RND efflux pump inhibitor. In addition, compounds did not mediate tetracycline boosting in *E*. *coli* AG100A (Δ*acrAB*). Considering the structure-activity relationship (SAR), the electron-donating groups like methyl and hydrogen in the *para*-position of the benzyl ring proved to be non-beneficial for the activity (Ar6, Ar7, Ar8, Ar9, Ar12, Ar23, and Ar24) as compared to fluorine-containing derivatives. The introduction of electron-donating substituents at the C-4 position of the phenyl ring attached to the pyrrole scaffold resulted in reduced activity (Ar2, Ar3, and Ar6). The SAR revealed that the presence of fluorine-substituted benzyl ring and electron-withdrawing groups containing aryl ring attached at the C-4 position of the pyrrole scaffold are the key features for bioactivity (S1 Fig).

We further tested the potentiation of Ar1, Ar5, Ar11, and Ar18 to RND efflux pump substrates (ciprofloxacin, levofloxacin, tetracycline, tigecycline, erythromycin, piperacillin, and chloramphenicol) against XDR *E*. *coli* ATCC BAA-2774 (expressing AcrAB-TolC), *K*. *pneumoniae* ATCC BAA-2782 (expressing AcrAB-TolC), and *P*. *aeruginosa* ATCC BAA-2795 (expressing MexAB-OprM). All four compounds at 16 μg/mL (1/4 × MIC) displayed ≥ 4-fold antibiotic boosting activity (S1 and S2 Tables). The potentiation effect observed was comparable to or even better than the existing potentiators, i.e., PAβN and NMP. The compounds did not exhibit synergy with any of the antibiotics tested against *P*. *aeruginosa* PAO750 (PAO1: *ΔmexAB-oprM*, *ΔmexCD-oprJ*, *ΔmexEF-oprN*, *ΔmexXY*) (S3 Table). In the validation experiment, Ar1, Ar5, Ar11, and Ar18 did not alter the activity of tobramycin, gentamicin, and amikacin (non-MexB substrates) (S4 Table). The fact that compounds potentiate a given antibiotic against efflux pump over-expressed strain but not deletion provides substantial evidence that the inhibition effect is RND efflux pump specific targeting AcrAB and MexAB efflux systems.

### The compounds modulate bacterial efflux pump activity

To further confirm that the synergy observed is due to AcrB efflux pump inhibition, we performed Hoechst 33342 accumulation and efflux inhibition assays against *E*. *coli* AG100_tet_. Hoechst 33342 is a membrane-permeate DNA-binding dye, a known substrate of RND efflux pumps, therefore employed as a reporter for quantitation of transport across bacterial cells [18]. The relative final fluorescence (RFF) values were calculated for all the compounds. RFF value is a difference between the final fluorescence of treated cells compared to untreated cells and is a direct measure of the efflux inhibitory potential of the compound [19]. RFF value ˃1 denotes the increased accumulation of the dye inside bacterial cells. For most of the compounds, RFF values were observed to be ˃1, while the most efficient compounds were Ar1 (RFF = 7.42), Ar5 (RFF = 7.68), Ar11 (RFF = 7.65), and Ar18 (RFF = 7.51) as compared to PAβN with RFF = 4.71. We observed a dose-dependent enhancement in dye accumulation in the bacterial cells when incubated in the presence of compounds (1/4 × MIC, 1/8 × MIC, 1/16 × MIC) which was higher as compared to PAβN (S5 Table).

**Fig 3:**
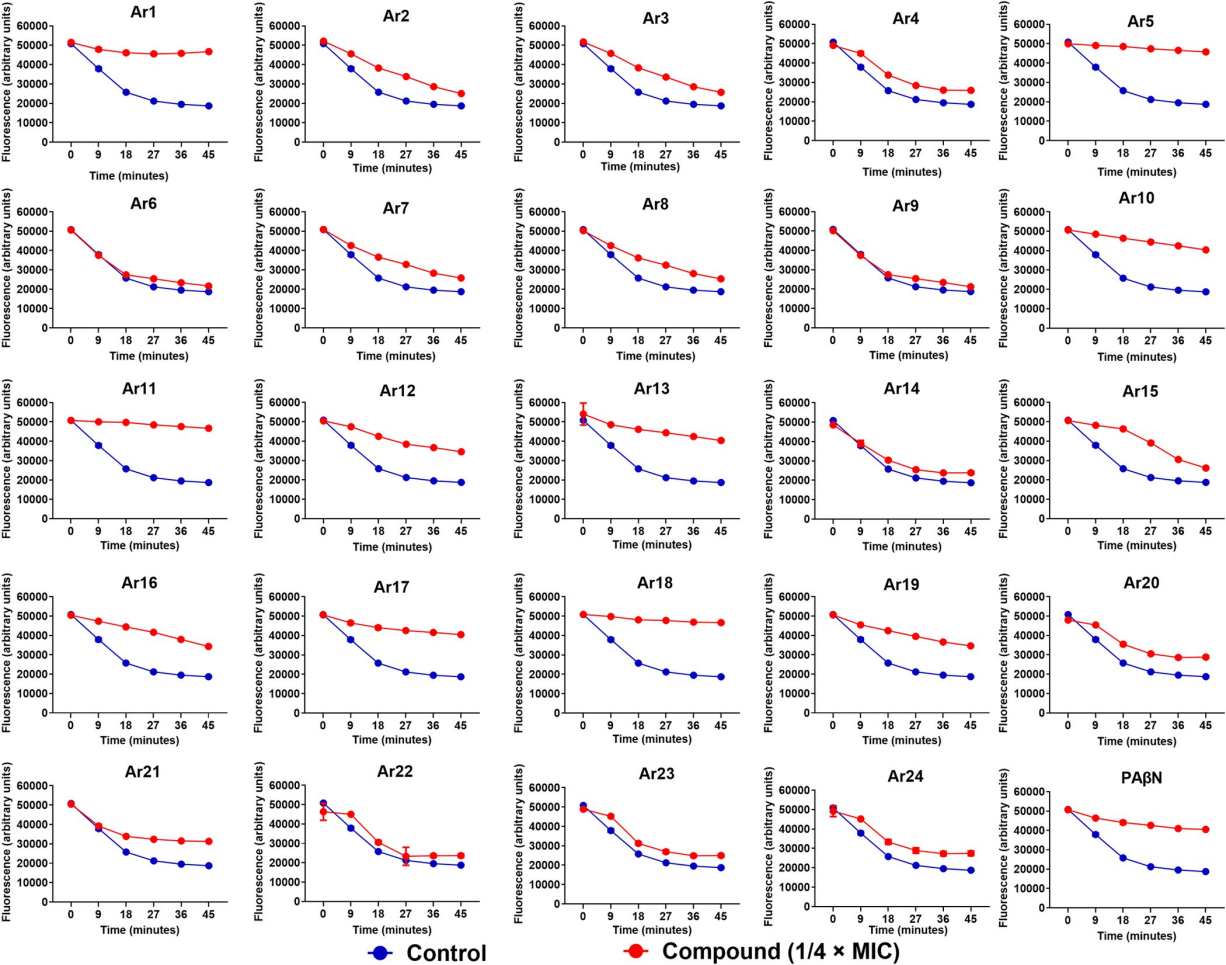
Effect of pyrrole derivatives (Ar1-Ar24) on Hoechst 33342 efflux in AcrB-TolC transporter-overproducing *E*. *coli* AG100_tet_ strain. AG100_tet_ cells were pre-loaded with Hoechst 33342 (2.5 μM) and either compounds (Ar1-Ar24 at 16 μg/mL) or PAβN (64 μg/mL, positive control) at ¼ × MIC under the condition that favor maximal accumulation. The data presented correspond to the average of triplicate readings ± SD.

Further, we determined the compounds’ potential to inhibit the Hoechst 33342 efflux in AcrB over-expressed *E*. *coli* AG100_tet_. The expression of AcrB efflux pumps caused a quick decrease in the fluorescence depicting Hoechst extrusion by the bacterial cells. In contrast, the presence of compounds at a sub-inhibitory concentration (1/4 × MIC) significantly delayed the decrease in fluorescence, indicating inhibited efflux compared to the untreated control. Our most efficient compounds were Ar1, Ar5, Ar11, and Ar18, exhibiting potent efflux inhibitory activity comparable to PAβN (Fig 3).

**Fig 4:**
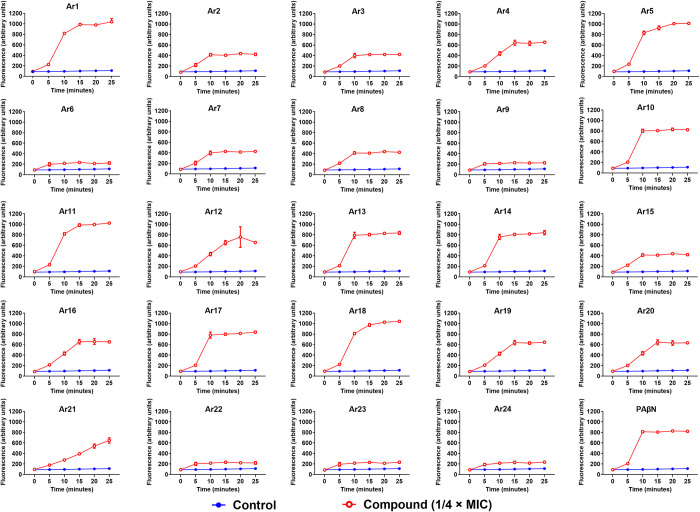
Accumulation of tetracycline (1 × MIC) in the presence of either Ar1-Ar24 at 1/4 × MIC (16 μg/mL) or PAβN at 1/4 × MIC (64 μg/mL) for 25 min in *E*. *coli* AG100_tet_. The increased accumulation of tetracycline in the bacterial cells was indicated by an augmentation in the tetracycline fluorescence intensity. The experiment was performed in triplicate, and the average data ± SD was plotted.

Additionally, we performed a tetracycline accumulation assay for a more realistic validation of the efflux inhibitory potential of the compounds. Tetracycline is known to be fluorescent, fluoresces with increased intensity as it traverses bacterial cell membrane [20, 21], and, most importantly, is a known substrate of RND efflux pumps [22]. We observed an increased accumulation of tetracycline in the presence of compounds (at ¼ × MIC) compared to the control. However, our four most efficient EPIs (Ar1, Ar5, Ar11, and Ar18) displayed a significant increase in fluorescence comparable to PAβN (Fig 4). Altogether, these results suggest that the compounds possess potent RND efflux pump inhibitory activity.

### Molecular docking and BLI studies identify the pyrrole derivatives as potential binders of AcrB and MexB

**Fig 5:**
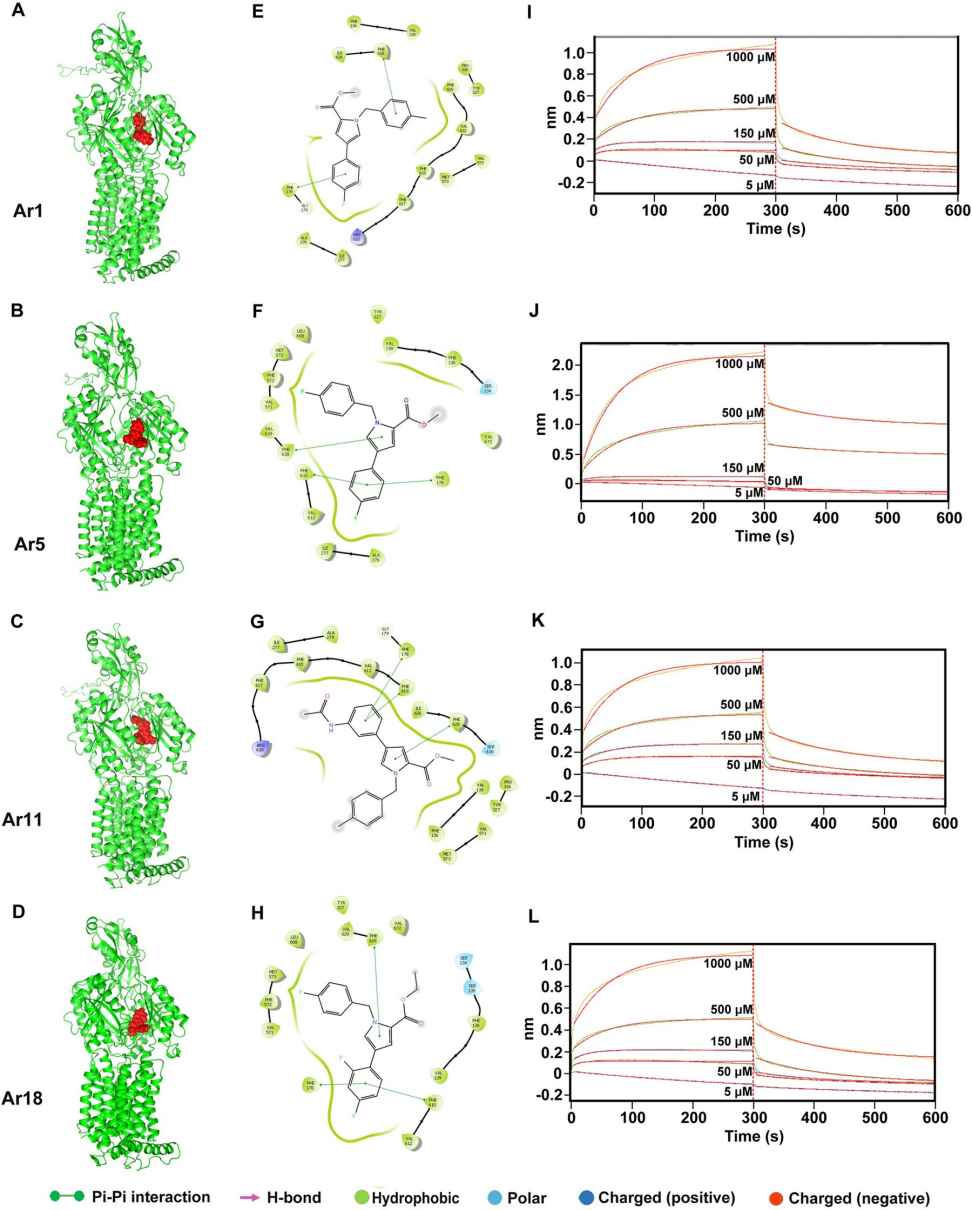
Structural representation of (A) Ar1 (B) Ar5 (C) Ar11 (D) Ar18 ligands (red spheres) docked in the active site cleft of AcrB (green cartoon). 2D ligand interaction diagram showing several interactions involved in binding of (E) Ar1 (F) Ar5 (G) Ar11 (H) Ar18 to AcrB. BLI study confirms the interaction between purified AcrB and EPIs (I) Ar1 (J) Ar5 (K) Ar11 (L) Ar18; the AcrB protein was immobilized onto an AR2G sensor tip, and interactions using different concentrations (ranging from 5 μM to 1 mM) of compounds were studied.

We performed *in silico* molecular docking studies to determine the binding residues involved in the interaction of compounds with AcrB and MexB proteins. The docking studies suggest that all four compounds (Ar1, Ar5, Ar11, Ar18) are docked at the substrate binding pocket and are stabilized by hydrophobic interactions, Pi–Pi stacking interactions, and/or H-bonding (Figs 5 and S2). This binding pocket is similar to the RP1 binding site, as reported in our previous study [17]. The AcrB/Ar and MexB/Ar binding pocket is lined by conserved hydrophobic patches. These hydrophobic patches are lined by multiple phenylalanine residues and collectively form a phenylalanine-rich substrate-binding pocket. The common phenylalanine residues that interact and stabilize all four compounds are Phe128, Phe178, Phe610, and Phe628 (Figs 5 and S2). The comparative analysis of AcrB and MexB binding with known inhibitor PAβN suggested that it also occupied the same site and shared common interacting residues (S3 Fig).

The docking scores of Ar compounds against AcrB and MexB range from -10.168 to -8.427 Kcal/mol, and the MMGBSA scores of Ar compounds against AcrB and MexB range from -67.08 to -56.18 Kcal/mol, indicating that these compounds have strong and stable interactions with the receptors (S6 Table).

According to our docking results and the available literature [3,23,24], we mutated selected residues at the distal binding pocket of AcrB to generate point and triple mutant variants. Two point variants (F178A and F628A) and one triple mutant (F615A, F617A, and R620A) variant of AcrB were created. *In silico* molecular docking studies were performed with the mutant proteins to predict the binding of Ar inhibitors. Interestingly, Ar inhibitors dock well in the binding site of all the mutants, suggesting these mutations do not affect binding. Ar compounds bind to the AcrB mutants at the same binding pocket as the wild type, suggesting no significant change in the binding site of the protein (S4 Fig). The docking scores of mutant proteins with Ar compounds ranged from -9.89 to -7.85, similar to that of the wild-type protein (S7 Table).

To study the interactions of AcrB with different pyrrole derivatives, we performed binding studies with recombinant AcrB using bio-layer interferometry (BLI). Our studies revealed that AcrB interacts with Ar1, Ar5, Ar11, and Ar18, as evident by the increase in the response (measured in nm) with increasing concentration. However, we could not determine the binding kinetics or K_d_ as the data did not fit adequately by applying the standard models (Fig 5I, 5J, 5K, and 5L). In our previous study, we performed the BLI studies using PAβN and CCCP as positive and negative controls, respectively. We observed the binding of AcrB with PAβN but not with CCCP [17].

Further, AcrB proteins from two point variants (F178A and F628A) and one triple mutant (F615A, F617A, and R620A) were purified and binding with Ar1, Ar5, Ar11, and Ar18 compounds was studied using BLI. We did not get good-quality data for the F628A variant. So, the data presented here suggests that mutating key residues in the binding pocket of AcrB did not affect the binding of Ar inhibitors (S5A and S5B Fig). It is noteworthy that the binding pocket of AcrB is having large volume that can facilitate the efflux of large antibiotics.

### EPIs sensitize bacterial populations to antibiotics

**Fig 6:**
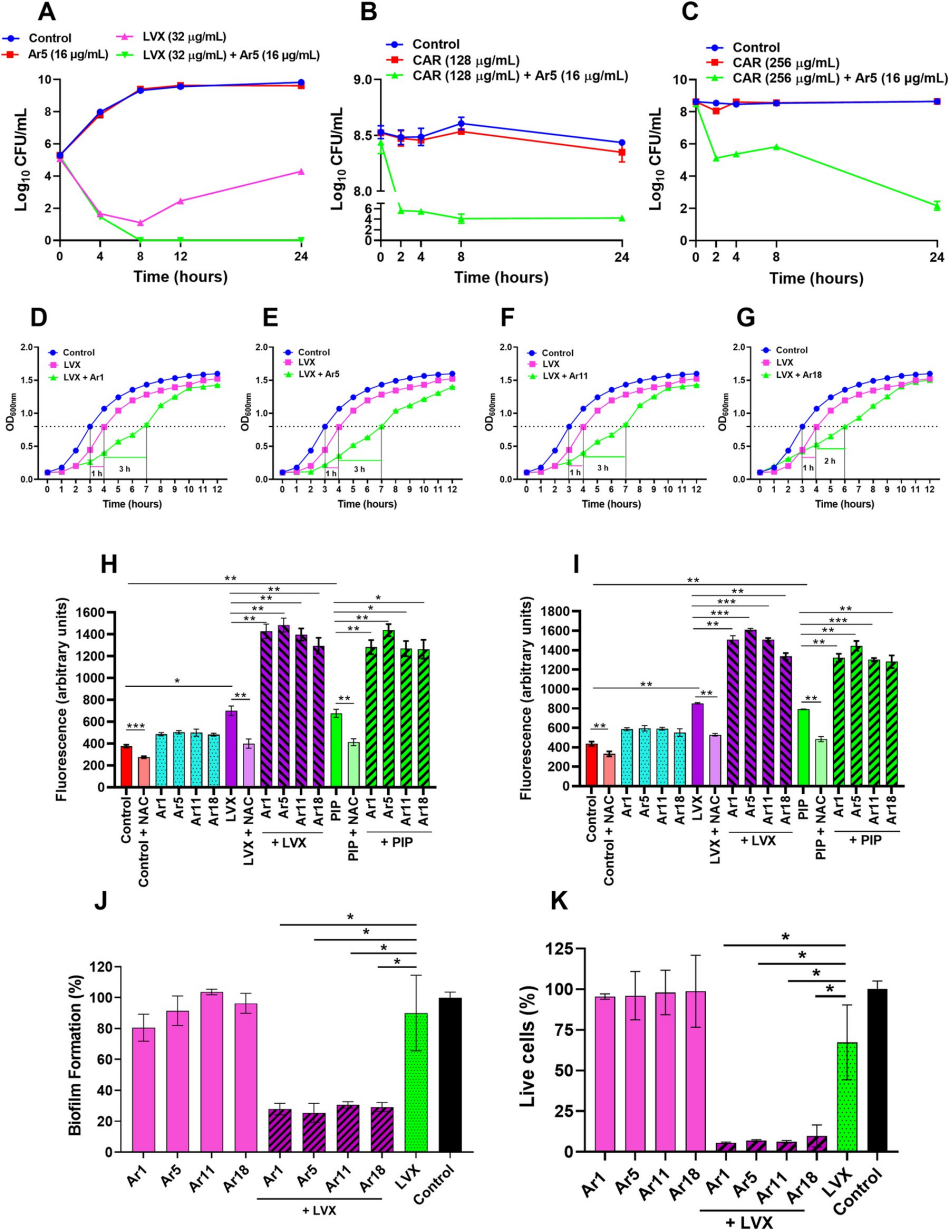
(A) Time-kill curves of *E*. *coli* ATCC BAA-2774 show levofloxacin’s bactericidal effect at 1 × MIC in combination with Ar5 at 16 μg/mL. Killing of (B) *E*. *coli* and (C) *P*. *aeruginosa* persister cells. The stationary phase cultures of *E*. *coli* AG100 and *P*. *aeruginosa* ATCC 27853 were treated with carbenicillin at 128 μg/mL and 256 μg/mL, respectively, for 18 h to isolate persister cells. These were re-exposed to carbenicillin in combination with Ar5 (16 μg/mL). Each time point in these experiments represents the mean log_10_ CFU/mL ± SD of three readings. Post-antibiotic effect (PAE) induced by levofloxacin in combination with (D) Ar1, (E) Ar5, (F) Ar11, and (G) Ar18 in *E*. *coli* ATCC BAA-2774 by the turbidimetry-based system. Growth curves of cultures not previously exposed (control) or pre-exposed to levofloxacin (1 × MIC) alone or in combination with EPIs at ¼ × MIC (16 μg/mL) for 2 h. Time 0 h corresponds to the growth monitoring immediately after antibiotic and EPI removal and cell resuspension in a fresh medium. PAE values in the graphs correspond to the growth halt undergone by the treated culture compared to the control in reaching an OD_600nm_ value half of the final OD_600nm_ after 12 h. The data represented is the average value of triplicates. Total ROS accumulation in (H) *E*. *coli* AG100tet and (I) *P*. *aeruginosa* ATCC BAA-2795 treated with levofloxacin (1 ×MIC) or piperacillin (1 ×MIC) in combination with EPIs (Ar1, Ar5, Ar11, and Ar18) at ¼ × MIC (16 μg/mL) for 2 h. Exogenous addition of NAC (6 mM) reduced the ROS accumulation. The average of triplicates ± SD is shown. (J) The influence of EPIs and levofloxacin on the preformed biofilms. The crystal violet assay assessed the biomass of *P*. *aeruginosa* ATCC BAA-2795 after exposure to sub-inhibitory concentrations (1/4 × MIC, 16 μg/mL) of EPIs and levofloxacin (1 × MIC) alone or in combination for 24 h. (K) Quantification of the effect of EPIs on mature biofilm in combination with levofloxacin. The viable bacterial population within the biofilm of *P*. *aeruginosa* ATCC BAA-2795 after 24 h exposure to sub-inhibitory concentrations (1/4 × MIC, 16 μg/mL) of EPIs and levofloxacin (1 × MIC) alone or in combination was determined using MTT assay. The results shown here correspond to the average ± SD of three independent readings. Results were considered significant when *p<0.05 and highly significant when **p<0.01 and ***p<0.001.

The kill-kinetics of *E*. *coli* ATCC BAA-2774 was performed to characterize the bactericidal effect of levofloxacin (1 × MIC) + Ar5 (16 μg/mL) combination. As expected, Ar5 alone (16 μg/mL) (lead EPI) behaved similarly to the untreated control and exhibited no antibacterial activity. Levofloxacin alone initially exhibited a reduction in bacterial CFU counts up to 8 h; however, re-growth was observed afterward. However, we observed that the combination of levofloxacin with Ar5 completely killed the bacteria in 8 h with no re-growth up to 24 h of incubation. Moreover, a remarkable reduction of 4.3 log_10_ CFU compared to levofloxacin alone was observed, representing synergy (Fig 6A).

Since bacterial persistence is directly correlated with efflux activity and negatively correlated with antibiotic accumulation [25], we anticipated and determined the effect of antibiotic and EPI combination against stationary phase antibiotic-induced *E*. *coli* and *P*. *aeruginosa* persisters. As expected, carbenicillin alone at 16 × MIC (128 μg/mL) and 8 × MIC (256 μg/mL) had a negligible killing effect against the *E*. *coli* and *P*. *aeruginosa* persister cells, respectively. The time-kill kinetics studies demonstrated the remarkable anti-persister potential of carbenicillin combined with Ar5, causing 4.2 log_10_ (Fig 6B) and 6 log_10_ (Fig 6C) reductions in the viable count against *E*. *coli* and *P*. *aeruginosa*, respectively. We further evaluated the efficacy of ciprofloxacin (2 × MIC, 4 × MIC, and 8 × MIC) in inhibiting *P*. *aeruginosa* persister cells and observed a typical biphasic killing pattern. These concentrations of ciprofloxacin could not display a complete bactericidal effect due to the dormant cells, i.e., persisters present in the bacterial population (S6A Fig). On the contrary, ciprofloxacin (8 × MIC) combined with Ar5 (16 μg/mL and 8 μg/mL) exhibited nearly complete eradication of the persister population within 3 h (S6B Fig). Moreover, the same MIC (0.25 μg/mL) values of ciprofloxacin observed each day after the re-growth procedure (against both control and ciprofloxacin-treated cells) provide evidence that the phenomenon we studied is persistence and not resistance to ciprofloxacin. These results suggest that the combination can be utilized as a therapeutic strategy against the persister cells.

The PAE of an antimicrobial agent is attributed to the effect that causes at least 0.5 h of halt in bacterial growth immediately after its removal [26]. The prolonged PAE of the antibiotics helps to maintain widely spaced dosing intervals. The PAE of levofloxacin against *E*. *coli* ATCC BAA-2774 was observed to be 1 h. In comparison, the combination treatment with Ar1, Ar5, Ar11, and Ar18 notably extended the PAEs of levofloxacin by 3 h (300%), 3 h (300%), 3 h (300%), and 2 h (200%), respectively (Fig 6D, 6E, 6F and 6G). Conclusively, the enhanced PAE in combination treatment would help the antibiotic to stay effective for a prolonged time against the target bacteria.

The excellent efficacy of the EPIs in kill-kinetics prompted us to evaluate their ability to prevent the emergence of spontaneous resistant mutants in response to levofloxacin. We observed that Ar5 at 16 μg/mL decreased levofloxacin’s mutation prevention concentration (MPC) by 32 folds, while each of Ar1, Ar11, and Ar18 (16 μg/mL) decreased the MPC by 16 folds. The potency of the combination in preventing resistant mutant development suggests its clinical relevance (Table 1).

**Table 1:** Mutation frequency of levofloxacin (with and without EPIs) on *K*. *pneumoniae* ATCC 700603. The experiment was performed in biological duplicates, results presented correspond to a single representative experiment.

Mutation Frequency with Levofloxacin								
Compounds	Conc. Used (μg/ml)	0.0625 × MIC	0.125 × MIC	0.25 × MIC	0.5 × MIC	1 × MIC	2 × MIC	4 × MIC
	0	UC	UC	UC	3.1 × 10^−10^	3.04 × 10^−12^	1.52 × 10^−12^	< 10^−11^
Ar 1	16	4.74 × 10^−10^	6.09 × 10^−11^	< 10^−11^	< 10^−11^	< 10^−11^	< 10^−11^	ND
Ar 5	16	3.96 × 10^−11^	< 10^−11^	< 10^−11^	< 10^−11^	< 10^−11^	< 10^−11^	ND
Ar 11	16	3.41 × 10^−10^	4.87 × 10^−11^	< 10^−11^	< 10^−11^	< 10^−11^	< 10^−11^	ND
Ar 18	16	3.85 × 10^−10^	5.79 × 10^−11^	< 10^−11^	< 10^−11^	< 10^−11^	< 10^−11^	ND

UC = Uncountable, ND = Not Determined

### EPIs enhance antibiotic-induced reactive oxygen species (ROS) production

Bactericidal antibiotics such as fluoroquinolones and β-lactams with diverse targets have been hypothesized to exhibit antibacterial effects partly by inducing ROS production. Notably, some bacterial efflux pumps are linked with ROS clearance, thereby protecting bacterial cells from damage [27]. So, we sought to determine the effect of EPIs on antibiotic-induced ROS generation in both *E*. *coli* AG100_tet_ (Fig 6H) and *P*. *aeruginosa* ATCC BAA-2795 (Fig 6I). Our lead EPIs (Ar1, Ar5, Ar11, and Ar18) at sub-inhibitory concentrations had a negligible effect on ROS production against *E*. *coli* and *P*. *aeruginosa*. Nonetheless, levofloxacin and piperacillin contributed to the endogenous ROS generation due to their DNA and cell wall damaging effects, respectively. Interestingly, the addition of EPIs significantly triggered the accumulation of antibiotic-induced ROS in these bacteria. We also observed that intracellular ROS generation was scavenged by an antioxidant N-acetyl cysteine (NAC). Taken together, we concluded that EPIs boosted the antibiotic-mediated ROS generation in *E*. *coli* and *P*. *aeruginosa* and can solve the classic issue of efflux pump-driven ROS tolerance.

### *P*. *aeruginosa* biofilms are disrupted by the EPI-antibiotic combination

The most common complications associated with *P*. *aeruginosa* infections are its ability to form biofilms, particularly in cystic fibrosis (CF) patients, contributing to morbidity and mortality. We evaluated the effect of our EPIs (Ar1, Ar5, Ar11, and Ar18) at a sub-inhibitory concentration (1/4 × MIC) combined with levofloxacin on the preformed *P*. *aeruginosa* biofilms. In the crystal violet (CV) assay, we observed that levofloxacin (1 × MIC) and EPIs (1/4 × MIC) alone did not significantly eradicate the biofilm. However, levofloxacin combined with Ar1, Ar5, Ar11, and Ar18 exhibited 72.1%, 74.7%, 69.5%, and 70.8% reduction in the preformed *P*. *aeruginosa* biofilms biomass, respectively, compared to control (Fig 6J). Based on the biofilm eradication potency displayed in the CV assay, we anticipated the effect of the combination on the viable bacterial population within biofilm by performing 4, 5-dimethylthiazol-2-yl)-2, 5-diphenyl tetrazolium bromide (MTT) colorimetric assay. We observed that EPIs alone at a sub-inhibitory concentration (1/4 × MIC) had a negligible effect, while levofloxacin alone (1 × MIC) exhibited a 33% reduction in the viability of bacteria within the biofilm. On the contrary, levofloxacin combined with EPIs displayed a notable reduction of 94.5%, 93.13%, 93.82%, and 90.33% in the live-cell population of preformed biofilm, respectively, compared to the control (Fig 6K).

### The EPIs do not disrupt bacterial membrane functions and behave neutrally to human calcium (Ca^2+^) channel activity

**Fig 7:**
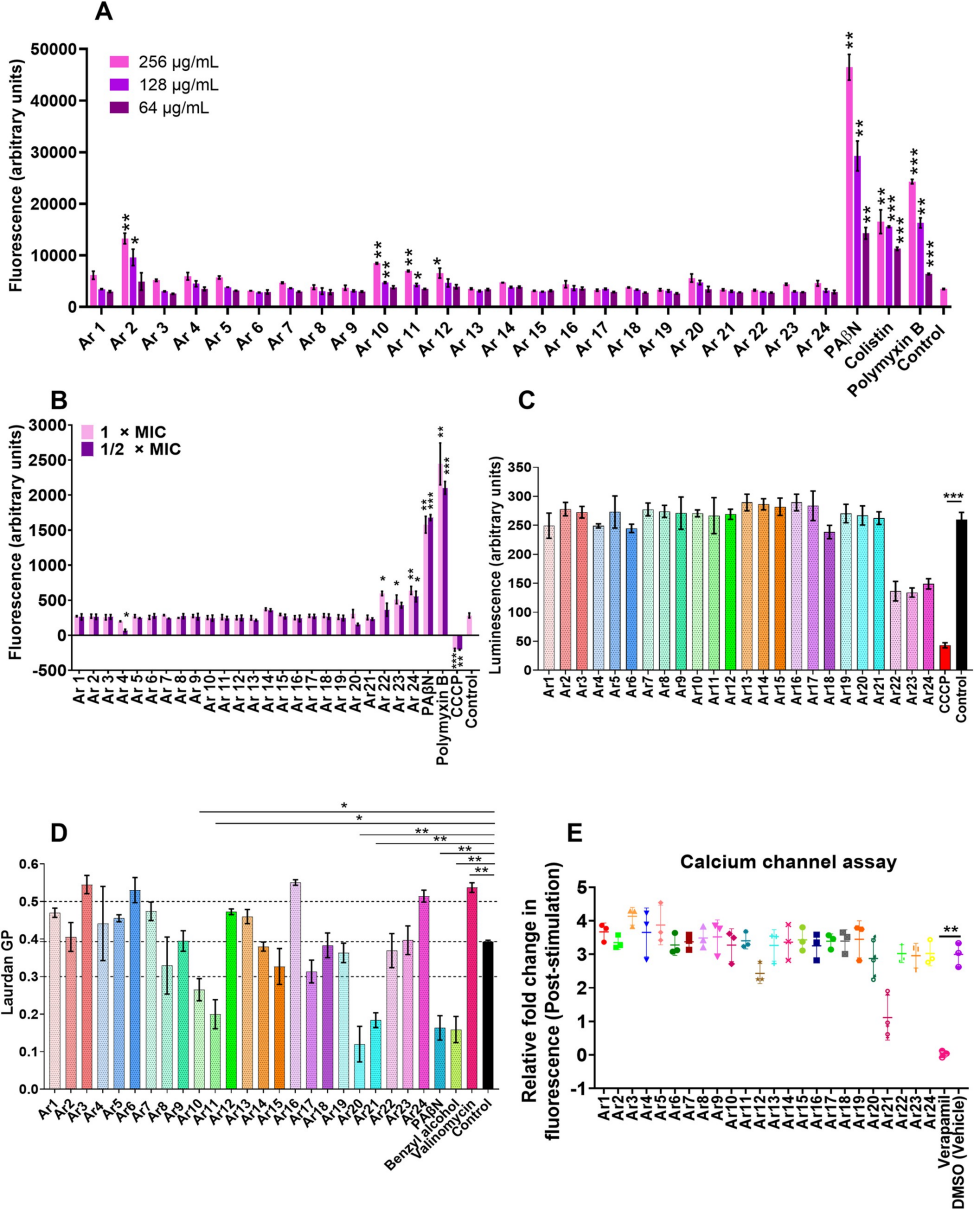
(A) Effect of EPIs (Ar1-Ar24) on the bacterial outer membrane permeability probed with ANS. *E*. *coli* AG100_tet_ was treated with ANS (3 mM) and compounds at (256 μg/mL, 128 μg/mL, 64 μg/mL) for 1 h at 37°C. Colistin (256 μg/mL, 128 μg/mL, 64 μg/mL) and PAβN (256 μg/mL, 128 μg/mL, 64 μg/mL), and polymyxin B (256 μg/mL, 128 μg/mL, 64 μg/mL) were included as positive controls. (B) Effect of EPIs (Ar1-Ar24) on the bacterial membrane depolarization probed with DiSC_3_(5). *E*. *coli* AG100_tet_ was treated with DiSC_3_(5) (0.4 μM) and compounds at either 1 × MIC (64 μg/mL) or ½ × MIC (32 μg/mL) for 60 min at 37°C. PAβN (64 μg/mL, 32 μg/mL), Polymyxin B (64 μg/mL, 32 μg/mL) and CCCP (64 μg/mL, 32 μg/mL) were included as a positive control. (C) Effect of EPIs (Ar1-Ar24) (16 μg/mL) on *E*. *coli* AG100_tet_ ATP levels. The bacterial culture was exposed to compounds or CCCP at ¼ × MIC (16 μg/mL) for 4 h. The ATP levels were quantified using a luciferin-luciferase bioluminescence detection assay. (D) Effect of EPIs (Ar1-Ar24) on the bacterial membrane fluidity probed with laurdan (10 μM). *E*. *coli* AG100_tet_ was treated with laurdan (10 μM) in the presence of compounds at 1 × MIC (64 μg/mL). PAβN (1 × MIC), valinomycin (50 μM), and benzyl alcohol (50 mM; a known membrane fluidizer) were included for comparison. The baseline fluorescence for each well was subtracted, and laurdan generalized polarization (GP) was plotted. (E) Mammalian calcium channel inhibition assay with HEK-293 cells. Cytoplasmic Ca^2+^ measures of carbachol pre- and post-treatment cells in the presence of either Ar1-Ar24 (16 μg/mL), DMSO (vehicle), or calcium channel antagonist verapamil (32 μg/mL). All the results presented here correspond to the average ± SD of triplicate readings. Results were considered significant when **p<0.01 and ***p<0.001.

Since the Gram-negative bacterial outer membrane acts as an effective permeability barrier, the synergy could also be observed due to the outer membrane permeabilization, causing the identification of false EPIs. We validated that the synergy observed is solely due to the efflux inhibition and not membrane targeting effects by performing an 8-anilino-1-napthylenesulfonic acid (ANS) assay with *E*. *coli* AG100_tet_. ANS is a neutrally charged fluorescent probe that fluoresces with increased intensity as it enters the hydrophobic environments [8]. The fluorescence increase was monitored for the compound-treated cells as a measure of membrane permeabilization. We did not observe any significant increase in the fluorescence for the *E*. *coli* AG100_tet_ treated with compounds (except Ar2, Ar10, Ar11, and Ar12). On the contrary, colistin (a known Gram-negative outer membrane permeabilizer), polymyxin B, and PAβN depicted significantly enhanced fluorescence compared to the control indicating membrane permeabilization (Fig 7A).

The RND family efflux pumps, i.e., AcrAB and MexAB, use proton motive force (PMF) for substrate transport around the inner membrane of bacteria. We assessed the effect of compounds on the inner membrane integrity to differentiate direct inhibition of AcrB from the PMF disruption by performing a 3, 3′-Dipropylthiadicarbocyanine iodide (DiSC_3_(5)) assay. DiSC_3_(5) is a fluorescent dye that fluoresces (by voltage-sensing) with increased intensity as it partitions the surface of the polarized cells. Depolarization of the membrane prevents dye partitioning, and bound dye is released into the media. The *E*. *coli* AG100_tet_ cells treated with compounds (except Ar22, Ar23, and Ar24) did not exhibit any significant fluorescence increase, indicating no PMF disruption. On the contrary, polymyxin B, CCCP, and PAβN treated cells exhibited a substantial change in fluorescence compared to control, indicating PMF disruption (Fig 7B).

Disruption of bacterial membrane functions can lead to reduced ATP levels due to respiratory chain impairment. To exclude the possibility of efflux inhibitory activity as a function of ATP depletion, we evaluated the ATP levels of *E*. *coli* AG100_tet_ cells treated with compounds at sub-inhibitory concentrations (1/4 × MIC). CCCP, a known ionophore, was included as a control due to its energy-dissipating nature. We observed no significant change (except Ar22, Ar23, and Ar24) in bacterial ATP levels in the cells treated (for 4 h) with compounds compared to the untreated control. However, the cells treated with CCCP displayed a sharp decrease in luminescence due to ATP depletion compared to control (Fig 7C).

Bacterial membrane fluidity is an important parameter in the action mechanism of membrane-targeting antibiotics [28], and some EPIs can alter the membrane fluidity, leading to false EPI identification. We evaluated the effect of EPIs (Ar1 –Ar24) on membrane fluidity by treating *E*. *coli* AG100_tet_ with EPIs at 1 × MIC using laurdan dye. Laurdan is a fluorescent probe; it intercalates into the membrane bilayer and detects membrane changes depending on surrounding water molecules, i.e., polarity in the membrane. Changes in the water content of the membrane cause wavelength shifts in laurdan emission, quantified by generalized polarization (GP) [29,30]. We observed that benzyl alcohol (known membrane fluidizer) and PAβN displayed significant alterations in membrane fluidity, making the membrane more fluid, while valinomycin was found to decrease membrane fluidity. In contrast, most EPIs (except Ar10, Ar11, Ar20, and Ar21) display negligible changes in bacterial membrane fluidity compared to the untreated control (Fig 7D). Most importantly, our potential EPIs (Ar1, Ar5, and Ar18) do not cause any alteration to essential bacterial membrane parameters or functions.

Another key parameter that limits the development of EPIs is their ability to interfere with the mammalian ion transport system. Verapamil is a well-known potent inhibitor of bacterial efflux pumps limited by its human Ca^2+^ channel inhibitory properties, causing human neurotoxicity [31]. Thus, we evaluated the effect of compounds on human Ca^2+^ channel activity. We stimulated the endoplasmic Ca^2+^ channels by adding carbamylcholine chloride (carbachol, a known calcium channel stimulator), which increases the Ca^2+^ levels in the cytoplasm. We observed that the addition of EPIs did not appear to significantly affect the human Ca^2+^ channel stimulation in response to carbachol. In contrast, verapamil caused a significant depletion of cytoplasmic Ca^2+^ ion accumulation, depicted by a drastic decrease in Fluo-4 dye fluorescence compared to control (Fig 7E). These results support the finding that the three potential EPIs (Ar1, Ar5, and Ar18) identified in the study exhibit synergy due to true efflux pump inhibition and no membrane-targeting effects. However, it is important to mention that EPIs at higher concentrations might behave differently, and the effects would need to be resolved for eventual *in vivo* use.

### EPIs lack toxicity in mammalian cells

Considering the lack of effect of EPIs on eukaryotic Ca^2+^ ion channels, we further evaluated cytotoxicity on Human Peripheral Blood Mononuclear cells (H-PBMC). In so doing, we observed the half-maximal inhibition concentration (IC_50_) of the compounds to be ˃128 μg/mL and a corresponding safety index of ˃8 following 24 h of incubation in the presence of EPIs (256 μg/mL—64 μg/mL). Our front runners, Ar1, Ar5, and Ar11, had extremely low toxicity toward H-PBMCs. Of particular interest, our best EPI (Ar5) exhibited IC_50_ at 256 μg/mL, corresponding to a safety index of 16 (S8 Table). We further performed a hemolysis experiment and observed no significant lysis and hemoglobin release for the rabbit red blood cells incubated with our most active compounds (Ar1, Ar5, and Ar18). We observed that the HC_10_ (the concentration at which 10% hemolysis occurs) of EPIs is >64 μg/mL—>512 μg/mL and a hemolysis index of 4–32. Nonetheless, our best EPI (Ar5) exhibited no hemolysis at 512 μg/mL (HC_10_ of ˃512 μg/mL) and a hemolysis index of ˃32. Triton X-100 (0.1%) was included as a positive control and exhibited 100% hemolysis (S9 Table). Taken together, these experiments provide substantial evidence that Ar5 is non-toxic *in vitro* and can be used for therapeutic applications *in vivo*.

### The EPIs exhibit anti-virulence potency and protect *C*. *elegans* from *P*. *aeruginosa* virulence

**Fig 8:**
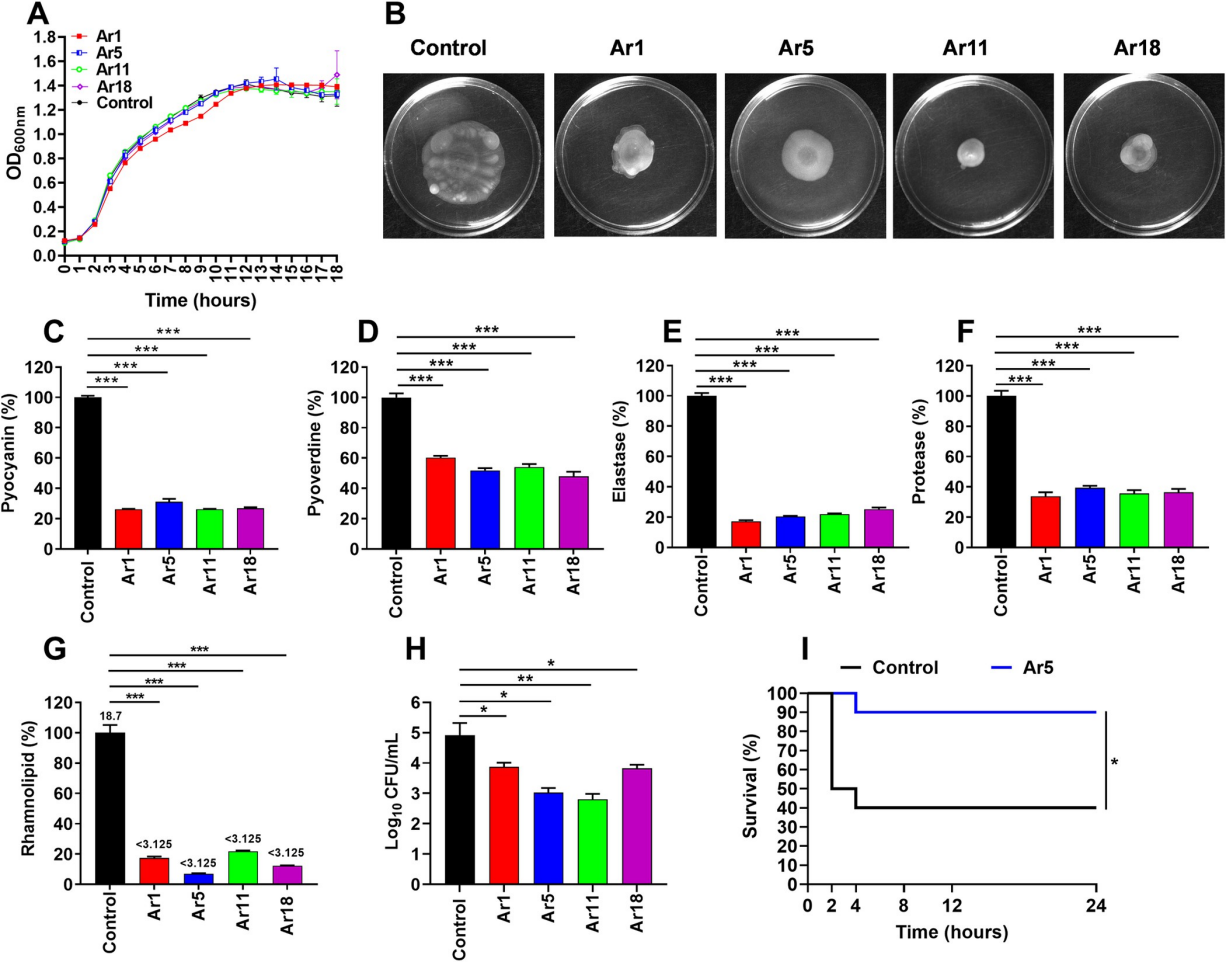
(A) Growth kinetics of *P*. *aeruginosa* PAO1 strain in CA-MHB under sub-inhibitory concentrations (1/4 × MIC (16 μg/mL)) of EPIs (Ar1, Ar5, Ar11, and Ar18). OD_600nm_ values are the means ± SD from three independent readings. (B) Effect of EPIs (Ar1, Ar5, Ar11, and Ar18) on *P*. *aeruginosa* PAO1 swimming motility. For treatment conditions, the plates were supplemented with Ar1, Ar5, Ar11, and Ar18 at sub-inhibitory concentrations (16 μg/mL) and incubated for 24 h at 37°C. The control plates were supplemented with an equal volume of DMSO as in treatment plates. Effect of EPIs (Ar1, Ar5, Ar11, and Ar18) on the production of extracellular virulence factors; pyocyanin (C), pyoverdine (D), elastase (E), protease (F), and rhamnolipids (G) levels in the culture supernatants of *P*. *aeruginosa* PAO1 in the presence of sub-inhibitory concentrations of EPIs (16 μg/mL). The percentage values were calculated with respect to the bacterial culture without treatment. The results were represented as mean ± SD of three independent readings. (H) Influence of sub-inhibitory concentrations (16 μg/mL) of EPIs (Ar1, Ar5, Ar11, and Ar18) on the invasive capacities of *P*. *aeruginosa* PAO1 within RAW264.7 macrophages. The results were presented as mean ± SD log_10_ CFU/mL of three independent readings. (I) Ar5 inhibits *P*. *aeruginosa* PAO1 virulence toward *C*. *elegans*. *C*. *elegans* were initially applied to *E*. *coli* OP50 lawns for feeding, and then the L4 stage worms were transferred to lawns of *P*. *aeruginosa* PAO1 grown (for 18 h) in the presence or absence of Ar5 (16 μg/mL). The control (without treatment) plates were supplemented with an equivalent volume of DMSO (vehicle). For survival assay, 10 L4 worms were placed in triplicate plates for each experimental condition. All the results were considered significant when *p<0.05 and highly significant when **p<0.01 and ***p<0.001.

*P*. *aeruginosa* is known to produce a multitude of virulence factors that aid the bacteria in its pathogenicity. Since earlier evidences suggest the role of efflux pumps in virulence, EPIs might also affect the virulence of bacteria besides enhancing antibiotic activity. So, we evaluated the effect of our four most active EPIs (Ar1, Ar5, Ar11, and Ar18) at sub-inhibitory concentrations (16 μg/mL) on the inhibition of motility and virulence factors of *P*. *aeruginosa* PAO1 (expressing Mex efflux pumps). Firstly, we assessed if the compounds at sub-inhibitory concentrations (16 μg/mL) exhibited any inhibitory effects on primitive bacterial growth pattern for 18 h and observed that none of the compounds (Ar1, Ar5, Ar11, and Ar18) altered the typical growth pattern of bacteria. As desired, it did not significantly differ from the control (without treatment) group (Fig 8A). Since motility is considered crucial for the establishment of *P*. *aeruginosa* infection, we determined the effect of the EPIs in restricting its surface motility. We observed a remarkable difference in the swimming motility for the treatment conditions compared to the control (Fig 8B), while a little inhibitory effect was observed for the swarming motility (S7A Fig). However, we observed no difference in the twitching motilities of treatment sets compared to the control (S7B Fig). Pyocyanin is a blue redox-active toxin, an important virulence factor of *P*. *aeruginosa* known to interfere with multiple cellular functions [32]. We observed a marked decrease in the pyocyanin levels of cells treated with EPIs, exhibiting ≥70% reduction as compared to the control (without treatment) (Fig 8C). Pyoverdine is a known siderophore and acts as an iron-chelator, promoting biofilm formation in *P*. *aeruginosa* [32]. Our EPIs demonstrated a ≥ 40% reduction in the pyoverdine levels compared to the untreated control (Fig 8D). *P*. *aeruginosa* elastases contribute to the bacterial invasion of lung parenchyma by cleaving multiple proteins, including fibrinogen, collagen, and elastin [32]. We observed a significant decrease in the elastase levels of EPI-treated cells, exhibiting ≥75% reduction as compared to the control (without treatment) (Fig 8E). *P*. *aeruginosa* proteases are known to degrade host iron-binding proteins and other immune system proteins to prevent bacterial clearance [32]. We observed a ≥60% reduction in protease levels in the cells treated with EPIs compared to control (Fig 8F). Another important virulence factor, rhamnolipids, is a biosurfactant and possesses hemolytic potential. We observed a significant decrease in the rhamnolipid levels of EPI-treated cells, exhibiting ≥79% reduction as compared to the control (without treatment) (Fig 8G). To support our hypothesis that the anti-virulence effects we observed are due to secondary effects of efflux pump inhibition, we tested the levels of major virulence factors on *P*. *aeruginosa* PAO750 (Δ*mexAB-oprM*, Δ*mexCD-oprJ*, Δ*mexEF-oprN*, Δ*mexXY*). However, we did not observe any reduction in *P*. *aeruginosa* PAO750 swimming, swarming, and twitching motility, unlike PAO1, which can be interpreted as a lack of activity due to Mex efflux pump deletion in PAO750 (S7C, S7D and S7E Fig). Moreover, we observed that none of the EPIs displayed any significant reduction in the levels of virulence factors (S8A–S8E Fig). These observations partly support our hypothesis that the anti-virulence effect we observed is due to Mex efflux pump inhibitory activity of the EPIs.

The phenotypic inhibition of virulence-related traits raises the question of what kind of effect Ar5 has on *P*. *aeruginosa* PAO1 transcriptome. To tackle this issue, the transcriptional profile of *P*. *aeruginosa* PAO1 in the presence and absence of sub-inhibitory concentration of Ar5 were compared by means of RNAseq analysis. PAβN was included for comparison (S9A and S9B Fig). Interestingly, we did not observe any significant up-regulation or down-regulation in the expression of virulence factor genes compared with the untreated control, indicating that the effect of Ar5 on these phenotypes may depend upon efflux pump inhibition (S10 Table). Since quorum sensing (QS) is known to play a key role in modulating the expression of virulence genes in *Pseudomonas aeruginosa* [33], we determined the effect of the treatment of Ar5 on the gene expression of three QS systems of *Pseudomonas aeruginosa*. We quantified the expression of lasI, lasR (lasI-lasR QS system), rhlI, rhlR (rhlI-rhlR QS system), pqsA, pqsB, pqsC, pqsD, pqsE, pqsH, pqsR (PQS QS system), and qscR (regulator of las QS). We did not observe any significant up-regulation or down-regulation in the expression of these genes compared to the untreated control (S10 Fig). These data further support our hypothesis that the anti-virulence effect of pyrrole compounds may depend on efflux pump inhibition.

The *in vitro* anti-virulence potency of the EPIs aroused our curiosity to test if the effect could also extend *in vivo*, and we performed the *C*. *elegans* survival assay. We observed that only 40% of worms survived from the worms fed on *P*. *aeruginosa* untreated control plates for 24 h. However, 90% of the worms fed on *P*. *aeruginosa* treated with Ar5 survived (Fig 8I). The enhanced survival of the worms in the treatment group can be attributed to the reduced levels of various virulence factors, which reduced *P*. *aeruginosa* pathogenicity. In the validation experiment, we observed that 90% of worms survived *P*. *aeruginosa* PAO750 infection both in the untreated control and Ar5 treated sets, indicating the lack of anti-virulence activity due to the absence of Mex pumps (S8F Fig). Since RND pumps are also known for their activity in bile acid resistance, *C*. *elegans* might also have bile acids in their digestive tract. Also, EPI PAβN has been observed to sensitize *P*. *aeruginosa* PAO1 to bile salts. To rule out the possibility that the synergistic activity of Ar5 with bile salts might result in false-positive anti-virulence activity, we looked for any reduction in CFU in the presence of Ar5 with bile salts. However, we did not observe any CFU reduction for Ar5/Bile salt combination together, whereas PAβN sensitized PAO1 to bile salts, and a reduction in CFU was observed (S11 Fig). Altogether, these observations suggest that the anti-virulence activity of EPIs is attributed to a secondary effect of efflux inhibition.

### The EPIs hampered bacterial invasion into macrophages

The ability of bacteria to invade the mammalian cells makes them virulent, contributes to establishing bacterial infection, and promotes disease. Alongside assessing the role of EPIs as putative anti-virulence agents in virulence factor inhibition assays, we assessed the role of our four anti-virulent compounds (Ar1, Ar5, Ar11, and Ar18) in reducing the invasiveness of *P*. *aeruginosa* PAO1. We observed that the treatment of Ar1, Ar5, Ar11, and Ar18 reduced the invasion of PAO1 by 1.1 log_10_, 1.9 log_10_, 2.1 log_10_, and 1.1 log_10,_ respectively, compared to the control (Fig 8H).

**Fig 9:**
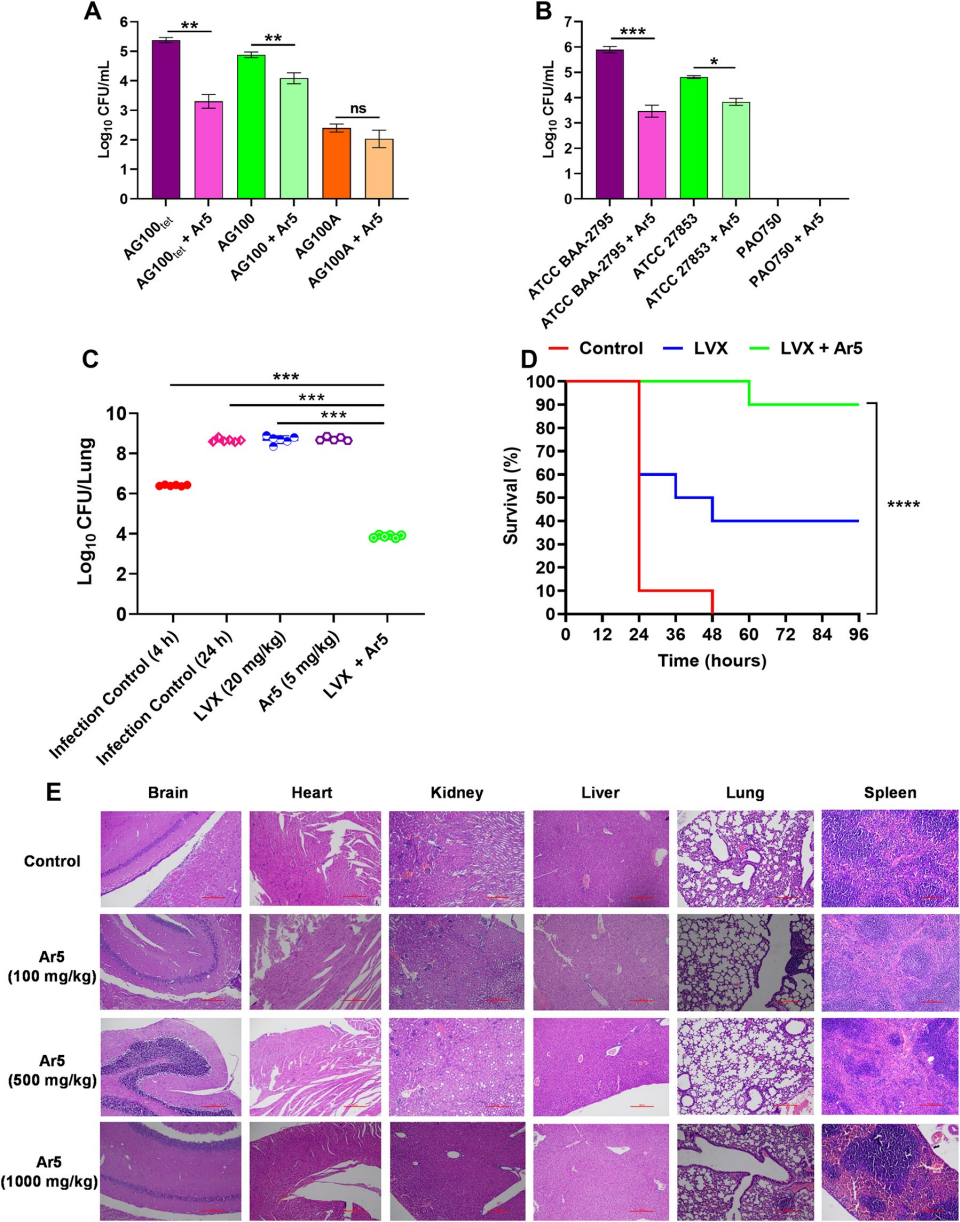
Influence of Ar5 (16 μg/mL) on the invasive capacities of (A) *E*. *coli* wild-type AG100, AcrB-TolC overproducing AG100_tet_, and AcrAB knockout AG100A; (B) *P*. *aeruginosa* wild-type ATCC 27853, MexAB-OprM overproducing ATCC BAA-2795, and PAO750 with MexAB deletion within RAW264.7 macrophages. The results were presented as mean ± SD log_10_ CFU/mL of three independent readings. (C) Ar5-levofloxacin combination is efficacious in mice. Single-dose (subcutaneous; 4 h post-infection, 6 mice/group) treatment with Ar5 (5 mg/kg) and levofloxacin (20 mg/kg) alone or in combination in immunocompetent lung infection model using *P*. *aeruginosa* ATCC BAA-2795. For drug-treated animals, lungs CFUs were determined at 24 h post-infection. For controls, CFU in the lungs was determined 4 h and 24 h post-infection. The CFU from each mouse is plotted as individual points, and error bars represent the SD within an experimental group. (D) For the peritonitis survival model, mice (*n* = 10 per group) were injected with a lethal dose of *P*. *aeruginosa* ATCC BAA-2795 and treated with three repetitive dosing of Ar5 (5 mg/kg), levofloxacin (20 mg/kg), and their combination 1, 4, and 7 h post-infection subcutaneously. The percent survival was calculated and represented using the Kaplan-Meier survival plot. Results were considered significant when *p<0.05 and highly significant when **p<0.01, ***p<0.001, and ****p<0.0001. (E) Histopathology analyses of brain, heart, lung, spleen, liver, and kidney of mice when treated with vehicle (control) or Ar5 (100, 500, and 1000 mg/kg). After removal, the organs were fixed in buffered formalin and stained with hematoxylin and eosin.

Further, we also evaluated the role of our best EPI (Ar5) in reducing the invasion of both *E*. *coli* and *P*. *aeruginosa* in human macrophage cells. We evaluated the invasive potential of *E*. *coli* AG100_tet_ (AcrB-overproducing), AG100 (wild-type), and AG100A (AcrAB-deletion). We observed that *E*. *coli* invasion was 0.5 log_10_ higher in the AcrB-overproducing strain and 2.5 log_10_ lower in the AcrAB-deletion strain compared to the wild-type strain. We observed that our lead EPI, Ar5 reduced the invasive abilities of AcrB-overproducing and wild-type strains into macrophages by 2 log_10_ and 0.8 log_10,_ respectively, compared to the corresponding controls (without treatment) (Fig 9A). The invasiveness of AcrB-overproducing *E*. *coli* AG100_tet_ was reduced to a greater extent as compared to the wild-type *E*. *coli* AG100.

Likewise, we evaluated the bacterial invasive abilities of the *P*. *aeruginosa* ATCC BAA-2795 (MexAB-OprM overproducing), ATCC 27853 (wild-type), and PAO750 (deletion strain) in macrophages. We observed that *P*. *aeruginosa* invasion was 1 log_10_ higher for the MexAB-overproducing strain than the wild-type strain; on the contrary, *P*. *aeruginosa* PAO750, with the Δ*mexAB-oprM*, Δ*mexCD-oprJ*, Δ*mexEF-oprN*, Δ*mexXY* genes deleted, failed to penetrate macrophage cells. Our lead EPI, Ar5, reduced the bacterial invasion of MexAB-overproducing and wild-type strains into macrophage cells by 2.4 log_10_ and 1 log_10_, respectively, compared to the corresponding controls (without treatment) (Fig 9B).

### The combination cured lung infection and protected sepsis-induced mice

The combination’s extensively promising abilities prompted us to evaluate its *in vivo* potential to cure *P*. *aeruginosa* infections. Since *P*. *aeruginosa* is the predominant cause of lung infections, particularly in CF patients, we established a neutropenic murine lung infection model and assessed the efficacy of the levofloxacin + Ar5 combination to cure it. Neither levofloxacin (20 mg/kg) nor Ar5 (5 mg/kg) alone significantly reduced bacterial counts. On the contrary, the levofloxacin-Ar5 combination demonstrated remarkable potency, resulting in a 2.5 and 4.7 log_10_ CFU reduction compared to the untreated control at 4 h and 24 h, respectively (Fig 9C). Further, we evaluated the potential of the combination to protect sepsis-induced mice against *P*. *aeruginosa* infection in the murine peritonitis survival model. We observed that only 10% of mice survived in the control group up to 48 h, and 90% died within 24 h of infection. The group with levofloxacin treatment (20 mg/kg; 3 doses) exhibited 40% increased survival rates compared to the control. In contrast, 90% of the mice survived after a three-dose treatment of levofloxacin + Ar5 (20 mg/kg + 5 mg/kg) 5 days post-infection, corresponding to a 90% increase in survival rate compared to control. The combination’s remarkable *in vivo* therapeutic potential highlights its clinical utility (Fig 9D).

### Ar5 appears to be safe in acute toxicity studies

The classical toxicity issues of EPIs are the major challenges that lead to their failure to get approval and reach the market. Keeping it in concern, as a preliminary toxicity evaluation, we first compared the acute toxicity of Ar5 with that of the control group. BALB/c mice were injected subcutaneously with different amounts of Ar5, in single-dose, in the range of 100–1000 mg/kg, and were monitored for 24 h post-administration of the compound. We did not observe any behavioral changes, morbidity, or mortality in the mice dosed with different concentrations of Ar5 with respect to the control group. Moreover, the analyses of histopathology studies of six major organs (brain, heart, lungs, liver, spleen, and kidney) showed no signs of toxicity similar to the control group (Fig 9E). Nonetheless, we also assessed the effect of Ar5 on blood biochemical parameters, including creatinine, triglycerides, cholesterol, bilirubin (conjugated and unconjugated), alanine aminotransferase (ALT/SGPT), alkaline phosphatase (ALP), aspartate aminotransferase (AST/SGOT), total proteins, albumin, and globulin. Additionally, we assessed the change in blood glucose and body weight before and after treatment (S11 Table). We did not observe any notable difference in these biochemical parameters compared to the control group. Based on acute toxicity studies, Ar5 can be considered non-toxic and safe for further clinical studies.

### *In silico* absorption, distribution, metabolism, and excretion (ADME) analyses

*In silico* ADME analyses of all the compounds revealed high intestinal absorption, moderate aqueous solubility, and blood-brain barrier penetration abilities (S12 Table). The bioavailability score of all the compounds was found to be better than PAβN and reserpine. Our best hits, Ar1, Ar5, and Ar11, did not violate Lipinski’s rule of drugability. Unlike PAβN, our lead EPI (Ar5) did not inhibit cytochrome P450 activity. In sum, most EPIs display favorable pharmacokinetic properties, a drug-like nature, and medicinal chemistry friendliness.

## Discussion

MDR Gram-negative bacterial infections are a significant cause of global health concern. The RND family of efflux pumps plays a crucial role in developing innate and acquired resistance in Gram-negative bacteria by extruding multiple classes of antibiotics to the cell’s exterior. Developing new efflux pump inhibitors targeting these efflux pumps is deemed desirable to turn the tide against Gram-negative drug-resistant bacteria. The EPI-mediated combination therapy would help increase antibiotic efficacy and revive obsolete antibiotics. Moreover, retarded antibiotic efflux would increase the hit rates in drug discovery, where over-expression of RND pumps is the reason for low hit rates [34]. Multiple inhibitors targeting RND-efflux pumps have been discovered previously; nonetheless, none could proceed to clinical trials due to their unavoidable toxicities. The need of the time is to look for new EPIs with maximal efficacies and minimal toxicities.

Since the pyrrole scaffold is previously validated for the RND efflux pump inhibition [17,18], we synthesized pyrrole derivatives targeting the RND efflux system, i.e., AcrAB-TolC of *Enterobacteriaceae*, MexAB-OprM of *P*. *aeruginosa*. As required, the synthesized derivatives did not display any intrinsic antibacterial activities at the used concentrations; otherwise, bacteria may develop resistance to them. Nonetheless, the compounds potentiated the MIC of the tested antibiotics many folds in efflux pump over-expressing strains of *E*. *coli*, *K*. *pneumoniae*, and *P*. *aeruginosa* in a concentration-dependent manner. Moreover, our four most active compounds (Ar1, Ar5, Ar11, Ar18) reduced tetracycline’s MIC below the clinical susceptibility breakpoints (≤4 μg/mL) [35] and exhibited better potentiation activity than PAβN (a known EPI). However, we did not observe any potentiation in the corresponding deletion strains due to the absence of the efflux pumps, suggesting the specificity of compounds for the RND efflux pumps. We further validated the efflux pump inhibition through Hoechst efflux assays, which are considered an indication of EPI functionality. The compounds enhanced the accumulation of Hoechst 33342 and retarded its extrusion in the AcrB over-expressed *E*. *coli*. Likewise, we observed a similar enhancement in tetracycline accumulation, which can directly correlate with the increase in tetracycline concentration within the bacterial cell. The enhanced accumulation further strengthens our finding that the compounds are RND efflux pump inhibitors.

Further, the BLI and molecular docking-based studies provided insights into the mechanism of interaction of AcrB with the inhibitors. We observed that all the inhibitors dock at the same binding pocket to inhibit the AcrB. Although we were not able to obtain the binding kinetics of inhibitors using BLI, but our data provides an experimental evidence for the interaction of AcrB with inhibitors. To confirm that Ar compounds target the distal binding pocket as predicted by docking studies, we did site-directed mutagenesis of the AcrB protein. Considering our docking results and literature, we selected important phenylalanine residues of the distal binding pocket, including F178, F615, F617, R620, and F628, which were also shown earlier to be critical for the binding of substrates and EPIs [3,36,37]. Site-directed mutagenesis generated AcrB^F178A^, AcrB^F628A^, and AcrB^F615A, F617A, R620A^ protein mutants for our study, which were further used for BLI to analyze the binding of our inhibitors. Our results indicated that even after the mutations in the distal binding pocket, the inhibitors do bind to the protein, showing that the hydrophobic region of the protein was not altered much because of these mutations. Likewise, docking of the Ar compounds to the mutated protein also predicted the binding of the compounds at the same site as that of the wild-type protein. The docking results are in agreement with the experimental data presented here. Overall, the BLI and docking studies indicate that the Ar compounds bind to the distal binding pocket of the AcrB protein, and mutating a few residues in a large channel (suitable for efflux of large molecules like antibiotics) does not impede the binding of the compounds.

In time-kill kinetics, synergy is defined as reducing 2 log_10_ CFU/mL in combination compared to its most active counterpart (antibiotic here) and must be ≥2 log_10_ CFU/mL below the starting inoculums [38]. We observed 4.3 log_10_ reductions in CFU for the levofloxacin-Ar5 combination compared to levofloxacin alone, representing synergy. Since persistence is correlated with efflux pumps and some previous studies report that EPIs, e.g., NMP and PAβN, enhanced antibiotic’s lethal effect against persister cells [25], we hypothesized that our EPIs might decrease the persister cells population. True to our hypothesis, we observed a remarkable decline in *E*. *coli* and *P*. *aeruginosa* persister cell populations for the combination treatment of Ar5 combined with carbenicillin and ciprofloxacin, respectively. Taken together, the combination can be employed to treat acute as well as chronic bacterial infections. Additionally, our EPI-antibiotic combinations remarkably enhanced the PAE of levofloxacin by ≥2 h. The enhanced PAEs of the antibiotics help sustain widely spaced dosing intervals. Most importantly, an EPI does not need to be always present to retain the antibiotic sensitivity of bacteria. The bactericidal antibiotics work by generating endogenous ROS [27]. Since an EPI does not target essential bacterial processes or display antibacterial activity itself but revives antibiotic sensitivity by targeting the resistance mechanism against the antibiotic, the combination treatment can lessen the resistance development [39–41]. Consistent with the hypothesis, we observed a diminished resistant mutant development frequency after the combination treatment of EPI with levofloxacin. The MPC of the combination was lower than or equal to the C_max_ of levofloxacin (2.8–5.2 μg/mL) in human plasma [42], indicating the clinical value of these combinations in restricting the development of resistant mutants.

Since efflux pumps are known to deplete the antibiotic-generated ROS through their extrusion, inhibition of efflux pumps can benefit attenuate ROS clearance. Consistent with the notion, we observed an increased accumulation of antibiotic-induced endogenous ROS for the combination treatment, indicating retarded depletion of ROS through inhibited efflux pumps. Ultimately, retarded ROS depletion/increased ROS accumulation due to efflux inhibition will result in the potentiation of the antibiotic’s antibacterial activity. One important determinant of chronic *P*. *aeruginosa* infections is its ability to form robust biofilms, which is further aided by the over-expression of efflux pumps [43]. The combination treatment of levofloxacin and EPIs significantly reduced preformed biofilms alongside reducing the viability of biofilm-enclosed bacterial cells.

Ideally, an EPI should not disrupt bacterial membrane functions. Alteration of bacterial membrane functions displays efflux inhibitory activity as a secondary effect, which leads to antibacterial activity by compounds alone and detection of non-specific pseudo-EPIs [8,44,45]. Many EPIs discovered previously possess undesired membrane-targeting properties, leading to their clinical ineffectiveness [19,46]. We observed that our lead EPIs possess none of the bacterial membrane targeting properties, unlike the previously discovered EPI PAβN [8]. The EPIs are devoid of membrane depolarisation and ATP depletory properties, unlike PAβN, colistin, polymyxin B, and CCCP. Another reason for the failure of EPIs is mammalian Ca^2+^ channel inhibitory activities, and one such example is that of verapamil exhibiting Ca^2+^ channel inhibitory properties eliciting neurotoxicity [31,39]. Our EPIs do not interfere with human Ca^2+^ channels and are devoid of related toxicities.

The extensive potential of *P*. *aeruginosa* to produce a plethora of virulence factors aids in its pathogenicity. The emerging evidence suggests RND transporters’ involvement in the extrusion of bacterial factors considered necessary for virulence [11,12,47,48]. We hypothesized that EPIs might attenuate bacterial virulence as a secondary effect of efflux inhibition. Consistent with our hypothesis, we observed that EPIs declined virulence factor levels, including pyocyanin, pyoverdine, elastase, protease, rhamnolipids, and retarded swimming motility. The anti-virulence effect of EPIs also extended to protecting *C*. *elegans* against *P*. *aeruginosa* infection, where we observed enhanced survival of nematode larvae for the treatment conditions. In *P*. *aeruginosa* PAO1, the *in vivo* protection effect exhibited by Ar5 well corroborates with *in vitro* suppression of virulence factor levels. Moreover, the fact that EPIs reduce virulence in Mex-expressing *P*. *aeruginosa* PAO1 and not in Mex-deletion PAO750 indicates that EPIs display anti-virulence activities due to the secondary effects of efflux pump inhibition. The RNAseq and qRT-PCR analysis suggested that EPIs can directly block the export of virulence determinants and reduce bacterial pathogenesis in a more indirect manner. Although the study was not designed to explore the mechanism by which RND efflux pumps are correlated with bacterial virulence, our experimental outcomes provide relevant clues for future research. The bacteria rely on their invasive abilities to cause pathogenicity and disease, where RND efflux pumps, i.e., AcrAB-TolC and MexAB-OprM, are known to disseminate invasion determinants of *E*. *coli* and *P*. *aeruginosa*, respectively [49,50]. Consistent with the previous reports [43], our study demonstrates that Ar5 as an EPI diminished the intracellular bacterial invasion alongside demonstrating the role of overproduction of RND efflux systems in disseminating invasion determinants in *E*. *coli* and *P*. *aeruginosa*. Considering the therapeutic potential of the combination, we determined the *in vivo* efficacy of the Ar5-levofloxacin combination to cure *P*. *aeruginosa* infection in the mice model. We observed notable efficacy of the combination in the murine lung and septicemia infection model. Furthermore, our best EPI was found non-toxic to eukaryotic cells *in vitro;* no morbidity or mortality was observed in acute toxicity studies. Similar observations provided evidence of non-toxicity in histopathological analyses. Moreover, our best compounds exhibit favorable pharmacokinetic properties, evidenced in ADME analyses. Taken together, our combination appears as a silver lining to the tragic situation of MDR Gram-negative bacterial infections and can be considered for further clinical development.

## Materials and methods

### Chemicals, bacterial strains, and growth conditions

The antibiotics, chemicals, and dyes were purchased from Sigma-Aldrich Chemical Co. (St. Louis, MO, United States) unless stated otherwise. The *E*. *coli* wild-type K-12 AG100 (*argE3 thi-1 rpsLxylmtlΔ(gal-uvrB)supE44*) and its derivatives, AcrB over-expressing AG100_tet_ and AcrAB knockout AG100A (*ΔacrAB*::Tn903 Kan^r^) [19,51] were used in the study. AG100_tet_ is derived from AG100 by gradual stepwise exposure to increased concentrations of tetracycline [19,52] and was grown in the presence of 8 μg/mL tetracycline to maintain efflux pump over-expression. AG100A was grown in the presence of 100 μg/mL kanamycin to preserve the transposon. The *P*. *aeruginosa* ATCC BAA-2795 over-expressing MexAB-OprM (Chromachemie, India), and PAO750 (PAO1: *ΔmexAB-oprM*, *ΔmexCD-oprJ*, *ΔmexEF-oprN*, *ΔmexXY*) [53] were also used in the study. The strains *P*. *aeruginosa* ATCC 27853, *P*. *aeruginosa* ATCC 15692 (PAO1), *K*. *Pneumoniae* ATCC BAA-2782, *K*. *Pneumoniae* ATCC 700603, and *E*. *coli* ATCC BAA-2774, were purchased from Himedia, India. All the strains were grown in BBL Cation-adjusted Mueller Hinton Broth (CA-MHB; BD, U.S.) at 37°C under standard conditions. Mueller Hinton Agar (MHA; Himedia, India) plates were used for bacterial viability counting.

### *General chemistry and* general procedure for the synthesis of 4-bromo-1H-2-ethyl carboxylate scaffold derivatives

The complete methodology is provided in the supporting information.

### MIC determination

The complete methodology is provided in the supporting information.

### Checkerboard synergy assay

The synergistic potential of the compounds was determined by broth-microdilution checkerboard synergy assay. The antibiotics were two-fold serially diluted into the wells of 96-well plates (along the abscissa) with different concentrations of compounds along the ordinate. The antibiotic + compound containing wells were dispensed with 100 μL of the bacterial culture (5 × 10^5^ CFU/mL) and incubated for 18 h at 37°C. The MICs of the antibiotics in the presence of compounds were recorded. To determine synergistic effects, the FICIs were calculated according to the formula:

FICI = FICI_A_ + FICI_B_

FICI_A_ = MIC of compound A in combination/MIC of compound A alone

FICI_B_ = MIC of compound B in combination/MIC of compound B alone

The FICI ≤0.5 was regarded as "synergy," 0.5 < FICI ≤ 4.0 was considered an "indifference" effect, and the FICI > 4.0 was considered an "antagonism" [54].

### Hoechst 33342 accumulation assay

*E*. *coli* AG100_tet_ was cultured to exponential phase at 37°C with shaking in CA-MHB. The bacterial cell culture was centrifuged for 15 min at 4,000 ×*g*. After centrifugation, the supernatant was discarded, and the pellet was washed once and resuspended in uptake buffer (110 mM NaCl, 7 mM KCl, 50 mM NH_4_Cl, 0.4 mM Na_2_HPO_4_, and 52 mM Tris base; pH 7.5) [17,55]. The OD_600nm_ of bacterial suspension was adjusted to 0.1 and spiked with Hoechst 33342 (final concentration of 2.5 μM) in the presence or absence of compounds at different concentrations (1/4 × MIC, 1/8 × MIC, or 1/16 × MIC). PAβN was included as a positive control. The increase in fluorescence was measured for 45 min at an excitation wavelength of 355 nm and emission wavelength of 460 nm in a microplate reader (BioTek, U.S.). The RFF was determined by the formula: RFF = (RFF_treated_ at 45 min–RFF_untreated_ at 45 min)/ RFF_untreated_ at 45 min [19].

### Hoechst 33342 efflux inhibition assay

The efflux inhibitory ability of compounds was fluorometrically estimated by following a method described earlier [18], with some modifications. The *E*. *coli* AG100_tet_ was grown to log phase with shaking at 37°C. The culture was centrifuged at 4,000 ×*g* for 10 min, washed twice, and finally diluted to OD_600nm_ of 0.4 in 1X phosphate-buffered saline (PBS; Gibco, Thermo Fisher Scientific, U.S.), pH 7.4. Further, the cells were spiked with Hoechst 33342 (2.5 μM final concentration) and compounds at 1/4 × MIC and incubated at 25°C for 60 min (without glucose) to facilitate maximum accumulation. The cells treated with PAβN (64 μg/mL) served as a positive control. After 60 min of incubation, the bacterial suspensions were centrifuged at 13,000 ×*g* for 15 min and re-suspended in PBS. Further, the aliquots were dispensed into 96-well polystyrene black plates (Corning, U.S.). The fluorescence (excitation 350 nm and emission 460 nm) was recorded for a time period of 45 min (at 9 min intervals) in a microplate reader.

### Measurement of tetracycline accumulation

The measurement of tetracycline accumulation was performed as described earlier [56] with some modifications. The freshly grown *E*. *coli* AG100_tet_ (mid-log phase) was pelleted at 9,000 ×*g* for 5 min and then washed twice in 10 mM 4- (2-hydroxyethyl)-1-piperazineethanesulfonic acid (HEPES) buffer, pH 7.2. Further, the bacterial culture was diluted to 10^8^ CFU/mL in 10 mM HEPES and boosted with tetracycline (1×MIC). After that, compounds (1/4×MIC) were added to tetracycline containing bacterial suspension and loaded into 96-well polystyrene black plates. In the control experiment, DMSO was added instead of compounds. The increase in fluorescence was measured at an emission wavelength of 535 nm for 25 min (5 min intervals) by excitation at 405 nm.

### Molecular docking studies

The complete methodology is provided in the supporting information.

### Cloning, expression, and purification of AcrB

The complete methodology is provided in the supporting information.

### Site-directed mutagenesis, expression, and purification of AcrB

The complete methodology is provided in the supporting information.

### Bio-layer interferometry (BLI)

To study the interaction of AcrB with various compounds, we performed BLI using Forte Octet RED 96 as previously described [17]. AcrB was diluted to 5 μM in 10 mM Sodium acetate buffer pH 5.0 and loaded onto the AR2G sensor tip after its activation by EDC/NHS coupling chemistry. After loading the AR2G sensor tip with AcrB blocking was performed by its treatment with 1 M Ethanolamine. A blank sensor tip was used as a control to subtract the non-specific signal. After immobilization of AcrB onto the sensor tip binding study of AcrB with different ligands (diluted in 20 mM HEPES pH 8.0, 150 mM NaCl and 5% (v/v) DMSO) was performed using different concentrations of ligands (5 μM, 50 μM, 150 μM, 500 μM and, 1000 μM) and monitored for association (300 s) and dissociation (300 s). The sensor tip was regenerated and neutralized after the association-dissociation cycle of a single concentration with 5 mM NaOH. After the experiment, data were analyzed using Data Analysis 8.0 software. Raw data was first subtracted from the reference well-aligned and processed with Savitzky-Golay filtering. After processing, data were fitted using the 1:1 model.

To check the binding of the three mutant constructs of AcrB, 10 μM of the protein was loaded on to pre-hydrated Ni-NTA (ForteBio, USA) sensors as per the manufacturer’s recommendations. Another sensor was also loaded with the same amount of protein to use as a reference. 500 μM of Ar1, Ar5, Ar11, and Ar18 compounds were used as ligands, diluted in 20 mM HEPES (pH 8.0), 150 mM NaCl, 0.02% DDM and 10% DMSO. 1% BSA was used to avoid non-specific interactions. After a stable baseline (300s) was obtained, association (150s) and dissociation (300s) were monitored. The protein was regenerated using 5 mM NaOH and the sensors were recharged using 10 mM Nickel chloride. ForteBio data analysis software 8.0 was used to analyze the data after the experiment.

### Time-kill kinetics

The freshly grown *E*. *coli* ATCC BAA-2774 was diluted to 5 × 10^5^ CFU/mL in CA-MHB and treated with either Ar5 (1/4 × MIC) or levofloxacin (1 × MIC) alone or their combination. The untreated bacterial suspension served as a control. The different treatment sets were incubated at 37°C for 24 h with shaking. A 100 μL of culture sample was withdrawn at different time points, appropriately diluted, plated on MHA, and plates were incubated for 24 h at 37°C. After 24 h, the colonies were counted, and based on colony number, CFU/mL was calculated. Each set’s log10 CFU/mL was plotted against different time points.

### Persister-killing assay

The effect of the antibiotic + EPI combination on antibiotic-induced persister cells was determined by following a method described earlier [57], with slight modifications. Initially, the antibiotic concentration to be used was optimized by performing kill-kinetics studies at different antibiotic concentrations. The concentration that no longer inhibits or allows bacterial growth such that a stable plateau phase is reached was used in the persister assay. Briefly, the bacterial cultures *E*. *coli* AG100 and *P*. *aeruginosa* ATCC 27853 were grown in Luria Bertani (LB) broth, Miller (Himedia, India) for 48 h. After 48 h, stationary phase *E*. *coli* and *P*. *aeruginosa* were treated with carbenicillin at 16 × MIC (128 μg/mL) and 8 × MIC (256 μg/mL), respectively, for 18 h. Following treatment, the bacterial culture was pelleted and washed twice in 1X PBS to remove antibiotic traces. Isolated persisters were then resuspended in 1X PBS, diluting up to five times the original volume. The diluted bacterial suspensions of *E*. *coli* and *P*. *aeruginosa* were again treated with carbenicillin alone as well as in the presence of Ar5 (16 μg/mL) and incubated at 37°C. The untreated culture was included as a control. The samples were withdrawn at different time intervals, spotted onto LB agar plates, plates were incubated for 24 h at 37°C, and viable bacterial counts were assessed.

### Determination of the post-antibiotic effect by turbidimetry

The *E*. *coli* ATCC BAA-2774 was grown to the mid-exponential phase (OD_600nm_ ≈ 0.25) in CA-MHB. The bacterial culture was treated with levofloxacin (32 μg/mL) alone, combined with Ar1, Ar5, Ar11, or Ar18 at sub-inhibitory concentrations (16 μg/mL) and incubated for 2 h at 37°C with shaking. Further, the bacterial cultures were diluted 50 times to eliminate the effect of drug carryover, and the diluted culture (250 μL volume in triplicates) was dispensed into a 96-well plate. The untreated bacterial suspension served as a control. The absorbance (OD_600nm_) was measured for 12 h in a microplate reader. PAE was calculated by a formula: PAE = T_50_ –C_50_; T_50_ = time (hours) needed for the drug-treated culture and C_50_ = time (hours) needed for the untreated cultures, to attain an OD_600nm_ = 50% of the final absorbance attained by an untreated culture. The OD_600nm_ of the control set and treated suspension were adjusted equal prior to growth resurgence to lessen the difference in the inoculums [58].

### MPC determination

The effect of compounds on reducing the emergence of spontaneous resistant mutants was determined by a method described earlier [59,60]. The 100 μL (~10^11^ CFU) volume from concentrated log-phase culture of *K*. *pneumoniae* ATCC 700603 was spread on MHA plates supplemented with different concentrations of levofloxacin alone and combined with the sub-inhibitory concentration (1/4 × MIC) of compounds (Ar1, Ar5, Ar11, Ar18). The plates were incubated at 37°C for 48 h, and colonies were counted. The concentration at which no bacterial colony emerged was regarded as the antibiotic’s MPC. The mutation frequency was calculated by the formula:

Mutation frequency = number of survivors/ CFU plated

### ROS measurement

The ROS levels in *E*. *coli* and *P*. *aeruginosa* were measured according to a protocol described elsewhere [27] with some modifications. The bacterial cultures of *E*. *coli* AG100_tet_ and *P*. *aeruginosa* ATCC BAA-2795 were grown in CA-MHB, washed, and resuspended in 1X PBS to obtain an OD_600nm_ of 0.5. The bacterial suspension was incubated with 10 μM 2’-7’dichlorofluorescin diacetate (DCFH-DA) for 1 h at 37°C. After incubation, the bacterial cells were washed thrice with 1X PBS, treated with Ar1, Ar5, Ar11, and Ar18 at sub-inhibitory concentrations (16 μg/mL) alone or in combination with levofloxacin and piperacillin at 1 × MIC. The treated bacterial suspension was dispensed into a 96-well black flat-bottom plate. The antioxidant N-acetyl-L-cysteine (NAC; 6 mM) was used as a control to neutralize ROS production. After incubation for another 2 h, the fluorescence was immediately measured at excitation and emission wavelengths of 488 nm and 525 nm, respectively.

### Biofilm eradication assay

The complete methodology is provided in the supporting information.

### Outer membrane permeabilization assay

The complete methodology is provided in the supporting information.

### Membrane depolarization assay

The complete methodology is provided in the supporting information.

### ATP determination assay

The complete methodology is provided in the supporting information.

### Membrane fluidity assay

The complete methodology is provided in the supporting information.

### Mammalian calcium channel blocking assay

The effect of compounds (Ar1-Ar24) on human Ca^2+^ channels was evaluated using Fluo-4 Direct calcium channel assay kit (Life Technologies, Carlsbad, CA), according to the manufacturer’s instructions. As described earlier [31,39], the 2X Fluo-4 Direct calcium reagent loading solution containing probenecid (5 mM) was added in equal volume to the 96-well plate containing 5 × 10^4^ HEK-293 (AddexBio, San Diego, CA) cells/well. The plates were allowed to equilibrate for 1 h at 37°C with 5% CO_2,_ and fluorescence (pre-stimulation) was immediately measured in a microplate reader. The cells were then treated with Ar1-Ar24 (16 μg/mL), Ca^2+^ channel inhibitor verapamil (32 μg/mL), and DMSO (mock) at 30 s. Then at 60 s, carbachol (50 μg/mL; calcium channel stimulator) was added, and the fluorescence (post-stimulation) was recorded for an additional 120 s.

### Cytotoxicity assay on H-PBMCs

The cytotoxicity assay was performed on Human Peripheral Blood Mononuclear cells (H-PBMC, Himedia, India) according to the manufacturer’s instructions. The H-PBMC (40,000 cells/well in 100 μL volume) suspension in RPMI-1640 media (containing 10% fetal bovine serum and 50 U/mL antibiotic-antimycotic solution) (Himedia^,^ India) was dispensed into 96-well polystyrene tissue culture-treated flat-bottom plates (Corning, U.S.). The plates were incubated for 48 h in a CO_2_ (5%) incubator at 37°C. After 48 h, the compounds (Ar1-Ar24) were added to the plates in a fresh medium and again incubated for 24 h in a CO_2_ (5%) incubator at 37°C. Next, MTT (1 mg/mL) was added to the wells, followed by incubation for 4–5 h. The formazan crystals were solubilized using a solubilizing solution (40% (v/v) dimethyl formamide in 2% (v/v) glacial acetic acid added with 16% sodium dodecyl sulfate), and the absorbance (OD_570nm_) was measured.

### Hemolysis assay

The hemolytic assay was performed on rabbit red blood cells (RBCs) as described previously [17]. Briefly, fresh rabbit blood was collected in a BD Vacutainer tube, centrifuged at 1000 ×*g*, and washed RBCs thrice with 1X PBS. A suspension of 4% RBCs prepared in 1X PBS was dispensed in 96-well flat-bottom plates and incubated with Ar1-Ar24 (512 μg/mL– 64 μg/mL) for 1 h. Triton X-100 (0.1%) was included as a positive control, and cells without treatment served as a negative control. After incubation, the plates were centrifuged (1000 ×*g*) for 5 min at 4°C. The 100 μL supernatant was transferred to another 96-well flat-bottom plate, and absorbance (OD_570nm_) of released hemoglobin was measured.

### Growth kinetics determination and Motility assays

The complete methodology is provided in the supporting information.

### Pyocyanin assay

The effect of EPIs on pyocyanin levels of *P*. *aeruginosa* PAO1 and PAO750 was quantified by a method described earlier [61]. *P*. *aeruginosa* PAO1 and PAO750 were grown in CA-MHB supplemented with Ar1, Ar5, Ar11, and Ar18 (16 μg/mL) for 48 h at 37°C. Further, 5-mL cell-free supernatants were extracted into 3 mL chloroform and re-extracted by mixing with 0.2 N HCl to extract acidic red pyocyanin. The untreated bacterial culture served as a control. Pyocyanin levels were quantified by measuring the solution’s absorbance (OD_520nm_).

### Pyoverdine assay

According to a method specified elsewhere [62], *P*. *aeruginosa* PAO1 and PAO750 were grown overnight in a casamino acid medium (0.5% casamino acids, 0.1 mM MgSO_4_, 7 mM potassium phosphate buffer, pH 7.0) in the presence of Ar1, Ar5, Ar11, Ar18 (16 μg/mL). The bacterial cultures were centrifuged, and the supernatant was collected in separate tubes. Further, 5 μL of supernatant was mixed with 995 μL of 10 mM Tris HCl (pH 6.8). The pyoverdine levels were quantified in a 96-well black plate (flat-bottom) by measuring fluorescence at an emission wavelength of 700 nm by excitation at 405 nm.

### Elastase assay

*P*. *aeruginosa* PAO1 and PAO750 were grown to OD_600nm_ of 0.5 in tryptic soy broth (0.25%) supplemented with peptone (5%) [63]. The bacterial culture was diluted 10 times in fresh media and incubated with compounds (Ar1, Ar5, Ar11, Ar18) at a sub-inhibitory concentration (16 μg/mL) for 18 h. After incubation, the culture was centrifuged, and the supernatant (100 μL) was incubated with 900 μL Tris buffer (100 mM Tris, 1 mM CaCl_2_, pH 7.5) supplemented with 20 mg Elastin-Congo red for 3 h. After 3 h, the solution was mixed with an equal volume of sodium ethylenediaminetetraacetic acid (Na_2_-EDTA) buffer and incubated on ice (15 min) to stop the action of the enzyme. After incubation, the reaction mixture was pelleted at 13,000 ×*g* for 15 min, the supernatant was dispensed to a 96-well flat-bottom plate (in triplicates), and the OD_495nm_ was measured [63].

### Protease assay

The bacterial culture of *P*. *aeruginosa* PAO1 and PAO750 were grown overnight and diluted 1:200 in fresh CA-MHB containing sub-inhibitory concentrations (16 μg/mL) of compounds (Ar1, Ar5, Ar11, Ar18) and incubated for 18 h at 37°C, with shaking (200 rpm). Post-incubation, the culture was centrifuged, and cell-free supernatants were incubated with an equal volume of 1.25% w/v skim milk [64] (Himedia, India) for 30 min, with shaking at 37°C. The untreated culture was included as a control. Further, the reaction mixture was dispensed in triplicates into a 96-well plate (flat-bottom), and absorbance (OD_600nm_) was measured.

### Rhamnolipids assay

The effect of EPIs on rhamnolipids was assessed by following a method described earlier [65], with some modifications. The OD_600nm_ of *P*. *aeruginosa* PAO1 and PAO750 was adjusted to 0.5, diluted 10 times in fresh PPGAS (0.02 M NH_4_Cl_2_, 0.02 M KCl, 0.12 M Tris-HCl, 1.6 mM MgSO_4_, 0.5% glucose, 1% peptone, pH 7.2) [66] medium, and incubated at 37°C for 24 h in the presence of compounds (Ar1, Ar5, Ar11, and Ar18) at 16 μg/mL. After 24 h, the culture was centrifuged at 13,000 ×*g* for 15 min. Then, 500 μL of supernatant was added to 1 mL of diethyl ether, shaken, and let it settle down. Next, 900 μL of the upper organic layer was collected and subject to dry at 80° C for 15 min. Afterward, 900 μL of orcinol-reagent (0.19% orcinol in 53% sulphuric acid) was added, incubated at 80°C for 30 min, cooled at room temperature (for 15 min), and OD_421nm_ was measured. The amount of rhamnolipids in samples was generated using a standard curve of OD_421nm_ versus concentration of rhamnose (Himedia, India).

### RNA isolation, RNA sequencing and qRT-PCR

The complete methodology is provided in the supporting information.

### *C*. *elegans* survival assay

*C*. *elegans* survival assay was performed according to a protocol described earlier [16], with some modifications. Briefly, *C*. *elegans* wild-type N2 Bristol worms were propagated on the *E*. *coli* OP50 spread on NGM plates. The eggs were harvested from gravid adults using bleaching (30 mL 5% bleach, 15 mL 5M KOH, 55 mL dH_2_O); harvested eggs were placed on *E*. *coli* OP50 lawns and incubated for 48 h at 20°C. After 48 h, the worms reached the L4 stage and were transferred to lawns of *P*. *aeruginosa* PAO1 and PAO750 grown (for 18 h) in the presence or absence of Ar5 (16 μg/mL). The control (without treatment) plates were supplemented with an equivalent volume of DMSO (vehicle). For survival assay, 10 L4 worms were placed in triplicate plates for each experimental condition. The plates were scored for live/dead worms at different time intervals for 24 h. The distinction between live versus dead was made based on the movement of the worms in response to physical stress delivered via gentle poking. Data analysis was done by using Kaplan-Meier survival plots.

### Determination of bile salts potentiation

The complete methodology is provided in the supporting information.

### Macrophage invasion assay

For assay [55], the macrophage cells RAW264.7 (AddexBio, San Diego, CA) were cultured in DMEM (containing 10% fetal bovine serum and 50 U/mL antibiotic-antimycotic solution) (Himedia^,^ India) at 37°C in a CO_2_ incubator (5% CO_2_). The 500 μL suspension (~10^5^ cells/well) was dispensed into 24-well plates (Corning, U.S.) and incubated for 24 h to allow adherence. Post-incubation, the cells were infected with 5 μL of bacterial culture (OD_600nm_ = 0.3) of *E*. *coli* and *P*. *aeruginosa* in the presence or absence of Ar5 (16 μg/mL) in a fresh medium. The plates were incubated for 1 h in a CO_2_ incubator. After 1 h of incubation, the media was discarded, wells were washed twice with 1X PBS, then treated (for 30 min) with 50 μg/mL gentamicin to remove extracellular bacteria, and yet again washed with 1X PBS to remove traces of the antibiotic. The intracellular bacteria were recovered by lysis of the host cell with a brief exposure to a mild reagent (0.1% saponin). Further, the viability of intracellular bacteria was assessed by spreading on MHA plates.

### Ethics statement for animal usage

Animal experiments were conducted according to the guidelines set by the Committee for the Purpose of Control and Supervision of Experiments on Animals (CPCSEA) using procedures approved by the Institutional Animal Ethics Committee (IAEC) at iCARE (Reg. No. 55/GO/Re/Rc/Bi/Bt/S/99/CPCSEA) facility of CSIR-Institute of Microbial Technology, India under protocol IAEC/20/04. Before the experiment, mice were randomly relocated from a housing cage to treatment or control cages. Blinding was considered unnecessary for mouse infection studies. During histopathology analyses, the observer was unaware of the control and treatment groups.

### *In vivo* murine lung infection model

Female BALB/c mice (*n* = 6 mice/group) were rendered neutropenic by intraperitoneal administration of cyclophosphamide; the first dose of 150 mg/kg four days before infection and second dose of 100 mg/kg one day prior to infection. An inoculum of (OD_600nm_ = 2.0) of *P*. *aeruginosa* ATCC BAA-2795 was re-suspended in sterile 1X PBS, and 10 μL of the suspension was administered intranasally. One group (pre-treatment) was deeply anesthetized and euthanized by cervical dislocation at 4 h post-infection. The lungs were aseptically removed and homogenized into 1X PBS, appropriately diluted, and finally spread on MHA plates for CFU counts. The remaining groups of mice were treated by subcutaneous injections of either levofloxacin (20 mg/kg) or Ar5 (5 mg/kg) alone or in combination. At 20 h after treatment, the remaining mice were euthanized under deep anesthesia, lungs were dissected out, and CFUs in the lungs were assessed.

### Murine peritonitis survival model

The survival study was performed by following a method described earlier [67], with some modifications. Female BALB/c mice (*n* = 10 mice/group) were infected with 100 μL (OD_600nm_ = 1.0) of *P*. *aeruginosa* ATCC BAA-2795 bacterial suspension (prepared in 1X PBS containing 5% porcine mucin) via intraperitoneal injection, which achieves 90% mortality within 24 h of infection. Mice were injected subcutaneously with levofloxacin alone (20 mg/kg), Ar5 alone (5 mg/kg), or levofloxacin combined with Ar5. The repetitive dosing was given at 1 h, 4 h, and 7 h post-infection. The survival of mice was monitored every 12 h until 96 h post-infection.

### Acute toxicity, histopathology, and blood biochemical studies

Acute toxicity was performed by following a proposed (new) method described elsewhere [68]. The study was performed in three stages, and the procession to the next stage depended on the previous stage’s results. The first stage involved 5 groups of mice (one animal in each group), the second stage involved 4 groups of mice, third stage involved 2 groups of mice. Each group had mice injected subcutaneously with increasing dosage of Ar5 (single dose) *viz*; stage 1 (vehicle control, 10 mg/kg, 25 mg/kg, 50 mg/kg, and 100 mg/kg), stage 2 (vehicle control, 250 mg/kg, 500 mg/kg, and 750 mg/kg), stage 3 (vehicle control and 1000 mg/kg). After dosing, the mice of all stages were observed at 1 h intervals for an initial 5 h and after 24 h. A confirmatory test (involving 2 mice per group) for the mice injected with the highest dose (1000 mg/kg) and dosed lower than the highest (750 mg/kg) was carried out to validate the results.

Before euthanization, the mice’s body weight and blood (collected through the tail vein) sugar were assessed. Then, the mice were anesthetized with ketamine (90 mg/kg) + xylazine (10 mg/kg) by intraperitoneal administration, followed by blood collection through cardiac puncture and retro-orbital puncture. The six major organs (brain, heart, lungs, liver, spleen, and kidney) were dissected out and immediately preserved in 10% buffered formalin and submitted for histological staining. The serum was prepared from the collected blood for evaluation of various biochemical parameters namely ALT/SGPT, and AST/SGOT, ALP, total bilirubin, creatinine, triglycerides, cholesterol, albumin, and total protein.

### *In silico* ADME analysis

The complete methodology is provided in the supporting information.

### Statistical significance

The values reported were shown as mean ± standard deviation. The sample size for each statistical analysis was 3, and the statistical significance and comparisons among groups were analyzed by multiple paired *t*-test. A *p-value <0.05 was regarded as statistically significant and **p< 0.01 and ***p < 0.001 as highly significant. Statistical significance in experimental data was determined using GraphPad Prism 9.3.1 software.

## Supporting information

S1 Table Antibiotics potentiation assays in the presence of most active compounds (Ar1, Ar5, Ar11, Ar18) against XDR strains of *K*. *pneumoniae* ATCC BAA-2782, *E*. *coli* ATCC BAA-2774, *P*. *aeruginosa* ATCC BAA-2795. (DOCX)

S2 Table Antibiotic potentiation assays in the presence of most active compounds against XDR *P*. *aeruginosa ATCC* BAA-2795. (DOCX)

S3 Table Effect of efflux pump deletion on antibiotic MICs in the presence of EPIs against hypersusceptible *P*. *aeruginosa* PAO750. (DOCX)

S4 Table Minimum inhibitory concentrations (MICs) of non-MexB substrate antibiotics on *P*. *aeruginosa* ATCC BAA-2795 in the presence of EPIs. (DOCX)

S5 Table Relative final fluorescence (RFF) values representing Hoechst 33342 accumulation for the *E*.*coli* AG100_tet_ strain in the presence of the efflux inhibitors. (DOCX)

S6 Table Binding energy (kcal/mol) of lead compounds (Ar1, Ar5, Ar11, Ar18) and PAβN to AcrB and MexB efflux pump protein of *E*. *coli* and *P*. *aeruginosa*. (DOCX)

S7 Table Binding energy (kcal/mol) of lead compounds (Ar1, Ar5, Ar11, Ar18) and MBX3135 to AcrB and MexB mutants. (DOCX)

S8 Table Cytotoxicity profile of synthesized compounds (Ar1-Ar24) on human peripheral blood mononuclear cells (H-PBMCs) as determined by using the MTT assay. (DOCX)

S9 Table Hemolytic activity of the synthesized compounds (Ar1-Ar24) on rabbit blood erythrocytes. (DOCX)

S10 Table Differential gene expression of the virulence factor genes of *P*. *aeruginosa* PAO1 in response to the treatment of Ar5 and PAβN (positive control) compared to the untreated control. (DOCX)

S11 Table Acute toxicity of compound Ar5 in BALB/c mice. (DOCX)

S12 Table *In silico* ADME analyses. (DOCX)

S1 Fig Structure-activity relationship between synthesized derivatives. (TIF)

S2 Fig Molecular docking of Ar ligands with MexB. Structural representation of Ar series ligands (red spheres) docked in MexB (green cartoon) (A) Ar1 (B) Ar5 (C) Ar11 (D) Ar18 docked in the active site cleft of MexB. 2D ligand interaction diagram portraying various interactions involved in binding of (E) Ar1 (F) Ar5 (G) Ar11 (H) Ar18 to MexB.
(TIF)

S3 Fig Molecular docking of PAβN with AcrB and MexB. PAβN (red spheres) docked in the active site cleft of (A) AcrB (green cartoon) and (B) MexB (green cartoon). 2D ligand interaction diagram portraying various interactions involved in binding of PAβN to (C) AcrB and (D) MexB.
(TIF)

S4 Fig Representation of Ar compounds docked with AcrB and AcrB mutants. For clarity, AcrB is shown in cartoon representation and ligands are shown in stick representation. The Ar compounds are coloured in pink (docked with AcrB), light green (docked with AcrB^F178A^), yellow (docked with AcrB^F628A^), and cyan (docked with AcrB^F615A, F617A, R620A^).
(TIF)

S5 Fig (A) Binding of Ar1, Ar5, Ar11, and Ar18 with AcrB^F615A, F617A, R620A^ protein (B) Binding of A1, Ar5, Ar11 and Ar18 with AcrB^F178A^ protein.
(TIF)

S6 Fig **Persister-killing assay** (A) Biphasic killing curves represent the persister formation frequency of *P*. *aeruginosa* ATCC 27853 under ciprofloxacin treatment at different concentrations (2, 4, and 8 × MIC). (B) Biphasic killing curves represent the persister formation frequency of *P*. *aeruginosa* ATCC 27853 under ciprofloxacin (8 × MIC) treatment in the absence or presence of Ar5 at sub-inhibitory concentrations (8 μg/mL and 16 μg/mL).
(TIF)

S7 Fig Motility assays. *P*. *aeruginosa* PAO1 (A) swarming motility (B) twitching motility in the absence (control) and presence of EPIs (Ar1, Ar5, Ar11, and Ar18) at sub-inhibitory concentrations (1/4 × MIC; 16 μg/mL). *P*. *aeruginosa* PAO750 (C) swimming motility (D) swarming motility (E) twitching motility in the absence and presence of EPIs (Ar1, Ar5, Ar11, and Ar18) at sub-inhibitory concentrations (1/4 × MIC; 16 μg/mL). For visualization of twitching, the attached cells were stained with crystal violet (1% w/v) for 5 min after carefully removing the agar, followed by washing to remove excess stain.
(TIF)

S8 Fig Effect of EPIs (Ar1, Ar5, Ar11, and Ar18) on the production of extracellular virulence factors; pyocyanin (A), pyoverdine (B), elastase (C), protease (D), and rhamnolipids (E) levels in the culture supernatants of *P*. *aeruginosa* PAO750 in the presence of sub-inhibitory concentrations of EPIs (16 μg/mL). (F) Effect of Ar5 on *P*. *aeruginosa* PAO750 virulence toward *C*. *elegans*.
(TIF)

S9 Fig **Effect of (A) Ar5 and (B) PAβN on virulence genes expression.** The interactive heat map representing a differential expression of *P*. *aeruginosa* PAO1 virulence genes on a gradient scale in response to treatment with Ar5 (16 μg/mL) and PAβN (16 μg/mL) for 18 h.
(TIF)

S10 Fig Quantitation of genes of three quorum sensing systems of *P*. *aeruginosa* PAO1 by quantitative real-time polymerase chain reaction (qRT-PCR) after treatment with our lead efflux pump inhibitor Ar5. Gene transcripts levels were normalized against the *S*. *aureus* 16s rRNA gene. The relative fold expression levels were calculated using the 2-ΔΔCT method. The average of triplicates ± SD is shown.
(TIF)

S11 Fig Viability of *P*. *aeruginosa* PAO1 (expressing MexB) in the presence of Ar5 (16 μg/mL) and PAβN (50 μg/mL, 25 μg/mL) with or without 0.15% bile salts. The average of triplicates ± SD is shown. Results were considered significant when *p<0.05 and highly significant when **p<0.01 and ***p<0.001.
(TIF)

S1 Supporting Information S1 Method. General chemistry; S2 Method. MIC determination; S3 Method. Molecular docking studies; S4 Method. Cloning, expression, and purification of AcrB; S5 Method. Site-directed mutagenesis, expression, and purification of AcrB; S6 Method. Persister assay for *P*. *aeruginosa*; S7 Method. Biofilm eradication assa; S8 Method. Outer membrane permeabilization assay; S9 Method. Membrane depolarization assay; S10 Method. ATP determination assay; S11 Method. Membrane fluidity assay; S12 Method. Growth kinetics determination; S13 Method. Motility assays; S14 Method. RNA isolation, RNA sequencing, and qRT-PCR; S15 Method. Determination of bile salts potentiation; S16 Method. *In silico* absorption, distribution, metabolism, and excretion (ADME) analysis.
(PDF)

S1 Data Contains numerical data used to make figures and tables. (XLSX)
