# Supplementary material for: Pyrrole-based inhibitors of RND-type efflux pumps reverse antibiotic resistance and display anti-virulence potential
Source: PLoS Pathog. 2024 Apr 9;20(4):e1012121. doi: 10.1371/journal.ppat.1012121 (PMC11003683; doi:10.1371/journal.ppat.1012121)
Supplement: S1 Table — (DOCX) [file ppat.1012121.s001.docx]

**S1 Table**. Antibiotics potentiation assays in the presence of most active compounds (Ar1, Ar5, Ar11, Ar18) at 16 µg/mL against XDR strains of *K. pneumoniae* ATCC BAA-2782, *E. coli* ATCC BAA-2774, *P. aeruginosa* ATCC BAA-2795. PAβN (64 µg/mL for *E. coli*, 32 µg/mL for *K. penumoniae*, and *P. aeruginosa*) and NMP (64 µg/mL for *E. coli*, 16 µg/mL for *K. penumoniae*, and *P. aeruginosa*) were used as control. The experiment was performed in three biological replicates and two technical replicates.

| Antibiotics | MIC (μg/mL) | Fold reduction in the MICs in the presence of Efflux Pump Inhibitors (EPIs) | | | | | |
| --- | --- | --- | --- | --- | --- | --- | --- |
|  |  | Ar 1 | Ar 5 | Ar 11 | Ar 18 | PAβN | NMP |
| *Klebsiella pneumoniae* ATCC BAA-2782 | | | | | | | |
| Ciprofloxacin | 128 | 16 | 32 | 16 | 16 | - | - |
| Levofloxacin | 32 | 8 | 32 | 16 | 8 | 2 | 8 |
| Tetracycline | 4 | 16 | 32 | 8 | 16 | - | 16 |
| Tigecycline | 64 | 32 | 16 | 16 | 8 | - | - |
| Erythromycin | 128 | 8 | 16 | 8 | 16 | 2 | 64 |
| Piperacillin | 1024 | 16 | 64 | 32 | 16 | - | - |
| Chloramphenicol | 16 | 8 | 8 | 4 | 4 | - | - |
| *Escherichia coli* ATCC BAA-2774 | | | | | | | |
| Ciprofloxacin | 256 | 32 | 64 | 32 | 16 | 8 | 4 |
| Levofloxacin | 32 | 16 | 32 | 16 | 8 | 8 | 4 |
| Tetracycline | 256 | 8 | 8 | 4 | 8 | 4 | 2 |
| Tigecycline | 128 | 4 | 8 | 4 | 2 | 2 | - |
| Erythromycin | 512 | 16 | 32 | 16 | 16 | 8 | 8 |
| Piperacillin | 512 | 8 | 32 | 32 | 16 | 8 | 4 |
| Chloramphenicol | 32 | 32 | 32 | 16 | 8 | 16 | 8 |
| *Pseudomonas aeruginosa* ATCC BAA-2795 | | | | | | | |
| Ciprofloxacin | 256 | 16 | 32 | 16 | 8 | 8 | 4 |
| Levofloxacin | 256 | 4 | 16 | 8 | 4 | 4 | 2 |
| Tetracycline | 32 | 16 | 32 | 16 | 16 | 4 | 4 |
| Tigecycline | 256 | 8 | 16 | 4 | 8 | 4 | 2 |
| Erythromycin | 512 | 16 | 32 | 16 | 8 | 4 | 8 |
| Piperacillin | 256 | 32 | 64 | 16 | 8 | 8 | 4 |
| Chloramphenicol | 256 | 16 | 16 | 8 | 4 | 4 | 4 |
