# Supplementary material for: Pyrrole-based inhibitors of RND-type efflux pumps reverse antibiotic resistance and display anti-virulence potential
Source: PLoS Pathog. 2024 Apr 9;20(4):e1012121. doi: 10.1371/journal.ppat.1012121 (PMC11003683; doi:10.1371/journal.ppat.1012121)
Supplement: S2 Table — (DOCX) [file ppat.1012121.s002.docx]

**S2 Table.** Antibiotic potentiation assays in the presence of most active compounds (Ar1, Ar5, Ar11, Ar18 at 16 µg/mL) against XDR *P. aeruginosa ATCC* BAA-2795. PAβN (32 µg/mL) and NMP (16 µg/mL) were used as controls. The mode values of three biological repeats are represented.

| Antibiotics | MIC (μg/mL) | Conc. of EPIs (μg/mL) | Fold reduction in the MICs in the presence of Efflux Pump Inhibitors (EPIs) | | | | | |
| --- | --- | --- | --- | --- | --- | --- | --- | --- |
|  |  |  | Ar 1 | Ar 5 | Ar 11 | Ar 18 | PAβN | NMP |
| Ciprofloxacin | 256 | 16 | 16 | 32 | 16 | 8 | 8 | 4 |
|  |  | 8 | 8 | 16 | 4 | 2 | 4 | 2 |
|  |  | 4 | 2 | 8 | 2 | - | - | - |
| Levofloxacin | 256 | 16 | 4 | 16 | 8 | 4 | 4 | 2 |
|  |  | 8 | 2 | 8 | 4 | 2 | 2 | - |
|  |  | 4 | 2 | 4 | 2 | - | 2 | - |
| Tetracycline | 32 | 16 | 16 | 32 | 16 | 16 | 4 | 4 |
|  |  | 8 | 4 | 16 | 8 | 4 | 2 | 2 |
|  |  | 4 | - | 8 | 4 | 2 | 2 | - |
| Tigecycline | 256 | 16 | 8 | 16 | 4 | 8 | 4 | 2 |
|  |  | 8 | 4 | 8 | - | 4 | 2 | - |
|  |  | 4 | 2 | 4 | - | 2 | 2 | - |
| Erythromycin | 512 | 16 | 16 | 32 | 16 | 8 | 4 | 8 |
|  |  | 8 | 8 | 16 | 4 | 4 | 2 | 4 |
|  |  | 4 | 4 | 4 | 2 | - | - | 2 |
| Piperacillin | 256 | 16 | 32 | 64 | 16 | 8 | 8 | 4 |
|  |  | 8 | 16 | 32 | 4 | 4 | 2 | 2 |
|  |  | 4 | 2 | 8 | - | 2 | - | - |
| Chloramphenicol | 256 | 16 | 16 | 16 | 8 | 4 | 4 | 4 |
|  |  | 8 | 4 | 8 | 4 | 2 | 2 | - |
|  |  | 4 | 2 | 4 | 2 | 2 | - | - |
