# Supplementary material for: Pyrrole-based inhibitors of RND-type efflux pumps reverse antibiotic resistance and display anti-virulence potential
Source: PLoS Pathog. 2024 Apr 9;20(4):e1012121. doi: 10.1371/journal.ppat.1012121 (PMC11003683; doi:10.1371/journal.ppat.1012121)
Supplement: S3 Table — (DOCX) [file ppat.1012121.s003.docx]

**S3 Table.** Effect of efflux pump deletion on antibiotic MICs in the presence of EPIs (Ar1, Ar5, Ar11, Ar18 at 16 μg/mL) against hypersusceptible *P. aeruginosa* PAO750. PAβN (4 μg/mL) and NMP (8 μg/mL) were included for comparison. The experiment was performed in three biological replicates and two technical replicates.

| Antibiotics | MIC (μg/mL) | Fold reduction in the MICs in the presence of Efflux Pump Inhibitors (EPIs) | | | | | |
| --- | --- | --- | --- | --- | --- | --- | --- |
|  |  | Ar 1 | Ar 5 | Ar 11 | Ar 18 | PAβN | NMP |
| Ciprofloxacin | 0.0078 | - | - | - | 2 | - | - |
| Levofloxacin | 0.03125 | - | - | - | - | 2 | - |
| Tetracycline | 0.125 | - | - | - | - | - | - |
| Tigecycline | 0.5 | - | - | 2 | - | - | - |
| Erythromycin | 8 | - | - | - | - | - | - |
| Piperacillin | 0.5 | - | - | - | - | - | - |
| Chloramphenicol | 1 | - | - | - | - | - | - |
