# Supplementary material for: Pyrrole-based inhibitors of RND-type efflux pumps reverse antibiotic resistance and display anti-virulence potential
Source: PLoS Pathog. 2024 Apr 9;20(4):e1012121. doi: 10.1371/journal.ppat.1012121 (PMC11003683; doi:10.1371/journal.ppat.1012121)
Supplement: S4 Table — (DOCX) [file ppat.1012121.s004.docx]

**S4 Table.** Minimum inhibitory concentrations (MICs) of non-MexB substrate antibiotics on *P. aeruginosa* ATCC BAA-2795 in the presence of EPIs. The experiment was performed in three biological replicates.

| Compounds | Concentration (μg/mL) | MICs (μg/mL) | | | | | |
| --- | --- | --- | --- | --- | --- | --- | --- |
|  |  | Tobramycin | | Gentamicin | | Amikacin | |
|  |  | Without EPI | With EPI | Without EPI | With EPI | Without EPI | With EPI |
| Ar1 | 16 | 64 | 64 | 64 | 64 | 256 | 256 |
| Ar5 | 16 | 64 | 64 | 64 | 64 | 256 | 256 |
| Ar11 | 16 | 64 | 64 | 64 | 64 | 256 | 256 |
| Ar18 | 16 | 64 | 64 | 64 | 64 | 256 | 256 |
