# Supplementary material for: Pyrrole-based inhibitors of RND-type efflux pumps reverse antibiotic resistance and display anti-virulence potential
Source: PLoS Pathog. 2024 Apr 9;20(4):e1012121. doi: 10.1371/journal.ppat.1012121 (PMC11003683; doi:10.1371/journal.ppat.1012121)
Supplement: S5 Table — (DOCX) [file ppat.1012121.s005.docx]

**S5 Table.** Relative final fluorescence (RFF) values representing Hoechst 33342 accumulation for the *E. coli* AG100_tet_ strain in the presence of the efflux inhibitors (Ar1 - Ar24) at 1/4 × MIC (16 µg/mL), 1/8 × MIC (8 µg/mL), 1/16 × MIC (4 µg/mL) and PAβN (64 µg/mL, 32 µg/mL, 16 µg/mL). The experiment was performed in two biological replicates; average ± SD values from an independent experiment are represented.

| **Compounds** | **Relative Final Fluorescence (RFF ± SD)** | | |
| --- | --- | --- | --- |
|  | **1/4 × MIC** | **1/8 × MIC** | **1/16 × MIC** |
| **Ar1** | 7.42 ± 0.3 | 4.78 ± 0.07 | 3.53 ± 0.05 |
| **Ar2** | 2.25 ± 0.01 | 2.32 ± 0.05 | 1.39 ± 0.009 |
| **Ar3** | 2.24 ± 0.25 | 1.42 ± 0.06 | 0.09 ± 0.06 |
| **Ar4** | 2.82 ± 0.15 | 1.06 ± 0.06 | 1.61 ± 0.15 |
| **Ar5** | 7.68 ± 0.08 | 4.78 ± 0.28 | 2.31 ± 0.02 |
| **Ar6** | 1.30 ± 0.05 | 0.08 ± 0.01 | 0.04 ± 0.03 |
| **Ar7** | 2.22 ± 0.16 | 1.35 ± 0.06 | 1.35 ± 0.08 |
| **Ar8** | 2.29 ± 0.02 | 1.38 ± 0.07 | 1.33 ± 0.09 |
| **Ar9** | 1.18 ± 0.07 | 0.07 ± 0.01 | 0.05 ± 0.04 |
| **Ar10** | 4.88 ± 0.07 | 3.63 ± 0.11 | 2.30 ± 0.007 |
| **Ar11** | 7.65 ± 0.39 | 4.71 ± 0.04 | 3.48 ± 0.13 |
| **Ar12** | 3.42 ± 0.09 | 2.33 ± 0.006 | 1.36 ± 0.02 |
| **Ar13** | 4.68 ± 0.11 | 3.47 ± 0.01 | 2.36 ± 0.05 |
| **Ar14** | 3.040 ± 0.166 | 1.863 ± 0.195 | 1.085 ± 0.109 |
| **Ar15** | 2.32 ± 0.03 | 2.33 ± 0.032 | 1.33 ± 0.06 |
| **Ar16** | 3.44 ± 0.06 | 2.32 ± 0.01 | 1.28 ± 0.19 |
| **Ar17** | 4.64 ± 0.08 | 3.53 ± 0.07 | 2.33 ± 0.02 |
| **Ar18** | 7.51 ± 0.13 | 4.73 ± 0.12 | 3.44 ± 0.02 |
| **Ar19** | 3.35 ± 0.02 | 2.37 ± 0.03 | 0.04 ± 0.01 |
| **Ar20** | 3.01 ± 0.12 | 1.08 ±0.11 | 0.27 ± 0.08 |
| **Ar21** | 3.45 ± 0.06 | 1.28 ± 0.05 | 0.08 ± 0.06 |
| **Ar22** | 0.575 ± 0.0760 | 0.356 ± 0.059 | 0.090 ± 0.029 |
| **Ar23** | 0.196 ± 0.040 | 0.013 ± 0.071 | -0.009 ± 0.058 |
| **Ar24** | 0.618 ± 0.708 | -0.235 ± 0.061 | -0.066 ± 0.024 |
| **PAβN** | 4.71 ± 0.08 | 3.56 ± 0.10 | 1.41 ± 0.02 |
