# Supplementary material for: Pyrrole-based inhibitors of RND-type efflux pumps reverse antibiotic resistance and display anti-virulence potential
Source: PLoS Pathog. 2024 Apr 9;20(4):e1012121. doi: 10.1371/journal.ppat.1012121 (PMC11003683; doi:10.1371/journal.ppat.1012121)
Supplement: S6 Table — (DOCX) [file ppat.1012121.s006.docx]

**S6 Table.** Binding energy (kcal/mol) of lead compounds (Ar1, Ar5, Ar11, Ar18) and PAβN to AcrB and MexB efflux pump protein of *E. coli* and *P. aeruginosa*.

| **Docking with AcrB** | | | |
| --- | --- | --- | --- |
| Compounds | Docking score  (kcal/mol) | Interacting residues | MMGBSA score (kcal//mol) |
| Ar1 | -8.427 | Hydrophobic: Phe136, Val139, Ile626, Phe628, Pro326, Phe610, Tyr327, Val612, Phe615, Phe617, Met573, Val571, Phe178, Ala279, Ile277  Pi-Pi interaction: Phe628, Phe178  Charged (positive): Arg620 | -56.18 |
| Ar5 | -9.795 | Hydrophobic: Tyr327, Val139, Phe136, Val672, Phe178, Leu668, Met573, Phe572, Val571, Val629, Phe628, Phe610, Val612, Ile277, Ala279  Pi-Pi interaction: Phe628, Phe610, Phe178  Polar: Ser134 | -58.85 |
| Ar11 | -9.104 | Hydrophobic: Phe617, Ile277, Phe615, Ala279, Val612, Phe178, Phe610, Ile626, Phe628, Val139, Pro326, Tyr327, Val571, Met573, Phe136  Pi-Pi interaction: Phe178, Phe610, Phe628  Charged (positive): Arg620  Polar: Ser630 | -59.98 |
| Ar18 | -10.168 | Hydrophobic: Tyr327, Val629, Phe628, Val672, Phe136, Val139, Phe610, Val612, Ala279, Phe178, Val571, Phe572, Met573, Leu668  Pi-Pi interaction: Phe628, Phe178, Phe610  Polar: Ser134, Ser135 | -64.98 |
| PAβN | -8.786 | Hydrophobic: Phe615, Val612, Phe610, Pro326, Tyr327, Val571, Phe628, Phe178, Val139, Phe136, Ile277  Pi-Pi interaction: Phe628, Phe136  Charged (positive): Arg620  Polar: Asn274, Gln176, Ser134  Hydrogen bond: Gln176 | -59.08 |
| **Docking with MexB** | | | |
| Compounds | Docking score  (kcal/mol) | Interacting residues | MMGBSA score (kcal//mol) |
| Ar1 | -9.348 | Hydrophobic: Tyr327, Val571, Phe573, Leu672, Val139, Phe136, Met630, Ala290, Phe628, Phe610, Val612, Ala279, Phe615, Ile277, Phe617, Phe178  Pi-Pi interaction: Phe178  Charged (positive): Lys151, Lys134  Polar: Gln176  Hydrogen bond: Gln176 | -61.84 |
| Ar5 | -9.180 | Hydrophobic: Val571, Val139, Phe573, Leu672, Phe136, Tyr327, Ala290, Met630, Phe628, Phe610, Ala279, Val612, Phe615, Phe178, Phe617  Pi-Pi interaction: Phe628, Phe610, Phe178, Tyr327  Charged (positive): Lys134  Polar: Gln176 | -61.37 |
| Ar11 | -9.736 | Hydrophobic: Tyr327, Ala290, Val571, Leu672, Phe136, Phe573, Val139, Met630, Phe628, Phe178, Phe610, Val612, Ala279, Phe615, Phe617, Ala279, Ile277  Pi-Pi interaction: Phe178, Phe610, Phe628  Charged (positive): Lys134, Lys151  Polar: Gln176 | -66.68 |
| Ar18 | -9.839 | Hydrophobic: Phe136, Ala290, Val139, Tyr327, Val571, Phe573, Met630, Phe628, Phe178, Phe610, Leu672, Phe617, Phe615, Ala279, Ile277, Val612  Pi-Pi interaction: Phe628, Phe178, Phe610  Charged (positive): Lys134, Lys151  Polar: Gln176 | -67.08 |
| PAβN | -9.058 | Hydrophobic: Val139, Phe136, Met630, Phe628, Leu672, Phe178, Ala279, Ile277, Phe610, Val612, Phe615, Phe617, Phe573, Val571, Tyr327  Pi-Pi interaction: Phe178, Phe610  Charged (positive): Lys134  Charged (negative): Gln81  Polar: Gln176, Asn616  Hydrogen bond: Lys134, Gln176, Gln81, Asn616 | -69.41 |
