# Supplementary material for: Pyrrole-based inhibitors of RND-type efflux pumps reverse antibiotic resistance and display anti-virulence potential
Source: PLoS Pathog. 2024 Apr 9;20(4):e1012121. doi: 10.1371/journal.ppat.1012121 (PMC11003683; doi:10.1371/journal.ppat.1012121)
Supplement: S7 Table — (DOCX) [file ppat.1012121.s007.docx]

**S7 Table.** Binding energy (kcal/mol) of lead compounds (Ar1, Ar5, Ar11, Ar18) and MBX3135 to AcrB and MexB mutants.

|  | **AcrB^F178A^** | **AcrB^F628A^** | **AcrB^F615A, F617A, R620A^** | **MexB^F178A^** | **MexB^F628A^** | **MexB^F615A, F617A, R620A^** |
| --- | --- | --- | --- | --- | --- | --- |
| Compounds | Docking score (kcal/mol) | Docking score (kcal/mol) | Docking score (kcal/mol) | Docking score (kcal/mol) | Docking score (kcal/mol) | Docking score (kcal/mol) |
| Ar1 | -8.50 | -8.63 | -9.15 | -9.93 | -8.99 | -9.33 |
| Ar5 | -7.85 | -9.01 | -9.03 | -9.93 | -9.50 | -9.18 |
| Ar11 | -8.64 | -9.17 | -9.89 | -10.17 | -11.44 | -9.25 |
| Ar18 | -8.99 | -9.17 | -9.49 | -10.68 | -8.74 | -9.79 |
| MBX3135 | -9.21 | -8.83 | -8.91 | -11.62 | -7.38 | -9.34 |
