# Supplementary material for: Pyrrole-based inhibitors of RND-type efflux pumps reverse antibiotic resistance and display anti-virulence potential
Source: PLoS Pathog. 2024 Apr 9;20(4):e1012121. doi: 10.1371/journal.ppat.1012121 (PMC11003683; doi:10.1371/journal.ppat.1012121)
Supplement: S8 Table — (DOCX) [file ppat.1012121.s008.docx]

**S8 Table.** Cytotoxicity profile of synthesized compounds (Ar1-Ar24) on human peripheral blood mononuclear cells (H-PBMCs) as determined by using the MTT assay. The experiment was performed in two biological replicates, the average of triplicates ± SD is shown.

| Compound | Concentration (µg/mL) | Viability ± SD (%) | IC_50_ (μg/mL) | Safety index |
| --- | --- | --- | --- | --- |
| Ar1 | 256 | 51.25 ± 6.3 | 256 | 16 |
|  | 128 | 74.12 ± 1.9 |  |  |
|  | 64 | 85.75 ± 4.9 |  |  |
| Ar2 | 256 | 48.10 ± 1.0 | 256 | 16 |
|  | 128 | 73.28 ± 0.5 |  |  |
|  | 64 | 93.27 ± 17.3 |  |  |
| Ar3 | 256 | 41.67 ± 1.0 | >128 | >8 |
|  | 128 | 71.76 ± 1.6 |  |  |
|  | 64 | 76.3 ± 0.7 |  |  |
| Ar4 | 256 | 98.02 ± 2.7 | >256 | >16 |
|  | 128 | 105.30 ± 9.0 |  |  |
|  | 64 | 117.61 ± 7.2 |  |  |
| Ar5 | 256 | 51.05 ± 1.0 | 256 | 16 |
|  | 128 | 68.65 ± 9.4 |  |  |
|  | 64 | 86.75 ± 5.1 |  |  |
| Ar6 | 256 | 47.16 ± 1.4 | 256 | 16 |
|  | 128 | 74.24 ± 1.4 |  |  |
|  | 64 | 89.26 ± 0.6 |  |  |
| Ar7 | 256 | 45.18 ± 1.4 | 256 | 16 |
|  | 128 | 74.24 ± 2.5 |  |  |
|  | 64 | 88.50 ± 1.2 |  |  |
| Ar8 | 256 | 47.40 ± 2.5 | 256 | 16 |
|  | 128 | 72.82 ± 1.3 |  |  |
|  | 64 | 87.14 ± 4.1 |  |  |
| Ar9 | 256 | 59.39 ± 8.3 | >256 | >16 |
|  | 128 | 79.64 ± 2.2 |  |  |
|  | 64 | 87.81 ± 2.5 |  |  |
| Ar10 | 256 | 48.22 ± 0.3 | 256 | 16 |
|  | 128 | 69.48 ± 0.9 |  |  |
|  | 64 | 75.42 ± 1.9 |  |  |
| Ar11 | 256 | 90.03 ± 0.9 | >256 | >16 |
|  | 128 | 93.30 ± 1.6 |  |  |
|  | 64 | 104.41 ± 15.9 |  |  |
| Ar12 | 256 | 49.75 ± 3.3 | 256 | 16 |
|  | 128 | 77.84 ± 1.3 |  |  |
|  | 64 | 91.18 ± 0.3 |  |  |
| Ar13 | 256 | 47.66 ± 1.0 | 256 | 16 |
|  | 128 | 76.07 ± 3.0 |  |  |
|  | 64 | 93.64 ± 4.6 |  |  |
| Ar14 | 256 | 44.80 ± 1.4 | >128 | >8 |
|  | 128 | 72.00 ± 0.9 |  |  |
|  | 64 | 85.40 ± 2.8 |  |  |
| Ar15 | 256 | 52.56 ± 1.8 | 256 | 16 |
|  | 128 | 69.99 ± 0.8 |  |  |
|  | 64 | 85.21 ± 1.0 |  |  |
| Ar16 | 256 | 50.50 ± 0.3 | 256 | 16 |
|  | 128 | 78.34 ± 1.4 |  |  |
|  | 64 | 89.70 ± 0.4 |  |  |
| Ar17 | 256 | 46.12 ± 4.5 | 256 | 16 |
|  | 128 | 67.95 ± 3.0 |  |  |
|  | 64 | 79.91 ± 7.8 |  |  |
| Ar18 | 256 | 41.98 ± 1.2 | >128 | >8 |
|  | 128 | 69.12 ± 0.6 |  |  |
|  | 64 | 82.70 ± 1.5 |  |  |
| Ar19 | 256 | 42.41 ± 1.2 | >128 | >8 |
|  | 128 | 70.16 ± 2.2 |  |  |
|  | 64 | 82.68 ± 1.9 |  |  |
| Ar20 | 256 | 41.20 ± 0.8 | >128 | >8 |
|  | 128 | 65.55 ± 1.8 |  |  |
|  | 64 | 76.60 ± 2.2 |  |  |
| Ar21 | 256 | 46.06 ± 4.4 | 256 | 16 |
|  | 128 | 68.83 ± 1.7 |  |  |
|  | 64 | 86.48 ± 4.9 |  |  |
| Ar22 | 256 | 37.10 ± 0.7 | >128 | >8 |
|  | 128 | 61.70 ± 1.5 |  |  |
|  | 64 | 82.76 ± 2.2 |  |  |
| Ar23 | 256 | 42.74 ± 0.4 | >128 | >8 |
|  | 128 | 70.46 ± 1.9 |  |  |
|  | 64 | 92.54 ± 6.5 |  |  |
| Ar24 | 256 | 45.53 ± 2.6 | 256 | 16 |
|  | 128 | 74.67 ± 1.2 |  |  |
|  | 64 | 86.95 ± 2.7 |  |  |

IC_50_- The concentration of an inhibitor at which approximately 50% cell viability is achieved.
