# Supplementary material for: Pyrrole-based inhibitors of RND-type efflux pumps reverse antibiotic resistance and display anti-virulence potential
Source: PLoS Pathog. 2024 Apr 9;20(4):e1012121. doi: 10.1371/journal.ppat.1012121 (PMC11003683; doi:10.1371/journal.ppat.1012121)
Supplement: S9 Table — (DOCX) [file ppat.1012121.s009.docx]

**S9 Table.** Hemolytic activity of the synthesized compounds (Ar1-Ar24) on rabbit blood erythrocytes. The experiment was performed in two biological replicates and three technical replicates, the average of triplicates ± SD is shown.

| Compounds | Concentration (μg/mL) | Hemolysis ± SD (%) | HC_10_ (μg/mL) | Hemolysis Index |
| --- | --- | --- | --- | --- |
| Ar1 | 512 | 4.48 ± 0.56 | >512 | >32 |
|  | 256 | 0.15 ± 1.34 |  |  |
| Ar2 | 512 | 6.78 ± 1.6 | >512 | >32 |
|  | 256 | 3.75 ± 1.46 |  |  |
| Ar3 | 512 | 5.97 ± 2.63 | >512 | >32 |
|  | 256 | 2.75 ± 1.19 |  |  |
| Ar4 | 512 | 61.57 ± 6.91 | ~64 | ~4 |
|  | 256 | 39.31 ± 7.31 |  |  |
|  | 128 | 26.77 ± 8.21 |  |  |
|  | 64 | 7.28 ± 2.42 |  |  |
| Ar5 | 512 | - | >512 | >32 |
|  | 256 | - |  |  |
| Ar6 | 512 | 5.63 ± 0.9 | >512 | >32 |
|  | 256 | 4.32 ± 0.23 |  |  |
| Ar7 | 512 | 24.29 ± 3.8 | >64-<128 | >4-<8 |
|  | 256 | 15.78 ± 3.54 |  |  |
|  | 128 | 18.66 ± 2.22 |  |  |
|  | 64 | 4.98 ± 0.43 |  |  |
| Ar8 | 512 | 8.88 ± 0.85 | >512 | >32 |
|  | 256 | 5.59 ± 2.90 |  |  |
| Ar9 | 512 | 19.88 ± 2.74 | 128 | 8 |
|  | 256 | 12.49 ± 4.22 |  |  |
|  | 128 | 9.13 ± 0.61 |  |  |
| Ar10 | 512 | 7.39 ± 2.71 | >512 | >32 |
|  | 256 | 2.98 ± 1.6 |  |  |
| Ar11 | 512 | 37.16 ± 5.92 | <64 | <4 |
|  | 256 | 34.94 ± 0.46 |  |  |
|  | 128 | 31.50 ± 3.77 |  |  |
|  | 64 | 27.45 ± 0.56 |  |  |
| Ar12 | 512 | 7.47 ± 1.78 | >512 | >32 |
|  | 256 | 6.78 ± 3.59 |  |  |
| Ar13 | 512 | 2.18 ± 1.08 | >512 | >32 |
|  | 256 | 1.3 ± 0.57 |  |  |
| Ar14 | 512 | 9.96 ± 4.81 | 512 | 32 |
|  | 256 | 5.28 ± 0.76 |  |  |
| Ar15 | 512 | 29.11 ± 2.87 | >64-<128 | >4-<8 |
|  | 256 | 22.18 ± 3.01 |  |  |
|  | 128 | 15.92 ± 3.94 |  |  |
|  | 64 | 2.29 ± 0.3 |  |  |
| Ar16 | 512 | 3.94 ± 1.37 | >512 | >32 |
|  | 256 | 3.40 ± 3.15 |  |  |
| Ar17 | 512 | 26.43 ± 2.39 | 256 | 16 |
|  | 256 | 10.26 ± 5.23 |  |  |
| Ar18 | 512 | 1.03 ± 0.24 | >512 | >32 |
|  | 128 | - |  |  |
| Ar19 | 512 | 32.72 ± 6.22 | 256 | 16 |
|  | 256 | 11.8 ± 2.68 |  |  |
| Ar20 | 512 | 15.47 ± 2.44 | >256-<512 | >16-<32 |
|  | 256 | 2.29 ± 2.18 |  |  |
| Ar21 | 512 | 1.87 ± 0.19 | >512 | >32 |
|  | 256 | - |  |  |
| Ar22 | 512 | 78.27 ± 4.72 | ~128 | ~8 |
|  | 256 | 41.76 ± 4.69 |  |  |
|  | 128 | 8.01 ± 0.3 |  |  |
| Ar23 | 512 | 2.68 ± 0.42 | >512 | >32 |
|  | 256 | - |  |  |
| Ar24 | 512 | 3.02 ± 0.48 | >512 | >32 |
|  | 256 | 1.26 ± 0.74 |  |  |
| Triton X-100 | 0.1% | 100.32 ± 7.02 | NA | NA |

HC_10_- Minimal hemolytic concentration that approximately induced a 10% hemolysis; '-' represents no hemolysis; NA, Not applicable
