# Supplementary material for: Pyrrole-based inhibitors of RND-type efflux pumps reverse antibiotic resistance and display anti-virulence potential
Source: PLoS Pathog. 2024 Apr 9;20(4):e1012121. doi: 10.1371/journal.ppat.1012121 (PMC11003683; doi:10.1371/journal.ppat.1012121)
Supplement: S10 Table — (DOCX) [file ppat.1012121.s010.docx]

**S10 Table.** Differential gene expression of the virulence factor genes of *P. aeruginosa* PAO1 in response to the treatment of Ar5 (16 µg/mL) and PAβN (16 µg/mL, positive control) compared to the untreated control. The experiment was performed in two biological replicates, values of both biological replicates are represented.

| Genes | Function | Log_2_ Fold Change^*^ | | Log_2_ Scale Value | | | | | |
| --- | --- | --- | --- | --- | --- | --- | --- | --- | --- |
|  |  | PAβN | Ar5 | Control | | PAβN | | Ar5 | |
|  |  |  |  | Replicate 1 | Replicate 2 | Replicate 1 | Replicate 2 | Replicate 1 | Replicate 2 |
| phzD2 | Phenazine biosynthesis | 1.65 | -0.0007 | 5.71 | 5.68 | 7.05 | 7.68 | 6.12 | 5.14 |
| phzE1 |  | 1.11 | -0.39 | 6.12 | 6.09 | 7.15 | 6.77 | 5.06 | 5.74 |
| phzF2 |  | 0.66 | -0.29 | 6.03 | 6.005 | 6.67 | 6.97 | 6.08 | 5.53 |
| phzD1 |  | 1.39 | -0.09 | 3.56 | 3.53 | 5.23 | 4.34 | 3.21 | 3.45 |
| phzF1 |  | 0.62 | -2.1 | 3.56 | 3.53 | 4.14 | 4.34 | -0.10 | 2.28 |
| phzB1 |  | 0.39 | -0.78 | 13.44 | 13.41 | 14.07 | 14.55 | 13.02 | 13.21 |
| phzM |  | 1.41 | -0.01 | 13.58 | 13.55 | 14.88 | 14.97 | 13.44 | 13.49 |
| phzB2 |  | 1.66 | -0.27 | 13.9 | 13.87 | 15.12 | 15.70 | 13.42 | 13.51 |
| phzS |  | 0.57 | -0.59 | 13.99 | 13.96 | 15.00 | 15.39 | 14.11 | 13.89 |
| phzE2 |  | 0.54 | -0.95 | 8.49 | 8.46 | 8.11 | 9.11 | 7.28 | 7.04 |
| phzC1 |  | 0.76 | -0.61 | 10.7 | 10.67 | 11.68 | 12.13 | 10.44 | 10.59 |
| phzC2 |  | 1.8 | -0.2 | 10.78 | 10.75 | 12.04 | 12.63 | 10.39 | 10.24 |
| phzG2 |  | 1.53 | 0.17 | 10.41 | 10.38 | 11.66 | 12.17 | 10.71 | 10.36 |
| phzA2 |  | 0.27 | -0.1 | 11.54 | 11.51 | 11.36 | 11.80 | 11.09 | 11.29 |
| phzA1 |  | -0.33 | -0.24 | 11.63 | 11.6 | 11.20 | 11.32 | 11.34 | 11.29 |
| phzG1 |  | 0.24 | -0.48 | 10.37 | 10.34 | 10.98 | 11.21 | 10.42 | 10.26 |
| phzH |  | 0.65 | 0.005 | 12.63 | 12.6 | 13.13 | 13.28 | 12.61 | 12.45 |
| pchE | Pyochelin biosynthesis | 1.38 | 1.04 | 15.31 | 15.28 | 16.49 | 16.70 | 16.25 | 16.19 |
| pchF |  | 0.21 | 0.94 | 15.89 | 15.86 | 16.03 | 16.32 | 16.87 | 16.89 |
| pchD |  | 2.39 | 1.52 | 14.48 | 14.45 | 16.43 | 16.46 | 15.60 | 15.49 |
| pchA |  | 1.35 | 1.33 | 14.08 | 14.05 | 15.00 | 15.26 | 15.24 | 14.90 |
| pchR |  | 1.81 | 0.58 | 12.27 | 12.24 | 13.85 | 13.93 | 12.47 | 12.78 |
| pchC |  | 1.74 | 1.41 | 12.59 | 12.56 | 13.92 | 13.99 | 13.69 | 13.50 |
| pchB |  | 1.6 | 1.44 | 12.76 | 12.74 | 13.85 | 14.18 | 13.81 | 13.84 |
| pchG |  | 0.3 | 0.98 | 12.85 | 12.82 | 12.88 | 13.26 | 13.73 | 13.74 |
| pchP |  | -0.19 | -0.03 | 12.37 | 12.34 | 12.13 | 12.18 | 12.32 | 12.25 |
| fptA | Pyochelin receptor | 2.54 | 1.07 | 15.54 | 15.51 | 17.83 | 17.85 | 16.38 | 16.29 |
| aprA | Alkaline protease | 0.54 | 0.58 | 15.99 | 15.97 | 16.12 | 16.38 | 16.34 | 16.18 |
| lon | Lon protease | 0.39 | -0.05 | 17.04 | 17.01 | 17.46 | 17.43 | 16.91 | 17.03 |
| lasB | Elastase | 0.54 | 0.4 | 16.58 | 16.55 | 16.77 | 16.96 | 16.68 | 16.71 |
| lasA |  | 1 | 0.18 | 17.84 | 17.81 | 18.19 | 18.22 | 17.17 | 17.53 |
| lasR |  | 0.03 | 0.16 | 15.54 | 15.51 | 15.50 | 15.44 | 15.53 | 15.60 |
| lasI |  | -0.09 | -0.34 | 16.4 | 16.37 | 16.13 | 16.27 | 15.85 | 15.99 |
| rhlB | Rhamnolipid biosynthesis/Quorum sensing | 0.86 | 0.1 | 16.33 | 16.3 | 16.87 | 16.73 | 15.93 | 16.08 |
| rhlR |  | 0.35 | 0.08 | 16.66 | 16.63 | 16.80 | 16.68 | 16.36 | 16.53 |
| rhlA |  | 0.9 | -0.09 | 17.02 | 16.99 | 17.51 | 17.41 | 16.33 | 16.54 |
| rhlC |  | 0.49 | 0.09 | 13.93 | 13.9 | 14.11 | 14.12 | 13.55 | 13.82 |
| rhlI |  | -0.4 | 0.07 | 14.55 | 14.52 | 14.00 | 13.96 | 14.34 | 14.51 |
| pslA | Biofilm formation | 0.07 | -0.08 | 14.47 | 14.44 | 14.59 | 14.48 | 14.27 | 14.43 |
| PA4541 | Protease (lepA) | 0.03 | 0.02 | 14.06 | 14.03 | 14.02 | 14.06 | 14.01 | 14.00 |
| pilH | Pili biosynthesis | -0.41 | -0.43 | 14.15 | 14.12 | 13.96 | 13.94 | 13.82 | 13.98 |
| pilG |  | -0.5 | -0.4 | 16.05 | 16.02 | 15.59 | 15.55 | 15.39 | 15.85 |
| pilJ |  | -0.26 | -0.35 | 14.99 | 14.96 | 14.92 | 14.91 | 14.71 | 14.86 |
| pilK |  | -0.29 | -0.36 | 12.46 | 12.43 | 12.23 | 12.23 | 12.13 | 12.13 |
| pilI |  | -0.59 | -0.42 | 13.28 | 13.26 | 12.99 | 12.92 | 12.91 | 13.25 |
| pilV |  | -0.17 | 0.13 | 11.78 | 11.75 | 11.66 | 11.65 | 11.97 | 11.90 |
| pilW |  | -0.19 | 0.04 | 12.54 | 12.51 | 12.49 | 12.56 | 12.78 | 12.70 |
| pilX |  | -0.02 | 0.07 | 12.26 | 12.23 | 12.35 | 12.41 | 12.49 | 12.42 |
| pilY1 |  | -0.28 | -0.07 | 15.39 | 15.37 | 15.26 | 15.31 | 15.47 | 15.45 |
| pilY2 |  | -0.35 | -0.17 | 11.19 | 11.16 | 10.85 | 11.03 | 11.21 | 10.98 |
| pilE |  | -0.21 | -0.11 | 11.61 | 11.58 | 11.54 | 11.68 | 11.86 | 11.48 |
| pilQ |  | -0.61 | -0.46 | 15.61 | 15.58 | 15.42 | 15.40 | 15.53 | 15.54 |
| pilP |  | -0.76 | -0.42 | 12.8 | 12.77 | 12.52 | 12.40 | 12.70 | 12.83 |
| pilO |  | -0.64 | -0.40 | 13.2 | 13.17 | 12.98 | 12.90 | 13.18 | 13.11 |
| pilN |  | -0.68 | -0.48 | 13.28 | 13.25 | 13.10 | 12.97 | 13.17 | 13.23 |
| pilM |  | -0.60 | -0.54 | 16.17 | 16.14 | 15.83 | 15.65 | 15.68 | 15.86 |
| PilT |  | -0.22 | -0.10 | 14.18 | 14.15 | 13.92 | 13.91 | 13.91 | 14.08 |
| pilU |  | -0.47 | -0.24 | 14.91 | 14.88 | 14.49 | 14.35 | 14.58 | 14.66 |
| pilZ |  | -0.31 | -0.15 | 13.49 | 13.46 | 13.39 | 13.01 | 13.31 | 13.47 |
| pilF |  | 0.44 | -0.06 | 12.84 | 12.81 | 13.20 | 13.15 | 12.61 | 12.65 |
| pilA |  | -0.80 | 0.01 | 17.61 | 17.58 | 16.96 | 16.90 | 17.61 | 17.82 |
| pilB |  | -0.56 | -0.10 | 14.81 | 14.78 | 14.40 | 14.39 | 14.79 | 14.86 |
| pilC |  | -0.61 | -0.51 | 16.61 | 16.58 | 16.19 | 16.13 | 16.20 | 16.28 |
| pilD |  | -0.49 | -0.31 | 12.86 | 12.83 | 12.59 | 12.48 | 12.68 | 12.69 |
| pilS |  | -0.32 | -0.11 | 13.08 | 13.05 | 12.76 | 12.79 | 12.93 | 12.99 |
| pilR |  | 0.11 | -0.07 | 12.48 | 12.45 | 12.61 | 12.64 | 12.38 | 12.42 |
| pqsE | Quinolone signal response | 0.68 | 0.29 | 14.03 | 14 | 14.65 | 14.35 | 13.79 | 14.20 |
| pqsA |  | -0.08 | 0.38 | 15.4 | 15.37 | 15.65 | 15.15 | 15.72 | 15.98 |
| pqsB |  | 0.37 | 0.28 | 15.12 | 15.09 | 15.60 | 15.28 | 15.20 | 15.45 |
| pqsC |  | 0.43 | 0.19 | 14.64 | 14.61 | 15.13 | 14.90 | 14.69 | 14.80 |
| pqsD |  | 0.50 | 0.24 | 14.74 | 14.71 | 15.30 | 15.04 | 14.80 | 14.98 |
| pqsH |  | 0.63 | 0.15 | 13.34 | 13.31 | 13.72 | 13.76 | 13.23 | 13.22 |
| pqsL |  | 0.007 | -0.03 | 12.35 | 12.32 | 12.52 | 12.56 | 12.54 | 12.40 |
| pvdL | Pyoverdine biosynthesis | -0.19 | 1.12 | 14.73 | 14.7 | 14.08 | 14.16 | 15.48 | 15.34 |
| pvdD |  | -0.22 | 0.61 | 14.65 | 14.62 | 14.09 | 14.23 | 15.05 | 14.90 |
| pvdJ |  | -0.42 | 0.5 | 14.37 | 14.34 | 13.69 | 13.76 | 14.73 | 14.52 |
| pvdG |  | 0.57 | 0.65 | 10.95 | 10.92 | 11.21 | 11.47 | 11.46 | 11.33 |
| pvdO |  | 0.04 | 0.88 | 11.28 | 11.25 | 10.80 | 11.03 | 11.83 | 11.63 |
| pvdH |  | 0.41 | 0.87 | 12.28 | 12.25 | 12.15 | 12.32 | 12.71 | 12.63 |
| pvdN |  | 0.26 | 0.7 | 12.18 | 12.15 | 12.05 | 12.27 | 12.62 | 12.53 |
| pvdQ |  | 0.17 | 0.48 | 12.14 | 12.11 | 12.01 | 12.14 | 12.46 | 12.26 |
| pvdP |  | 0.02 | 0.26 | 12.67 | 12.64 | 12.45 | 12.69 | 12.84 | 12.73 |
| pvdS |  | 1.45 | 0.95 | 12.04 | 12.01 | 13.11 | 13.33 | 12.65 | 12.73 |
| pvdF |  | -0.06 | 0.4 | 12.76 | 12.73 | 12.49 | 12.50 | 13.01 | 12.85 |
| pvdA |  | 0.74 | 1.29 | 13.37 | 13.34 | 13.44 | 13.63 | 14.14 | 13.96 |
| pvdE |  | -0.15 | 0.17 | 13.59 | 13.56 | 13.26 | 13.42 | 13.63 | 13.65 |
| pvdR |  | 0.56 | 0.38 | 11.36 | 11.33 | 11.81 | 11.88 | 11.64 | 11.63 |
| pvdT |  | 0.27 | 0.32 | 11.77 | 11.74 | 11.85 | 12.01 | 12.01 | 11.89 |
| fpvA | Ferripyoverdine receptor | 0.23 | 0.41 | 14.07 | 14.04 | 14.05 | 14.16 | 14.28 | 14.22 |
| fpvB |  | -0.11 | 0.14 | 13.44 | 13.41 | 13.17 | 13.24 | 13.36 | 13.51 |
| chpA | Type IV pili twitching motility | -0.08 | -0.32 | 15.62 | 15.59 | 12.02 | 12.17 | 15.42 | 15.50 |
| chpE |  | -0.1 | -0.17 | 9.93 | 9.9 | 10.00 | 10.13 | 10.11 | 9.81 |
| chpC |  | 0.0005 | -0.28 | 11.05 | 11.02 | 11.33 | 11.44 | 11.13 | 11.00 |
| chpD |  | -0.2 | -0.23 | 10.44 | 10.41 | 10.49 | 10.67 | 10.52 | 10.53 |
| chpB |  | -0.22 | -0.31 | 11.94 | 11.92 | 12.02 | 12.17 | 11.93 | 12.02 |
| pasP | Small protease | 0.32 | 0.13 | 14.34 | 14.32 | 14.84 | 15.00 | 14.81 | 14.60 |
| pelC | Pellicle/biofilm formation | 0.02 | -0.01 | 9.21 | 9.18 | 9.29 | 9.31 | 9.22 | 9.44 |
| pelF |  | -0.16 | 0.1 | 11.55 | 11.52 | 11.32 | 11.39 | 11.62 | 11.57 |
| pelD |  | 0.02 | 0.07 | 10.68 | 10.65 | 10.62 | 10.84 | 10.72 | 10.78 |
| pelE |  | 0.001 | 0.16 | 10.86 | 10.83 | 10.87 | 10.83 | 11.03 | 10.94 |
| pelB |  | -0.03 | 0.03 | 11.98 | 11.95 | 11.86 | 12.00 | 12.00 | 11.94 |
| pelG |  | -0.47 | -0.14 | 12.49 | 12.46 | 11.93 | 12.07 | 12.28 | 12.35 |
| pelA |  | 0.08 | 0.06 | 12.26 | 12.23 | 12.30 | 12.32 | 12.27 | 12.29 |
| toxA | ExotoxinA | -0.29 | -0.03 | 11.84 | 11.81 | 11.46 | 11.36 | 11.67 | 11.60 |
| plcH | Phospholipase C | -0.41 | -0.01 | 12.72 | 12.69 | 12.20 | 12.39 | 12.63 | 12.70 |
| qscR | Quorum sensing | 0.06 | -0.25 | 13.16 | 13.13 | 12.98 | 12.97 | 12.53 | 12.70 |

^*^The positive and negative values of Log_2_ fold change represent the up-regulation and down-regulation of genes respectively.
