# Supplementary material for: Pyrrole-based inhibitors of RND-type efflux pumps reverse antibiotic resistance and display anti-virulence potential
Source: PLoS Pathog. 2024 Apr 9;20(4):e1012121. doi: 10.1371/journal.ppat.1012121 (PMC11003683; doi:10.1371/journal.ppat.1012121)
Supplement: S11 Table — (DOCX) [file ppat.1012121.s011.docx]

**S11 Table.** Acute toxicity of compound Ar5 in BALB/c mice.

| Parameters | Vehicle | 100 mg/kg | 500 mg/kg | 750 mg/kg | 1000 mg/kg |
| --- | --- | --- | --- | --- | --- |
| Body weight (g) | 15.75 | 14.69 | 16.11 | 14.67 | 15.4 |
| Glucose (mg/dL) | 67 | 102 | 88 | 103 | 61 |
| Creatinine (mg/dL) | ≤1 | ≤1 | ≤1 | ≤0.5 | ≤1 |
| Triglycerides (mg/dL) | 85 | 65 | 110 | 47.5 | 55 |
| Cholesterol (mg/dL) | 75 | 100 | 90 | 107.5 | 70 |
| Bilirubin; conjugated (mg/dL) | 0.45 | 0.4 | 0.5 | 0.25 | 0.5 |
| Bilirubin; unconjugated (mg/dL) | 0.55 | 0.35 | 0.25 | 0.2 | 0.5 |
| SGOT (U/L) | 100 | 100 | 110 | 92.5 | 280 |
| SGPT (U/L) | 40 | 60 | 45 | 40 | 45 |
| Alkaline phosphatase (U/L) | 100 | 110 | 90 | 85 | 60 |
| Total proteins (g/dL) | 4 | 5 | 5 | 4.5 | 4 |
| Albumin (g/dL) | 2.5 | 3 | 3 | 2.5 | 2 |
| Globulin (g/dL) | 1.5 | 2 | 2 | 2 | 2 |
| A/G ratio | 1.7 | 1.5 | 1.5 | 1.3 | 1.0 |
