# Supplementary material for: Pyrrole-based inhibitors of RND-type efflux pumps reverse antibiotic resistance and display anti-virulence potential
Source: PLoS Pathog. 2024 Apr 9;20(4):e1012121. doi: 10.1371/journal.ppat.1012121 (PMC11003683; doi:10.1371/journal.ppat.1012121)
Supplement: S12 Table — (DOCX) [file ppat.1012121.s012.docx]

**S12 Table.** *In silico* ADME analyses.

| Compounds | XLogP3 | LogS (ESOL) | GI absorption | BBB permeant | P-gp substrate | CYP3A4 inhibitor | Log K_p_(cm/s) | Lipinski violation | Bioavailability score |
| --- | --- | --- | --- | --- | --- | --- | --- | --- | --- |
| Ar1 | 4.53 | -4.89 | High | Yes | No | No | -5.06 | None | 0.55 |
| Ar2 | 4.88 | -5.12 | High | Yes | No | Yes | -5.15 | None | 0.55 |
| Ar3 | 4.14 | -4.66 | High | Yes | No | Yes | -5.43 | None | 0.55 |
| Ar4 | 4.08 | -4.60 | High | Yes | No | No | -5.36 | None | 0.55 |
| Ar5 | 4.27 | -4.75 | High | Yes | No | No | -5.26 | None | 0.55 |
| Ar6 | 4.84 | -5.08 | High | Yes | No | Yes | -5.00 | None | 0.55 |
| Ar7 | 4.51 | -4.87 | High | Yes | No | No | -5.14 | None | 0.55 |
| Ar8 | 3.79 | -4.38 | High | Yes | No | Yes | -5.54 | None | 0.55 |
| Ar9 | 4.95 | -5.25 | High | No | No | No | -5.20 | None | 0.85 |
| Ar10 | 4.05 | -4.65 | High | Yes | No | Yes | -5.72 | None | 0.55 |
| Ar11 | 3.61 | -4.37 | High | Yes | No | Yes | -5.95 | None | 0.55 |
| Ar12 | 3.75 | -4.38 | High | Yes | No | Yes | -5.67 | None | 0.55 |
| Ar13 | 4.13 | -4.64 | High | Yes | No | Yes | -5.51 | None | 0.55 |
| Ar14 | 4.65 | -5.11 | High | Yes | No | Yes | -5.31 | None | 0.55 |
| Ar15 | 4.50 | -5.03 | High | No | No | No | -5.43 | None | 0.85 |
| Ar16 | 5.42 | -5.67 | Low | No | No | No | -4.84 | Yes (1) | 0.55 |
| Ar17 | 2.99 | -3.38 | High | Yes | No | No | -5.60 | None | 0.55 |
| Ar18 | 4.74 | -5.14 | High | No | No | No | -5.13 | Yes (1) | 0.55 |
| Ar19 | 3.60 | -4.34 | High | Yes | No | Yes | -5.83 | None | 0.55 |
| Ar20 | 3.65 | -4.35 | High | Yes | No | Yes | -5.86 | None | 0.55 |
| Ar21 | 4.26 | -4.77 | High | Yes | No | Yes | -5.40 | None | 0.55 |
| Ar22 | 4.46 | -4.85 | High | Yes | No | No | -5.28 | None | 0.55 |
| Ar23 | 4.53 | -4.89 | High | Yes | No | No | -5.06 | None | 0.55 |
| Ar24 | 5.69 | -5.81 | Low | No | No | No | -4.62 | Yes (1) | 0.55 |
| PAβN | 3.96 | -5.04 | Low | No | No | Yes | -6.66 | Yes (2) | 0.17 |
| Reserpine | 4.04 | -5.75 | High | No | Yes | No | -7.14 | Yes (2) | 0.17 |

ESOL, Estimating aqueous solubility; GI, Gastrointestinal; BBB, Blood-brain barrier; P-gp, P-glycoprotein; CYP3A4, Cytochrome P450 3A4
