# Supplementary figures and images for: Pyrrole-based inhibitors of RND-type efflux pumps reverse antibiotic resistance and display anti-virulence potential
Source: PLoS Pathog. 2024 Apr 9;20(4):e1012121. doi: 10.1371/journal.ppat.1012121 (PMC11003683; doi:10.1371/journal.ppat.1012121)

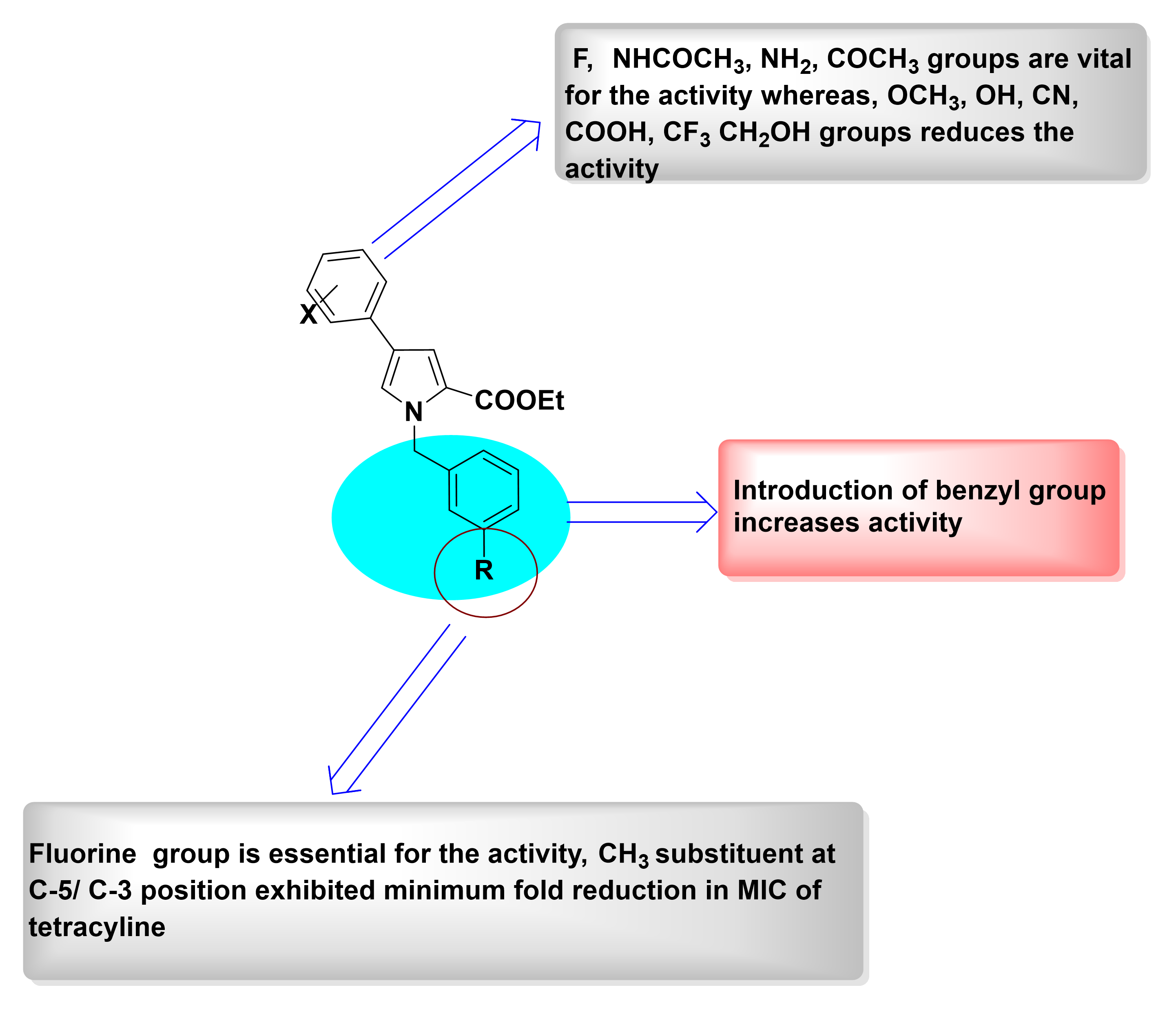

Supplement: S1 Fig — (TIF) [file ppat.1012121.s013.tif]

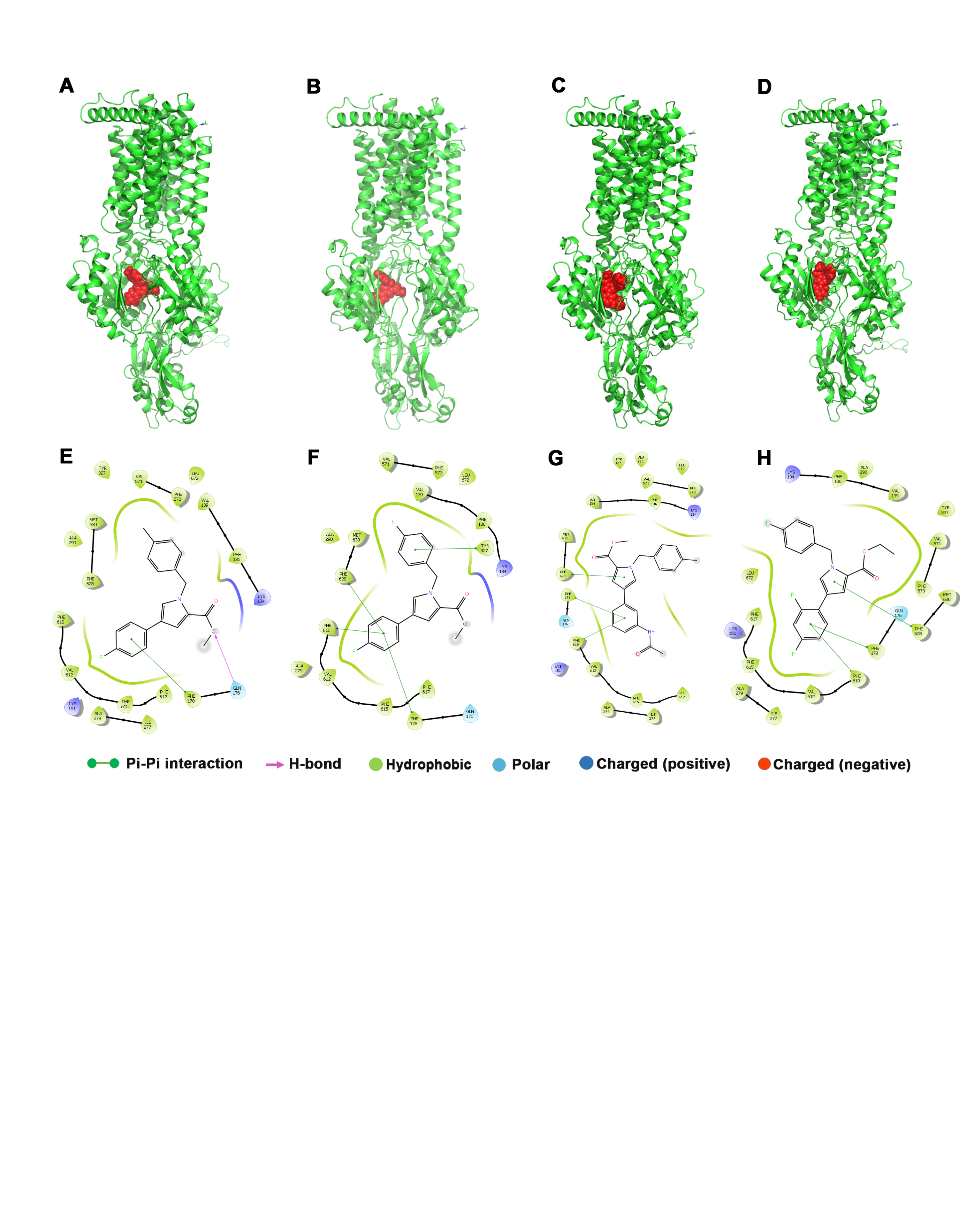

Supplement: S2 Fig — Structural representation of Ar series ligands (red spheres) docked in MexB (green cartoon) (A) Ar1 (B) Ar5 (C) Ar11 (D) Ar18 docked in the active site cleft of MexB. 2D ligand interaction diagram portraying various interactions involved in binding of (E) Ar1 (F) Ar5 (G) Ar11 (H) Ar18 to MexB. (TIF) [file ppat.1012121.s014.tif]

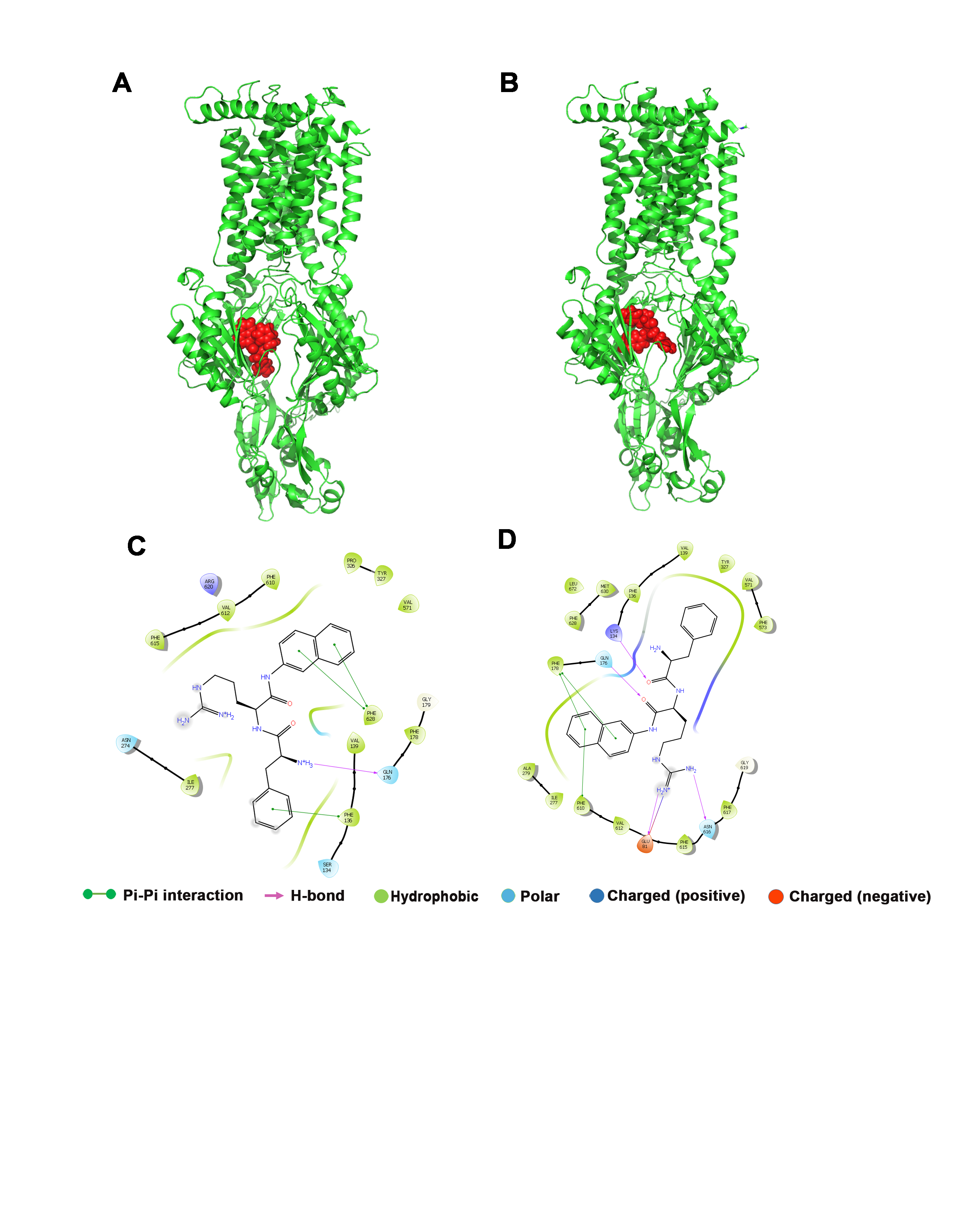

Supplement: S3 Fig — PAβN (red spheres) docked in the active site cleft of (A) AcrB (green cartoon) and (B) MexB (green cartoon). 2D ligand interaction diagram portraying various interactions involved in binding of PAβN to (C) AcrB and (D) MexB. (TIF) [file ppat.1012121.s015.tif]

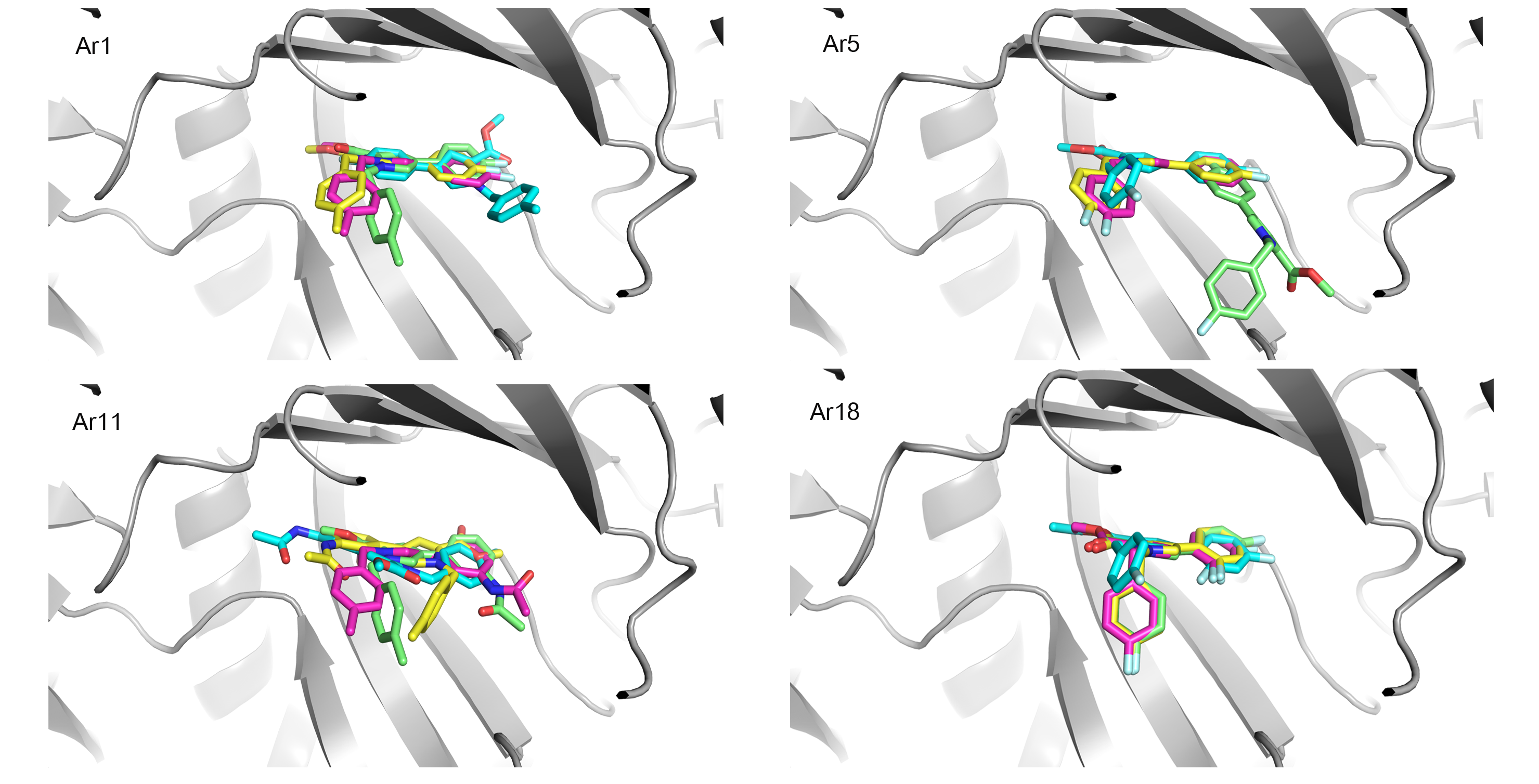

Supplement: S4 Fig — For clarity, AcrB is shown in cartoon representation and ligands are shown in stick representation. The Ar compounds are coloured in pink (docked with AcrB), light green (docked with AcrBF178A), yellow (docked with AcrBF628A), and cyan (docked with AcrBF615A, F617A, R620A). (TIF) [file ppat.1012121.s016.tif]

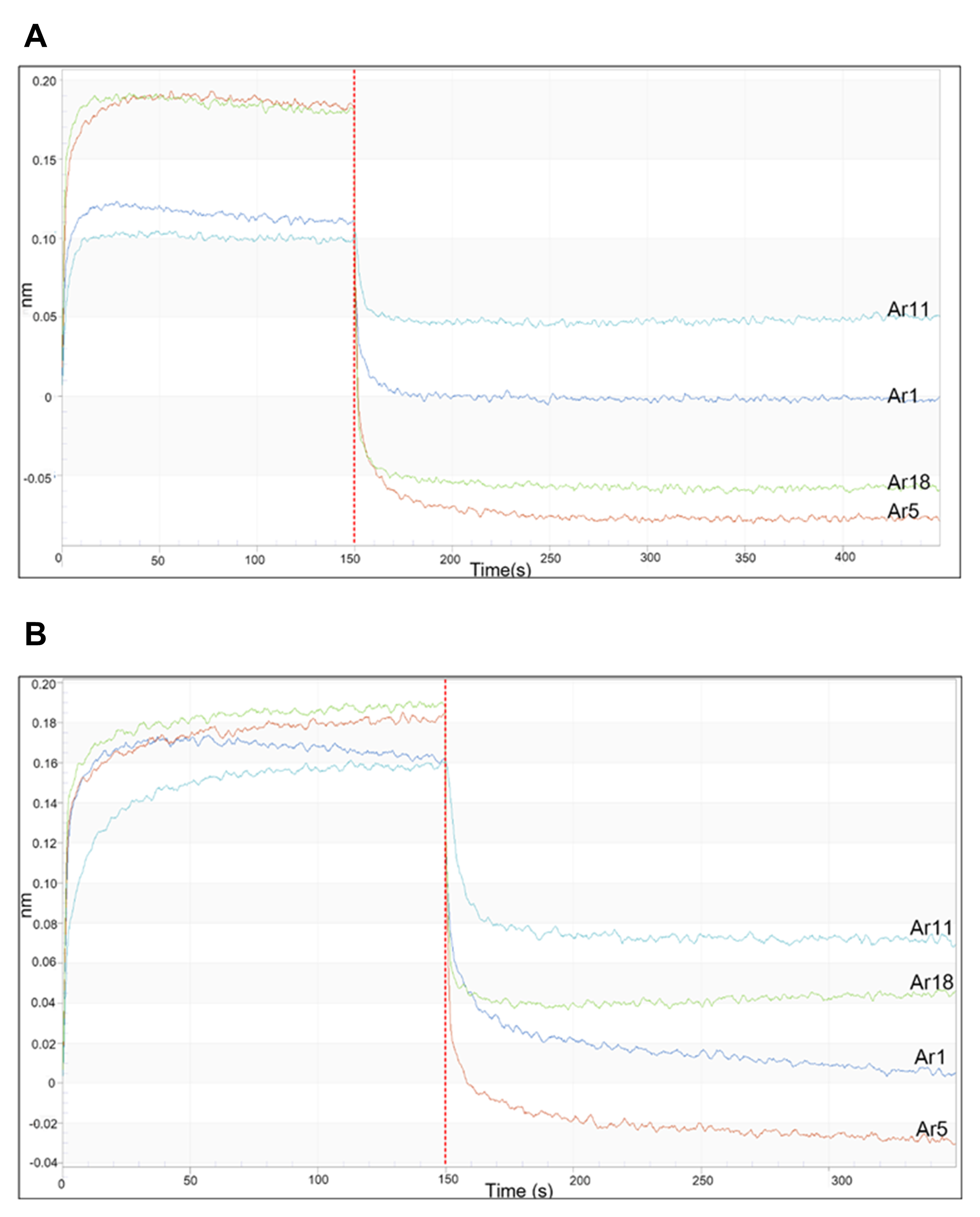

Supplement: S5 Fig — (A) Binding of Ar1, Ar5, Ar11, and Ar18 with AcrBF615A, F617A, R620A protein (B) Binding of A1, Ar5, Ar11 and Ar18 with AcrBF178A protein. (TIF) [file ppat.1012121.s017.tif]

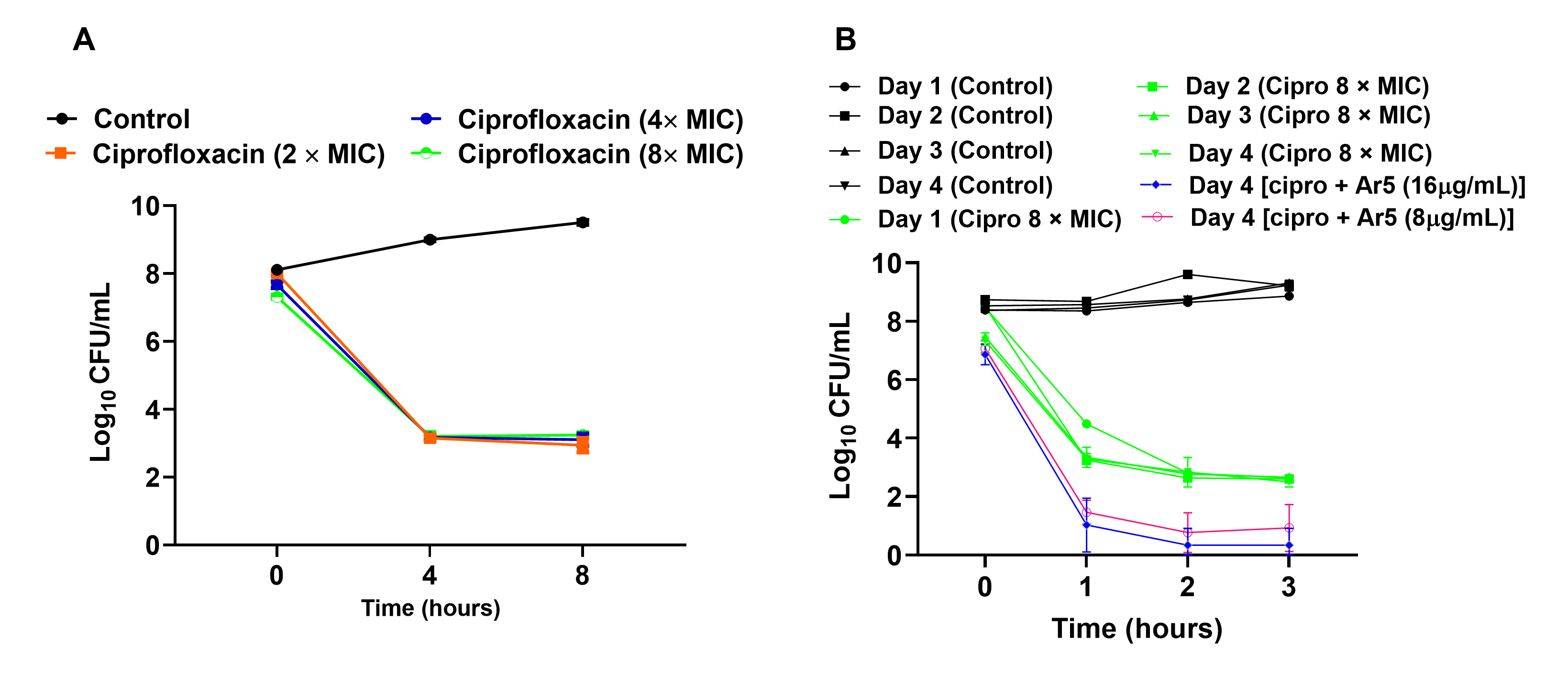

Supplement: S6 Fig — Persister-killing assay (A) Biphasic killing curves represent the persister formation frequency of P. aeruginosa ATCC 27853 under ciprofloxacin treatment at different concentrations (2, 4, and 8 × MIC). (B) Biphasic killing curves represent the persister formation frequency of P. aeruginosa ATCC 27853 under ciprofloxacin (8 × MIC) treatment in the absence or presence of Ar5 at sub-inhibitory concentrations (8 μg/mL and 16 μg/mL). (TIF) [file ppat.1012121.s018.tif]

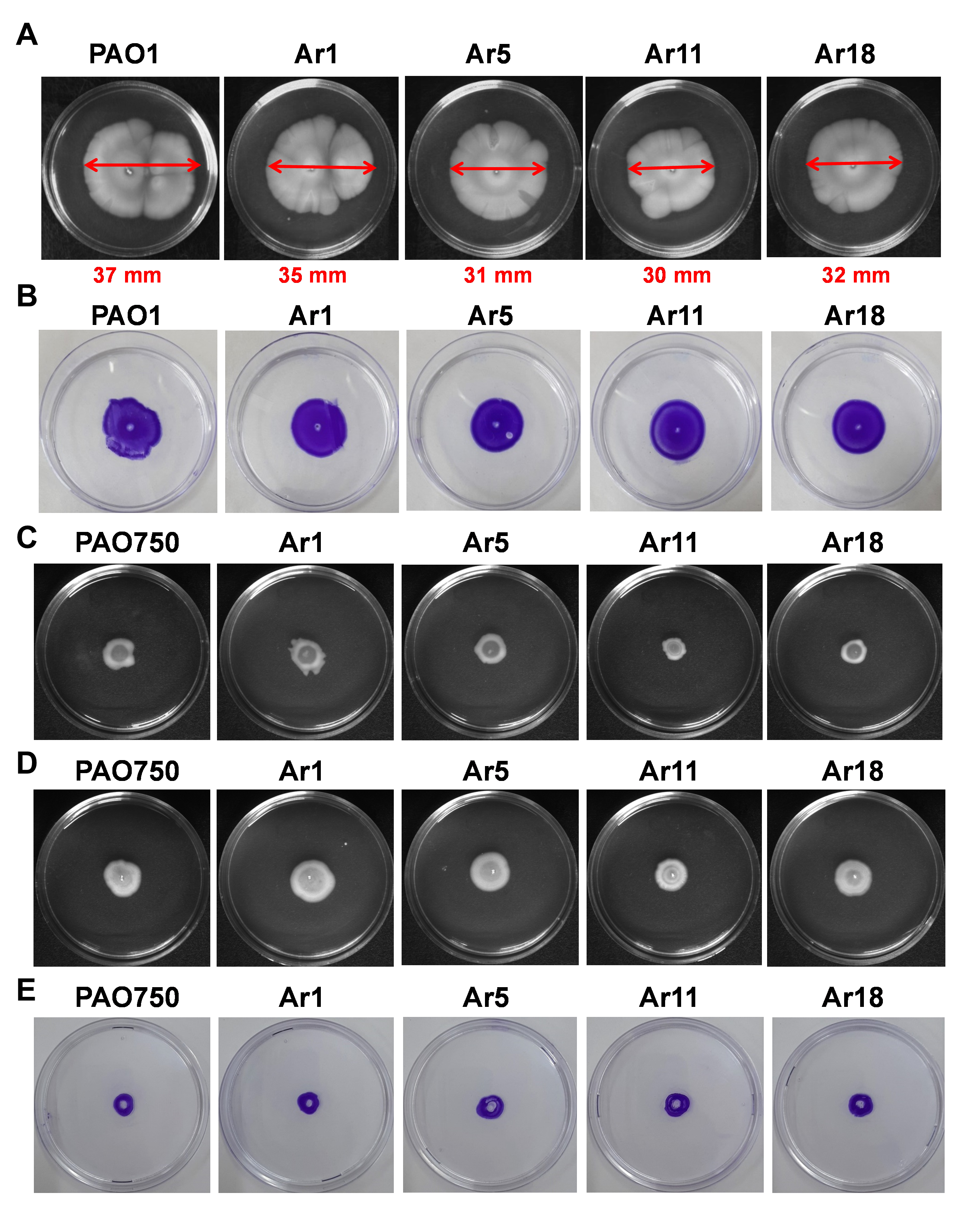

Supplement: S7 Fig — P. aeruginosa PAO1 (A) swarming motility (B) twitching motility in the absence (control) and presence of EPIs (Ar1, Ar5, Ar11, and Ar18) at sub-inhibitory concentrations (1/4 × MIC; 16 μg/mL). P. aeruginosa PAO750 (C) swimming motility (D) swarming motility (E) twitching motility in the absence and presence of EPIs (Ar1, Ar5, Ar11, and Ar18) at sub-inhibitory concentrations (1/4 × MIC; 16 μg/mL). For visualization of twitching, the attached cells were stained with crystal violet (1% w/v) for 5 min after carefully removing the agar, followed by washing to remove excess stain. (TIF) [file ppat.1012121.s019.tif]

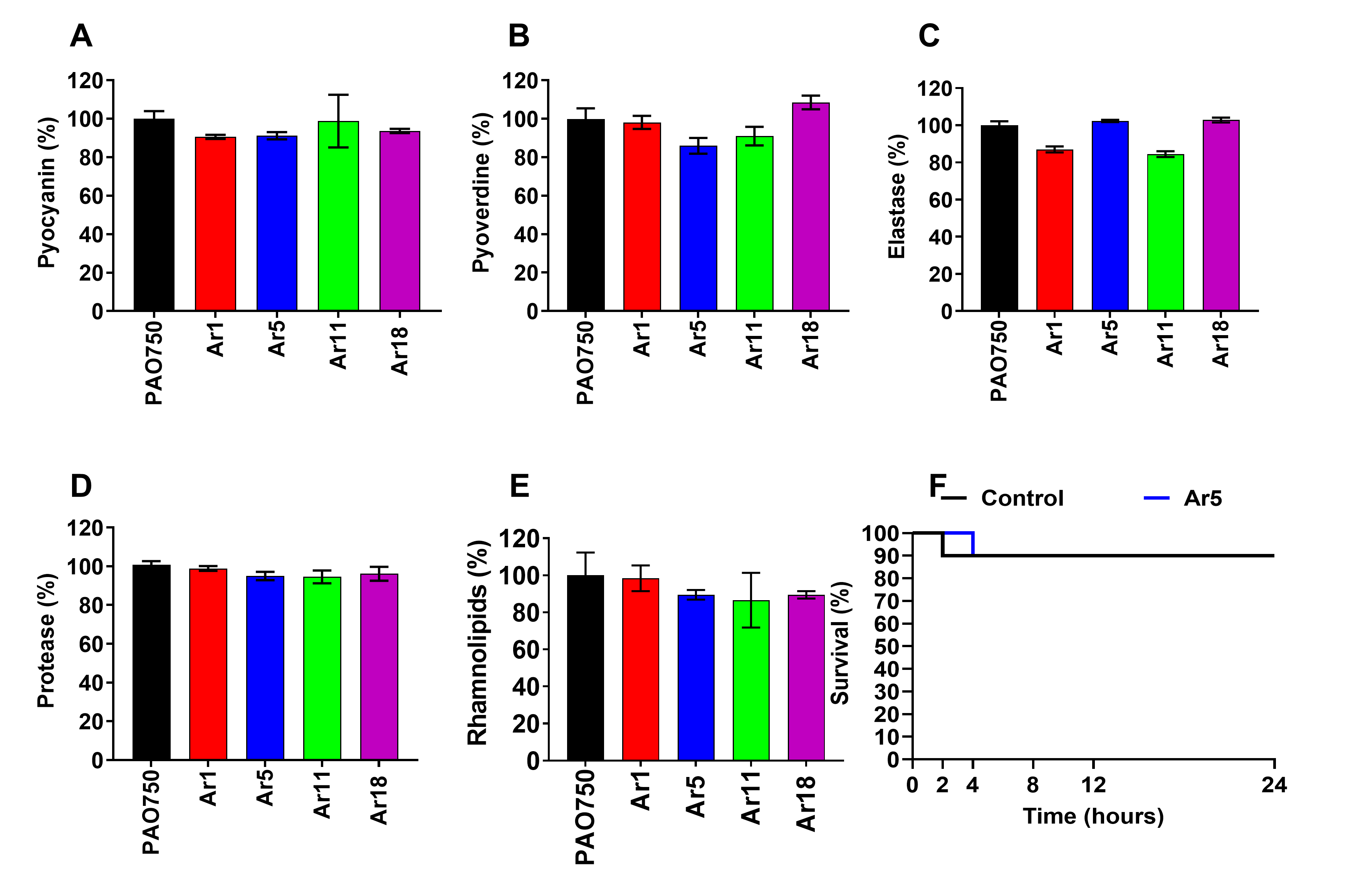

Supplement: S8 Fig — Effect of EPIs (Ar1, Ar5, Ar11, and Ar18) on the production of extracellular virulence factors; pyocyanin (A), pyoverdine (B), elastase (C), protease (D), and rhamnolipids (E) levels in the culture supernatants of P. aeruginosa PAO750 in the presence of sub-inhibitory concentrations of EPIs (16 μg/mL). (F) Effect of Ar5 on P. aeruginosa PAO750 virulence toward C. elegans. (TIF) [file ppat.1012121.s020.tif]

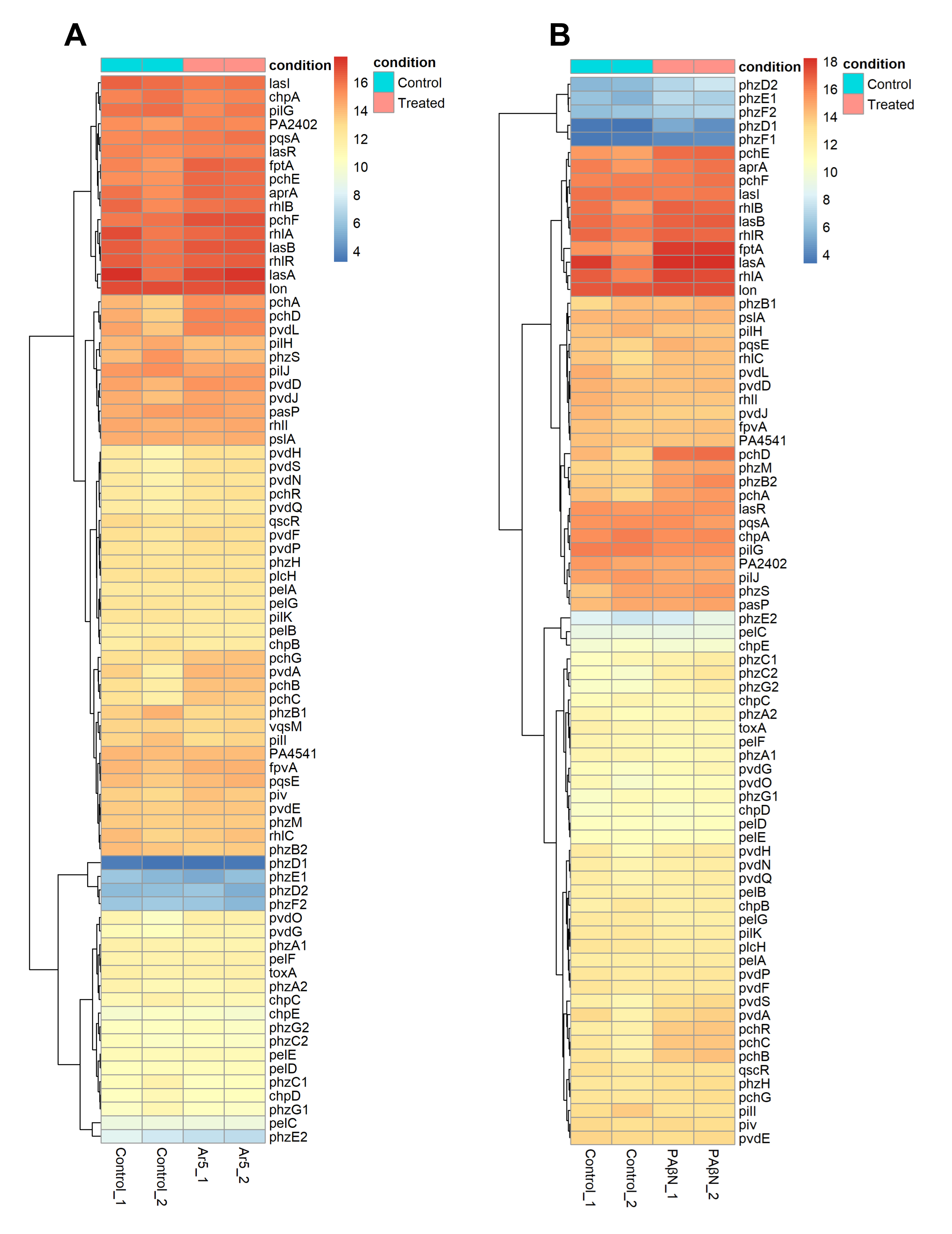

Supplement: S9 Fig — Effect of (A) Ar5 and (B) PAβN on virulence genes expression. The interactive heat map representing a differential expression of P. aeruginosa PAO1 virulence genes on a gradient scale in response to treatment with Ar5 (16 μg/mL) and PAβN (16 μg/mL) for 18 h. (TIF) [file ppat.1012121.s021.tif]

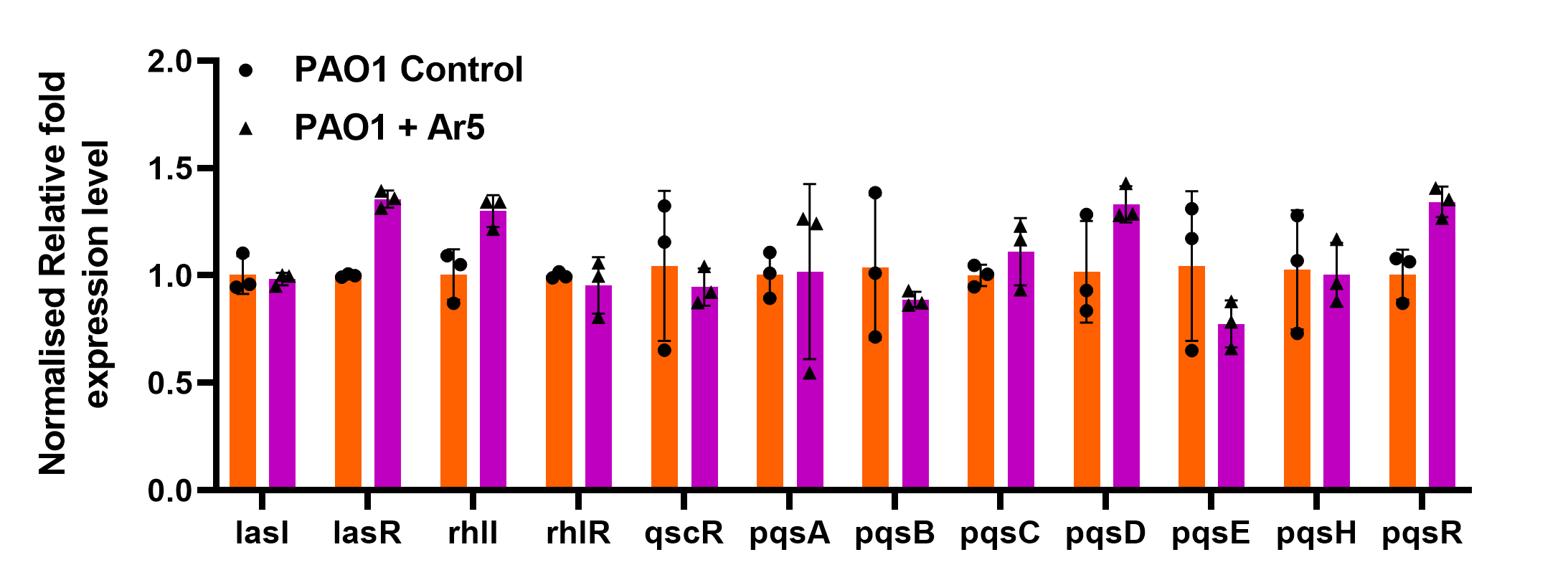

Supplement: S10 Fig — The relative fold expression levels were calculated using the 2-ΔΔCT method. The average of triplicates ± SD is shown. (TIF) [file ppat.1012121.s022.tif]

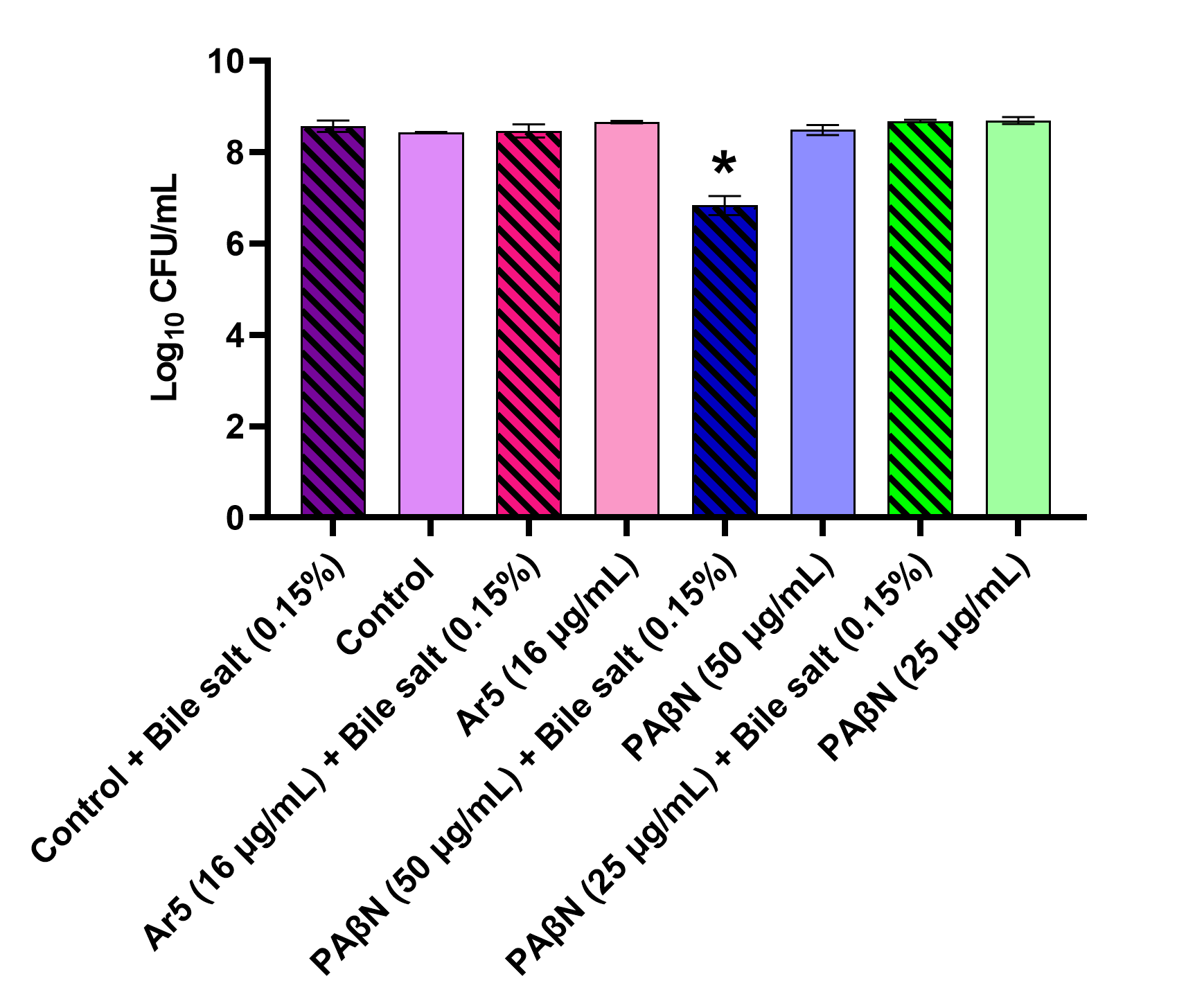

Supplement: S11 Fig — The average of triplicates ± SD is shown. Results were considered significant when *p<0.05 and highly significant when **p<0.01 and ***p<0.001. (TIF) [file ppat.1012121.s023.tif]
