# Supplementary material for: Pyrrole-based inhibitors of RND-type efflux pumps reverse antibiotic resistance and display anti-virulence potential
Source: PLoS Pathog. 2024 Apr 9;20(4):e1012121. doi: 10.1371/journal.ppat.1012121 (PMC11003683; doi:10.1371/journal.ppat.1012121)
Supplement: S1 Supporting Information — General chemistry; S2 Method. MIC determination; S3 Method. Molecular docking studies; S4 Method. Cloning, expression, and purification of AcrB; S5 Method. Site-directed mutagenesis, expression, and purification of AcrB; S6 Method. Persister assay for P. aeruginosa; S7 Method. Biofilm eradication assa; S8 Method. Outer membrane permeabilization assay; S9 Method. Membrane depolarization assay; S10 Method. ATP determination assay; S11 Method. Membrane fluidity assay; S12 Method. Growth kinetics determination; S13 Method. Motility assays; S14 Method. RNA isolation, RNA sequencing, and qRT-PCR; S15 Method. Determination of bile salts potentiation; S16 Method. In silico absorption, distribution, metabolism, and excretion (ADME) analysis. (PDF) [file ppat.1012121.s024.pdf]

Supporting information

Pyrrole-based inhibitors of RND-type efflux pumps reverse antibiotic resistance and display anti-virulence potential

Nisha Mahey[1, 2], Rushikesh Tambat[1,5#], Ritu Kalia[3], Rajnita Ingavale[3], Akriti kodesia[2, 4], Nishtha Chandal[1,2], Srajan Kapoor[4], Dipesh Kumar Verma[4,6#], Krishan Gopal Thakur[4], Sanjay Jachak[3*], Hemraj Nandanwar[1, 2*]

[1]Clinical Microbiology & Antimicrobial Research Laboratory, CSIR-Institute of Microbial Technology, Sector 39-A, Chandigarh, India

[2]Academy of Scientific & Innovative Research (AcSIR), Ghaziabad, Uttar Pradesh, India

[3]Department of Natural Products, National Institute of Pharmaceutical Education and Research, Mohali, India

[4]Structural Biology Laboratory, CSIR-Institute of Microbial Technology, Chandigarh, India

[5]Department of Chemistry and Biochemistry, University of Oklahoma, Norman, Oklahoma, United States of America

[6]Department of Life Sciences and Biotechnology, Chhatrapati Shahu ji Maharaj University, Kanpur, India

[#]Current affiliation

***Address for correspondence**

Dr. Hemraj Nandanwar, Chief Scientist, CSIR-Institute of Microbial Technology, Sector 39-A, Chandigarh-160036, India

Email ID: hemraj@imtech.res.in

Telephone: +91-172-2880338   Fax: +91-172-2690585/2690632

#Address for co-correspondence

Dr. Sanjay Jachak, Professor, Department of Natural Products, National Institute of Pharmaceutical Education & Research, Mohali-160062, Punjab

Email ID: sjachak11@gmail.com

Telephone: 91-172-2292037

Short title: Pyrrole-based RND-type efflux inhibitors

Table of content

S1 Method. General chemistry

S2 Method. MIC determination

S3 Method. Molecular docking studies

S4 Method. Cloning, expression, and purification of AcrB

S5 Method. Site-directed mutagenesis, expression, and purification of AcrB

S6 Method. Persister assay for *P. aeruginosa*

S7 Method. Biofilm eradication assay

S8 Method. Outer membrane permeabilization assay

S9 Method. Membrane depolarization assay

S10 Method. ATP determination assay

S11 Method. Membrane fluidity assay

S12 Method. Growth kinetics determination

S13 Method. Motility assays

S14 Method. RNA isolation, RNA sequencing, and qRT-PCR

S15 Method. Determination of bile salts potentiation

S16 Method. *In silico* absorption, distribution, metabolism, and excretion (ADME) analysis

Supplementary methods

S1 Method. *General chemistry*

All Chemicals and reagents used for the synthesis were procured from Sigma Aldrich (St. Louis, MO, USA), Alfa-aesar (Johnson Matthey Company, Ward Hill, MA, USA), and S.D. Fine chemicals Ltd.(India). Silica gel (#60-120 and #100-200), and neutral alumina were used for column chromatography (CC), and thin-layer chromatography (TLC) precoated plates were purchased from Merck (Germany). The organic solvents used for column chromatography (CC) were obtained from Finar and Rankem (India). ^{1}H and ^{13}C NMR spectra were recorded on Bruker Avance DPX 400 spectrometer (Brukers, Germany) at 400 MHz and 100 MHz, respectively, and on JNM-ECA 500 series NMR Jeol spectrometer (JEOL, Japan) at 500 MHz and 125 MHz. NMR spectra were recorded in $CDCl_3$, MeOH-d_4 and/or DMSO-d_6 at room temperature. Chemical shifts values (δ) are expressed in ppm relative to internal standard tetramethylsilane (TMS). Coupling constants (J) are given in hertz (Hz). NMR data are reported in the following order: chemical shift, multiplicity (s = singlet, d = doublet, t = triplet, dd = doublet of doublets, dt = doublet of triplets, m = multiplet, br = broad), and combinations of these coupling constants and integration. The mass spectra were recorded on Agilent 6200 series TOF/6500 series Q-TOF 10.1. The purity of final compounds was determined using the Waters ACQUITY UPLC H-Class system, equipped with a binary pump, autosampler, photodiode array detector (PDA), and Empower™ 3 software. CEM microwave synthesizer (DISCOVER SYSTEM Model no.908010) was utilized for performing coupling reactions.

Scheme: 1 Synthetic scheme for 4-bromo-1H-2-ethyl carboxylate derivatives [a]

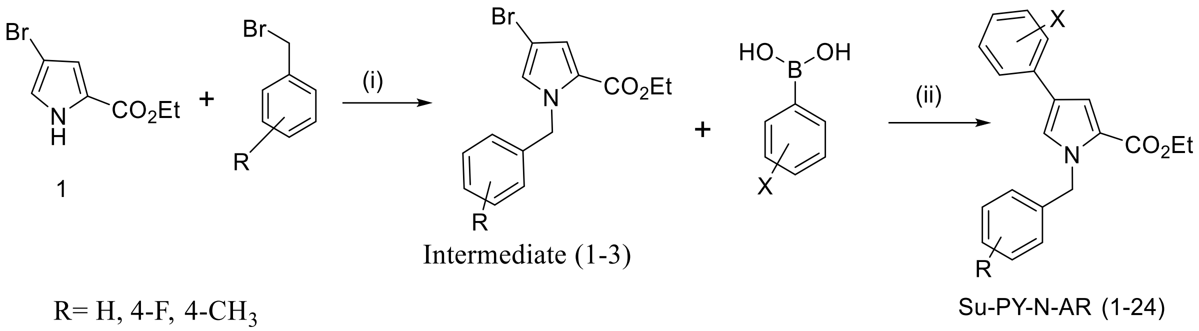

R= H, 4-F, 4-CH_3

X= 4-F, 4-CHO, 4-OCH_3, 4-OH, 4-CN, 4-COOH, 3-$NHCOCH_3$, 4-$COCH_3$, 3-NH_2, 4-CF_3, 2,4-di-fluoro, 5-fluoropyridine, 3-CH_2OH, 3-OH, 4-CH_3

[a] (i) Aq.NaOH,TBAB,DCM, 0 ^{0}C -18 ^{0}C, 15 hrs, (ii) microwave assisted reaction; [Pd(Ph_3P)$_4$], Cs_2CO_3, DMF:H_2O(2:1), 130 ^{0}C,125 W, 100 Psi, 30-45 min.

General procedure for the synthesis of 4-bromo-1H-2-ethyl carboxylate scaffold derivatives

To the solution of 4-bromo-1H-2-ethyl carboxylate (RP1) (1eq.) in dichloromethane (DCM) maintained at 0°C, aqueous solution of 50% w/v NaOH and 40 % w/v Tetrabutylammonium bromide (TBAB) was added. Substituted/ unsubstituted benzyl bromide (1.1eq.) was then added dropwise to the above solution, maintained at 0°C. The temperature was increased upto 18°C over 1h. The reaction was completed in 15 h. The reaction mixture was acidified with 3M HCl to pH 1 and then extracted with ethyl acetate: water. The combined organic phase was dried over anhydrous Na_2SO_4 and filtered. The solvent was removed under reduced pressure to obtain a crude product. The purification was done using silica gel (#100-200) column chromatography. The column was eluted with 2-5 % ethyl acetate in hexane to give ethyl 4-bromo-1-(4-fluorobenzyl/4-methylbenzyl/benzyl)-1H-pyrrole-2-carboxylate (intermediate 1/2/3) Step (ii) was done using Suzuki-Miyaura cross-coupling reaction of intermediate 1/2/3 (1 eq.) with substituted aryl boronic acids (2.5 eq.), cesium carbonate (4 eq.) as base and Tetrakis (0.1eq.) as a catalyst in a microwave reactor (Temp-130°C, power-125W, time- 30-45 min.). DMF:

H_2O (2:1) was used as a solvent. The crude reaction mixture was extracted with ethyl acetate: water. The combined organic phase was dried over anhydrous Na_2SO_4 and filtered. Final purification was done using silica gel (#100-200) column chromatography. The column was eluted with 15-35% ethyl acetate in hexane to give desired ethyl 1, 4 substituted, 1H-pyrrole-2-carboxylate derivatives [1-3].

Intermediate 1

4-Bromo-1-(benzyl)-pyrrole-2-ethylcarboxylate

Yield (80 %); yellow oily liquid; ESI-HRMS m/z; calcd for $C_{14}H_{15}BrNO_2$ $[M+H]^+$= 308.0286; found 308.0280

Intermediate 2

4-Bromo-1-(4-fluoro benzyl)-pyrrole-2-ethylcarboxylate

Yield (80 %); yellow oily liquid; ESI-HRMS m/z; calcd for $C_{14}H_{13}BrFNO_2$ $[M+H]^+$= 326.0192; found 326.0196

Intermediate 3

4-Bromo-1-(4-methyl benzyl)-pyrrole-2-ethylcarboxylate

Yield (75 %); brown yellow oily liquid; ESI-HRMS m/z; calcd for $C_{14}H_{13}BrFNO_2$ $[M+H]^+$= 326.0192; found 326.0196

1. SU-Py-N-Ar-1

Ethyl 1-(4-fluorobenzyl)-4-(4-flurophenyl)-1H-pyrrole-2-carboxylate

Yield 26 %; white solid; ^1H NMR (400 MHz, $CDCl_3$) δ ppm: 7.46-7.42 (m, 2H), 7.22 (d, *J*= 2.0 Hz, 1H), 7.15-7.11 (m, 2H), 7.08 (d, *J*= 2.0 Hz, 1H), 7.05-6.6 (m, 4H), 5.5 (s, 2H), 4.25 (q *J*= 7.12 Hz, 2H), 1.32 (t, *J*= 7.12 Hz, 3H) ^{13}C NMR (100 MHz, $CDCl_3$) δ ppm 163.4, 162.8, 161.0,160.4, 133.7, 133.7, 130.5, 130.4, 128.7, 128.7, 126.6, 126.6, 125.0, 123.8, 123.1,115.7, 115.5, 115.4, 60.14, 51.5, 14.4

ESI-HRMS m/z; calcd for $C_{20}H_{16}F_2NO_2$ $[M+H]^+$ 342.1306; found 342.1300

2. SU-Py-N-Ar-2

Ethyl 1-(4-fluorobenzyl)-4-(4-formylphenyl)-1H-pyrrole-2-carboxylate

Yield 26 %; white solid; ^{1}H NMR (400 MHz, DMSO-d_6) δ ppm: 9.9 (s, 1H), 8.05-8.02(m, 1H), 7.89-7.84 (m, 4H), 7.46 (d, *J*=2.0 Hz,1H), 7.26-7.23(m, 2H), 7.19-7.15 (m, 2H), 5.56 (s, 1H), 4.21 (q, *J*=8 Hz, 2H), 1.26-1.22(m, 3H) ^{13}C NMR: 192.7, 160.7, 140.5, 135.0, 134.9, 134.3, 130.8, 130.7, 129.4, 129.3, 128.9, 128.4, 125.4, 123.2, 122.7, 115.9, 115.9, 115.7, 60.3, 51.3, 14.7

ESI-HRMS m/z; calcd for $C_{21}H_{19}FNO_3$ $[M+H]^+$ 352.1349; found 352.1347

3. Su-Py-N-Ar-3

Ethyl 1-(4-fluorobenzyl)-4-(4-methoxyphenyl)-1H-pyrrole-2-carboxylate

Yield 92 %; white solid;^{1}H NMR (400 MHz, CD_3OD) δ ppm: 7.44 (d, *J*=8.4 Hz, 2H), 7.35(m, 1H), 7.2 (d, *J*=2.0 Hz) 7.15 (m, 2H), 7.0 (t, *J*=8 Hz, 2H), 6.88 (d, *J*= 8.4 Hz, 2H), 5.52 (s, 2H), 4.2 (q, *J*=7.2Hz, 2H), 3.77(s, 3H), 1.28 (t, *J*=7.1 Hz, 3H) ^{13}C NMR (100 MHz,CD_3OD) δ ppm: 164.6, 162.4, 162.2, 159.7, 136.1, 136.0, 129.7, 129.6, 128.3, 127.1, 126.7, 125.8, 123.7, 116.18, 115.9, 115.1, 60.9, 55.6, 52.2, 14.6

ESI-HRMS *m/z*; calcd for $C_{21}H_{21}FNO_3$ $[M+H]^+$ 354.1505; found 354.1498

4. Su-Py-N-Ar-4

Ethyl 1-(4-fluorobenzyl)-4-(4-hydroxyphenyl)-1H-pyrrole-2-carboxylate

Yield 43 %; white solid;^{1}H NMR (400 MHz, $CDCl_3$) δ ppm: 7.39 (d, *J*=8.3 Hz, 2H), 7.24(m, 1H), 7.15 (m, 2H), 7.09 (s, 1H), 7.01 (m, 2H), 6.84 (d, *J*= 8.4 Hz, 2H), 5.55 (s, 2H), 5.08 (s, 1H), 4.28 (q, *J*=8 Hz, 2H), 1.34 (t, *J*=7.1 Hz, 3H) ^{13}C NMR (100 MHz, $CDCl_3$) δ ppm: 163.4, 161.2, 160.9, 154.3, 133.9, 133.8, 128.7, 128.6, 127.1, 126.5, 126.5, 124.8, 124.4, 122.8, 115.7, 115.5, 115.3, 60.1, 51.5, 14.4

ESI-MS *m/z*; calcd for $C_{20}H_{19}FNO_3$ $[M+H]^+$ 340.1349; found 340.1344

5. Su-Py N-Ar-5

Ethyl 1-benzyl-4-(4-fluorophenyl)-1H-pyrrole-2-carboxylate

Yield 50 %; white solid; ^{1}H NMR (400 MHz, $CDCl_3$) δ ppm 7.48- 7.47 (m, 2H), 7.36- 7.27 (m, 4H), 7.18 (d, J= 7.1 Hz, 2H), 7.13 (s, 1H), 7. 05(t, J= 8.52 Hz, 2 H), 5.61 (s, 2H), 4.28(q, J= 7.04Hz, 2H), 1.35 (t, J= 7.04, 3H) ^{13}C NMR: 159.9, 159.2, 136.9, 129.5, 127.6, 127.6, 126.5, 125.9, 125.9, 125.6, 125.5, 124.1, 122.6, 122.2, 114.6, 114.4, 114.2, 59.0, 51.2, 13.3; ESI-HRMS *m/z*; calcd for $C_{20}H_{19}FNO_2$ $[M+H]^+$324.1400; found 324.1394

6. Su-Py-N-Ar-6

Ethyl 1-benzyl-4-(4-methoxyphenyl)-1H-pyrrole-2-carboxylate

Yield 71 %; white solid; ^{1}H NMR (400 MHz, $CDCl_3$) δ ppm: 7.43 (m, 2H), 7.31 (m, 2H), 7.25 (m, H), 7.23 (d, J= 2 Hz, 1H) 7.15 (d, J= 7.04 Hz, 2H), 7.08 (d, J= 2.0 Hz), 6.89(m, ,2H), 5.58 (s, 2H), 4.25 (q, J= 7.16 Hz, 2H), 3.81 (s, 3H), 1.32 (t, J=7.12 Hz, 3H). ^{13}C NMR (100 MHz, $CDCl_3$) δ ppm: 161.1, 158.2, 138.2, 128.7 (2C), 127.5, 127.2. 126.9 (2C), 126.3 (2C), 124.9, 124.3, 122.9, 115.1, 114.2 (2C), 59.9, 55.3, 52.2, 14.4;ESI-HRMS *m/z*; calcd for $C_{21}H_{22}NO_3$ $[M+H]^+$337.1633, found: 337.2348

7. Su-Py-N-Ar-7

Ethyl 1-benzyl-4-(4-hydroxyphenyl)-1H-pyrrole-2-carboxylate

Yield 79%; white solid; ^{1}H NMR (400 MHz,CD_3OD) δ ppm: 7.27 (m, 2H), 7.24-7.16 (m, 3H), 7.13 (d, J = 7.16 Hz, 1H), 7.1 (d, J = 1.96 Hz, 1H), 7.02 (d, J = 7.2 Hz, 2H), 6.68 (d, J = 8.6 Hz, 2H), 5.47 (s, 2H), 4.11 (q, J = 7.12 Hz, 2H), 1.85 (t, J = 7.12Hz, 3H). ^{13}C NMR (100 MHz,CD_3OD) δ ppm: 161.2, 155.6, 138.8, 128.1 (2C), 126.9, 126.3 (2C), 126.0, 125.8 (2C), 125.4, 124.7, 122.4, 115.1 (2C), 114.8, 59.6, 51.6, 13.3; ESI-HRMS *m/z*; calcd for $C_{20}H_{20}NO_3$ $[M+H]^+$ 322.1443, found: 322.1437

8. Su-Py-N-Ar-8

Ethyl 1-benzyl-4-(4-cyanophenyl)-1H-pyrrole-2-carboxylate

Yield 71%; white solid; ^{1}H NMR (400 MHz, $CDCl_3$) δ ppm: 7.61-7.55 (m, 4H), 7.35-7.32 (m, 3H), 7.29-7.27 (m, 1H), 7.23 (d, J= 2.0 Hz, 1H), 7.17-7.15 (m, 2H), 5.60 (s, 2H), 4.27 (q, J= 7.12 Hz, 2H), 1.33 (t, J= 7.16 Hz,

3H). ^{13}C NMR (100 MHz, $CDCl_3$) δ ppm: 160.8, 139.0, 137.4, 132.6 (2C), 128.8 (2C), 127.7, 127.0 (2C), 126.1, 125.3 (2C), 123.9, 122.6, 119.2, 115.4, 109.1, 60.3, 52.4, 14.3; ESI-HRMS *m/z*; calcd for $C_{21}H_{19}N_2O_2$ $[M+H]^+$ 331.1447, found 331.1441

9. Su-Py-N-Ar-9

4-(1-benzyl-5-(ethoxycarbonyl)-1H-pyrrol-3-yl)benzoic acid

Yield 70 %; white solid;^{1}H NMR (400 MHz, $CDCl_3$) δ ppm ; 8.10 (d, *J*= 8.36 Hz, 2H), 7.61(d, *J*=8.37 Hz, 2H), 7.39(d, *J*=1.96 Hz,1H), 7.37-7.35 (m, 1H), 7.33-7.30 (m, 2H), 7.19 (d, *J*=7.02 Hz, 2H), 5.635 (s, 2H), 4.30 (q, *J*=7.12 Hz, 2H), 1.36(t, *J*=7.12 Hz, 3H). ^{13}C NMR (100 MHz, $CDCl_3$) δ ppm; 171.4 (2C), 160.9, 139.8, 137.6, 130.8, 128.7 (2C), 127.7, 126.9, 126.6, 126.2, 124.7, 123.7, 123.2, 115.6, 60.2, 52.4, 14.3; ESI-HRMS *m/z*; calcd for $C_{21}H_{19}NNaO_4$ $[M+Na]^+$ 372.1212; found 372.1205

10. Su-Py N-Ar-10

Ethyl 4-(3-acetamidophenyl)-1-benzyl-1H-pyrrole-2-carboxylate

Yield 58 %; white solid; ^{1}H NMR (400 MHz,DMSO-d_6)) δ ppm 9.93 (s, 1H), 7.82(s, 1H), 7.72(d, *J*= 2Hz, 1H),7.41-7.39(m, 1H), 7.35-7.31 (m, 2H),7.27-7.24(m, 3H), 7.19(d, *J*= 2.0 Hz,1 H), 7.16(m, 2H), 5.59(s, 2H), 4.18(q, *J*= 7.2 Hz, 2H), 2.05 (s, 3H), 1.24(t, *J*= 7.08, 3H). ^{13}C NMR (100 MHz, DMSO-d_6) δ ppm 168.8, 160.6, 140.3,139.1, 134.7, 129.6, 128.9,128.9, 127.8, 127.3, 127.1,127.1, 123.9, 122.7, 119.9, 117.3,115.7, 115.0, 60.1, 51.8, 24.5, 14.7. ESI-HRMS *m/z*; calcd for $C_{22}H_{23}N_2O_3$ $[M+H]^+$363.1709; found 363.1707

11. Su-Py-N-Ar-11

Ethyl 4-(3-acetamidophenyl)-1-(4-fluorobenzyl)-1H-pyrrole-2-carboxylate

Yield 55%; white solid;^{1}H NMR (400 MHz, DMSO-d_6) δ ppm : 9.94 (s, 1H), 7.82 (s, 1H), 7.74 (d, J= 1.2Hz, 1H), 7.4-7.38 (m, 1H), 7.27- 7.20 (m, 3H), 7.19-7.14 (m, 3H), 5.56 (s, 2H), 4.19 (q, *J*= 7.08 Hz, 2H), 2.05 (s, 3H), 1.25 (t, *J*= 7.12 Hz, 3H).^{13}C NMR (100 MHz, DMSO-d_6) δ ppm; 168.8, 163.1, 160.6, 140.26, 160.6, 140.3,

135.3, 135.2, 134.7, 129.6, 129.4, 129.3, 127.2, 123.9, 122.5, 119.9, 117.3, 115.9, 115.7, 115.1, 60.2, 51.0, 24.5, 14.7 ESI-HRMS *m/z*; calcd for $C_{22}H_{22}FN_2O_3$ $[M+H]^+$381.1614, found381.1612

12. Su-Py-N-Ar-12

Ethyl 4-(4-acetylphenyl)-1-benzyl-1H-pyrrole-2-carboxylate

Yield 68%; white solid; ^{1}H NMR (400 MHz, $CDCl_3$) δ ppm; 7.93(d, *J*=3.62 Hz,2H), 7.57(d, *J*=8.36Hz, 2H), 7.35(d,*J*=2.00Hz, 1H), 7.30(m, 2H), 7.25(m, 1H), 7.16(d, *J*=7.08Hz,2H), 5.60(s,2H), 4.27(q, *J*=7.12Hz, 2H), 2.59(s, 3H), 1.33(t, *J*=7.12Hz, 3H).^{13}C NMR (100 MHz, $CDCl_3$) δ ppm; 197.5, 160.8, 139.1, 137.6, 134.7,129.0, 128.7, 127.6, 126.9, 126.2, 124.7, 123.7, 123.2, 115.5, 60.2, 52.4, 26.5, 14.3 ;ESI-HRMS *m/z*; calcd for $C_{22}H_{22}NO_3$ $[M+H]^+$ 348.1600; found 348.1597

13. Su-Py-N-Ar-13

Ethyl 4-(3-aminophenyl)-1-(4-fluorobenzyl)-1H-pyrrole-2-carboxylate

Yield 65%; white solid;^{1}H NMR (400 MHz, $CDCl_3$) δ ppm ;7.28(s, 1H), 7.18- 7.14 (m, 4H),7.04- 6.99 (m, 2H), 6.95-6.93(m, 1H), 6.86 (t, J= 19.6 Hz, 1H), 6.59-6.56 (m, 1H), 5.57 (s, 2H), 4.28 (q, *J*=7.16 Hz,2H), 3.69 (s,2H), 1.35(t, *J*=7.12 Hz,3H).^{13}C NMR (100 MHz, $CDCl_3$) δ ppm: 161.1, 146.8, 135.3, 133.8, 133.8, 129.7, 128.7, 128.6, 125.4, 124.7, 122.8, 115.7, 115.7, 115.6, 115.4, 113.2, 111.8, 60.1, 51.5, 14.4 ESI-HRMS *m/z*; calcd for $C_{20}H_{20}FN_2O_2$ $[M+H]^+$ 339.1509; found339.1508

14. Su-Py-N-Ar-14

Ethyl 4-(3-acetylphenyl)-1-(4-fluorobenzyl)-1H-pyrrole-2-carboxylate

Yield 50%; white solid; ^{1}H NMR (400 MHz, $CDCl_3$) δ ppm ; 7.96 (d, *J*= 8.24 Hz, 2H), 7.59 (d, *J*=8.24 Hz, 2H), 7.37 (d, *J*= 1.60 Hz, 1H), 7.28-7.27 (m, 1H), 7.19- 7.16 (m, 2H), 7.03 (t, *J*= 8.60 Hz, 2H), 5.58 (s, 2H), 4.30 (q, *J*= 7.12 Hz, 2H),2.62(s, 3H), 1.36 (t, *J*= 7.12 Hz, 3H). ^{13}C NMR (100 MHz, $CDCl_3$) δ ppm; 197.6, 160.9, 139.1, 134.8, 133.4, 129.1 (2C), 128.8, 128.7, 126.0, 124.8 (2C), 123.6, 123.4, 115.8, 115.7, 115.6, 60.3, 51.7, 26.6, 14.4;ESI-HRMS *m/z*; calcd for $C_{22}H_{21}NFO_3$ $[M+H]^+$ 366.1505; found 366.1501

15. Su-Py-N-Ar-15

4-(5-(ethoxycarbonyl)-1-(4-fluorobenzyl)-1H-pyrrol-3-yl)benzoic acid

Yield 65%; white solid;^{1}H NMR (400 MHz, $CDCl_3$) δ ppm; 8.10 (d, *J*= 8.13 Hz, 2H), 7.61 (d, *J*=8.11 Hz, 2H), 7.38 (s, 1H),7.18 (m, 2H), 7.03 (t, *J*= 8.53 Hz, 2H), 5.59 (s, 2H), 4.30 (q, *J*= 7.09 Hz, 2H), 1.37 (t, *J*=7.08 Hz, 3H).^{13}C NMR (100 MHz, $CDCl_3$) δ ppm; 171.6, 163.5, 161.1, 160.9, 139.7, 133.4, 133.4, 130.9, 128.8, 128.7, 126.7, 126.1, 124.8, 123.6, 123.4, 115.8, 115.7, 115.6, 60.3, 51.7, 14.4; ESI-HRMS *m/z*; calcd for $C_{21}H_{18}FNNaO_4$ $[M+Na]^+$ 390.1118;found 390.1110.

16. Su-Py-N-Ar-16

Ethyl 1-(4-fluorobenzyl)-4-(4-(trifluoromethyl)phenyl)-1H-pyrrole-2-carboxylate

Yield 72%; white solid;^{1}H NMR (400 MHz, $CDCl_3$) δ ppm; 7.60 (s, 4H), 7.34 (d, *J*= 1.80 Hz, 2H), 7.22(d, *J*= 1.68 Hz, 1H), 7.18 (m, 2H), 7.03 (t, *J*= 8.62 Hz, 2H), 5.58 (s, 2H), 4.30 (q, *J*= 7.10 Hz, 2H), 1.36 (t, *J*= 7.10 Hz, 3H). ^{13}C NMR (100 MHz, $CDCl_3$) δ ppm; 163.5, 160.9, 137.9, 133.5, 133.4, 128.8, 128.7, 128.3, 127.9, 125.7, 125.7, 127.5, 125.7, 125.1, 123.6, 123.2, 115.8, 115.6, 115.5, 60.2, 51.7, 14.3 ESI-HRMS *m/z*; calcd for $C_{21}H_{17}NF_4O_2$ $[M+H]^+$ 392.1274; found 392.1268

17. Su-Py-N-Ar-17

Ethyl 4-(4-fluorophenyl)-1H-pyrrole-2-carboxylate

Yield 25%;white solid;^{1}H NMR(400MHz,CDCl3)δppm;9.19(s,1H),7.49(m,2H),7.18(d,*J*=13.19Hz,2H), 7.07(t,*J*=8.56Hz,2H),4.37(q,*J*=7.11Hz,2H),1.40(t,*J*=7.12Hz,3H).^{13}CNMR(100 MHz,CDCl3)δppm;161.0,126.8,126.8,126.0,123.9,119.0,115.7,115.5,112.3,60.6,14.5. ESI-HRMS *m/z*; calcd for $C_{13}H_{13}NFO_2$ $[M+H]^+$ 234.0930, found 234.0923

18. Su-Py-N-Ar-18

Ethyl 4-(2,4-difluorophenyl)-1-(4-fluorobenzyl)-1H-pyrrole-2-carboxylate

Yield 76%; white solid; ^{1}H NMR (400 MHz, $CDCl_3$) δ ppm;7.52(q, *J*=8.03,1H), 7.30(m,2H), 7.17(m,2H), 7.02(t, *J*=8.57 Hz,2H), 6.88(q, *J*=7.17Hz,2H), 5.57(s,2H), 4.29(q, *J*=7.10 Hz, 2H), 1.35(t, *J*= 7.10 Hz,3H).^{13}C NMR (100 MHz, $CDCl_3$) δ ppm;161.0, 160.9, 133.7, 128.7, 128.6, 128.4, 128.3, 128.3, 128.2, 127.6, 127.5, 122.6, 117.4, 116.4, 115.7, 115.4, 111.6, 111.3, 104.6, 104.3, 104.1, 60.1, 51.6, 14.4;ESI-HRMS *m/z*; calcd for $C_{20}H_{17}NF_3O_2$ [M+H]$^+$ 360.1211;found 360.1204

19. Su-Py-N-Ar-19

Ethyl 1-(4-fluorobenzyl)-4-(5-fluoropyridin-2-yl)-1H-pyrrole-2-carboxylate

Yield 70%; white solid;^{1}H NMR (400 MHz, $CDCl_3$) δ ppm;8.60(s, 1H),8.31(d, *J*=2.44 Hz,1H), 7.47(d, *J*=9.70 Hz,1H), 7.30(d, *J*=1.37 Hz,1H), 7.19(m,3H), 7.03(t, *J*=8.55 Hz,2H), 5.58(s,2H), 4.30(q, *J*=7.10 Hz,2H), 1.36(t, *J*=7.10 Hz,3H)^{13}C NMR (100 MHz, $CDCl_3$) δ ppm;163.6, 161.1, 160.7, 158.6, 142.4, 142.4, 135.5, 135.3, 133.3, 133.2, 131.9, 131.9, 128.9, 128.8, 125.6, 123.9, 120.0, 118.9, 118.7, 115.8, 115.6, 115.5, 77.3, 77.0, 76.7, 60.4, 51.7, 14.3. ESI-HRMS *m/z*; calcd for $C_{19}H_{17}N_2F_2O_2$ [M+H]$^+$ 343.1258; found 343.1262

20. Su-Py-N-Ar-20

Ethyl 1-(4-fluorobenzyl)-4-(3-(hydroxymethyl)phenyl)-1H-pyrrole-2-carboxylate

Yield 72%; white solid; ^{1}H NMR (400 MHz, $CDCl_3$) δ ppm; 7.54 (s,1H), 7.45 (d, *J*= 7.68 Hz,1H), 7.35 (m, 2H), 7.19 (m, 4H), 7.02 (t, *J*= 8.54 Hz, 2H), 5.57 (s, 2H), 4.73 (s, 2H), 4.29 (q, *J*= 7.09 Hz, 2H), 1.36 (q, *J*= 7.10 Hz, 3H). ^{13}C NMR (100 MHz, $CDCl_3$) δ ppm;163.4, 161.0, 141.4, 134.6, 133.8, 133.7, 129.0, 128.7, 128.7, 125.4, 124.8, 124.4, 123.7, 123.1, 115.7, 115.5, 115.5, 65.4, 60.1, 51.6, 14.4.; ESI-HRMS *m/z*; calcd for $C_{21}H_{21}NFO_3$ [M+H]$^+$ 354.1505; found354.1497

21. Su-Py-N-Ar-21

Ethyl 4-(4-cyanophenyl)-1-(4-fluorobenzyl)-1H-pyrrole-2-carboxylate

Yield 68%; white solid; ^{1}H NMR (400 MHz, $CDCl_3$) δ ppm; 7.61(q, *J*= 9.22 Hz, 4H), 7.33 (d, *J*= 1.94 Hz, 1H), 7.24(d, *J*= 1.90 Hz, 1H), 7.18(m, 2H), 7.03 (t, *J*= 8.61 Hz, 2H), 5.58 (s, 2H), 4.30 (q, *J*= 7.11 Hz, 2H), 1.36 (t,

J= 7.12 Hz, 3H). ^{13}C NMR (100 MHz, $CDCl_3$) δ ppm;163.5, 160.8, 138.9, 133.3, 133.2, 132.9, 132.6, 128.9, 128.8, 127.9, 125.9, 125.3, 123.9, 122.8, 119.2, 115.8, 115.6, 115.5, 109.3, 60.4, 51.8, 14.4; ESI-HRMS *m/z*; calcd for $C_{21}H_{18}N_2FO_2$ $[M+H]^+$ 349.1352; found 349.1345

22. Su-Py-N-Ar-22

Ethyl 1-(4-fluorobenzyl)-4-(3-hydroxyphenyl)-1H-pyrrole-2-carboxylate

Yield 40%; white solid; ^1H NMR (500 MHz, $CDCl_3$) δ ppm; 7.29-7.24 (m, 1H), 7.19-7.16 (m, 1H), 7.05-6.92 (m, 6H), 6.7 (m, 1H), 6.46(s, 1H), 5.45 (m, 2H), 4.25(m, 2H), 1.3 (t, *J*=7.5 Hz, 3H).^{13}C NMR (125 MHz, $CDCl_3$) δ ppm; 163.0, 161.2, 161.1, 156.1, 135.6, 133.6, 129.9, 128.5, 128.4, 125.7, 124.2, 122.6, 117.3, 115.8, 115.5, 115.3, 113.3, 111.9, 60.3, 51.5; ESI-HRMS *m/z;* calcd for $C_{20}H_{19}FNO_3$ $[M+H]^+$ 340.1349; found 340.1342

23. Su-Py-N-Ar-23

Ethyl 4-(4-fluorophenyl)-1-(4-methylbenzyl)-1H-pyrrole-2-carboxylate

Yield 65% White solid; ^1H NMR (500 MHz, $CDCl_3$) δ ppm; 7.44 (m, 2H), 7.23-7.19 (m., 2H), 7.08-6.94 (m, 6H), 5.55 (s, 2H), 4.27 (q, *J* = 7.3 Hz, 2H), 2.34 (s, 3H), 1.33 (t, *J* = 7.2 Hz, 3H)

^{13}C NMR (126 MHz, $CDCl_3$) δ ppm; 162.3, 160.7, 138.2, 137.9, 137.5, 130.9, 128.6, 127.4, 126.5, 125.1, 123.8, 123.6, 123.2, 115.4, 114.1, 60.0, 52.2, 21.4, 14.4.ESI-HRMS *m/z*; calcd for $C_{21}H_{21}FNO_2$ $[M+H]^+$ 338.1556; found338.1551

24. Su-Py-N-Ar-24

Ethyl 1-(4-methylbenzyl)-4-(4-(trifluoromethyl)phenyl)-1H-pyrrole-2-carboxylate

Yield 70%; white solid; ^1H NMR (500 MHz, $CDCl_3$) δ ppm; 7.58 (s, 4H), 7.32 (d, *J* = 1.9 Hz, 1H), 7.23-7.19 (m, 2H), 7.09 (d, *J* = 7.6 Hz, 1H), 6.97 (m, 2H), 5.57(s, 2H), 4.29 (q, *J* = 7.0 Hz, 2H), 2.32 (s, 3H), 1.34 (t, *J* = 7.2 Hz, 3H), ^{13}C NMR (126 MHz, $CDCl_3$) δ ppm; 160.9, 138.4, 137.1, 128.6, 127.7, 125.7, 125.0, 124.1, 123.0, 115.4, 60.2, 52.3, 21.4, 14.4 ESI-HRMS *m/z*; calcd for $C_{22}H_{21}F_3NO_2$ $[M+H]^+$ 388.1524, found: 388.1519

S2 Method. MIC determination

MICs of various antibiotics and compounds were determined by broth microdilution assay following the Clinical & Laboratory Standards Institute (CLSI) guidelines [4]. The antibiotics/compounds were two-fold serially diluted into the wells of 96-well plates (along the abscissa). Further, the wells were inoculated with 5×10^5 CFU/mL of the bacterial culture and incubated for 18 h at 37 ºC in CA-MHB. The wells containing the lowest concentration without visual turbidity were considered the MIC of a compound.

S3 Method. Molecular docking studies

The AcrB and MexB dockings with compounds (Ar1, Ar5, Ar11, Ar18) and PAβN compounds were carried out using the Schrodinger software suite 2022. The molecular structures of compounds were drawn using ChemDraw software, and three-dimensional structures of Ar1, Ar5, Ar11, Ar18, and PAβN were prepared by using the Ligprep module [5]. Both AcrB and MexB structures (PDB ID: 3W9H and 3W9J) [6] were retrieved from the RCSB database and processed using a protein preparation wizard [7]. The protein preparation wizard using the OPLS3 force field was utilized to optimize the structures by adding hydrogen atoms, optimizing hydrogen bonds, removing atomic clashes, and performing other operations not part of the X-ray crystal structure refinement process [8]. The ligand binding sites in AcrB structures were searched by using SiteMap module [9]. The site with the highest score (>1.0) was selected for grid preparation and docking. A Glidegrid module was used to generate the grid for docking by keeping the box dimension of 15 Å around the binding pocket. Docking was performed using the extra precision (XP) module of Glide. Based on the binding scores, these various binding poses were sorted. We further used the molecular mechanics with generalised Born, and surface area solvation (MM-GBSA) based free binding energy calculations in the Prime module of Maestro (2020.3), Schrödinger to sort molecules based on binding energy. The Glide output pose viewer file (maegz format) of the protein-ligand docked complexes were run using the MM-GBSA module by keeping the default

settings. The docked structures were visualized in the Maestro. The molecular graphics were prepared using Pymol software suite [10].

S4 Method. Cloning, expression, and purification of AcrB

The cloning, expression, and purification of AcrB were performed using the protocol described earlier [11]. The AcrB coding gene was PCR amplified from gDNA of *E. coli*, cloned in pET22b vector, and transformed in *E. coli* Rosetta DE3 cells. Colonies obtained were then inoculated into 10 mL of primary media, which was further used to inoculate 1 L media for large-scale protein expression. Media was supplemented with 0.3 mM IPTG when OD_{600} of ~0.6 was reached. The culture was then grown further for 4 h at 37 ˚C at 200 rpm, and then cells were harvested by centrifugation at 9000 ×*g* for 15 min at 4 ˚C. The cell pellet was then dissolved in 20 mM HEPES pH 8.0, 150 mM NaCl (buffer 1), lysed by sonication, and then centrifuged at 18000 ×*g* for 1 h. The pellet obtained was then solubilized in buffer 1 supplemented with 0.2 %(w/v) DDM (buffer 2) for 16 h at 4 ˚C and centrifuged again at 18000 ×*g* for 1 h. The supernatant obtained was then mixed with Ni-NTA resins pre-equilibrated with buffer 2. After incubation with Ni-NTA for 1 h, the supernatant was removed, and Ni-NTA resins were washed with Buffer 1 supplemented with 0.02% DDM (Buffer 3). The AcrB was then eluted using buffer 3 supplemented with an increasing concentration of Imidazole (10 mM, 20 mM, 200 mM, and 500 mM). SDS-PAGE was performed to check the protein quality, and fractions containing AcrB were pooled together, concentrated to 500 µL, mixed with 2 mL of peptidisc solution 1 mg/mL, and concentrated further till 500 µL. After this, buffer exchange was performed by mixing 500 µL of AcrB in peptidisc [12] with 4.5 mL of buffer 1 and again concentrated till 500 µL; this process was repeated a total of 4 times. Then the sample was subjected to size exclusion chromatography using Superdex 200 increase 10/300 GL column. The purified protein was then concentrated and used for further experiments.

S5 Method. Site-directed mutagenesis, expression, and purification of AcrB

AcrB^{F178A}, AcrB^{F628A}, and AcrB$^{F615A, F617A, R620A}$ mutants were made after docking analysis and literature survey. Single primers were used to perform site-directed mutagenesis. AcrB^{F178A} was prepared using primer- 5’-GGGCGTGGGTGATGTTCAGTTGGCCGGTTCACAGTACGCGATGCG-3’, AcrB^{F628A} primer 5’-GGTCAGAATACCGGTATTGCGGCCGTTTCCTTGAAGGACTGGGCC -3’ was used to incorporate F628A mutation and 5’-GTGTTCGCCGTTAACGGCGCCGGCGCTGCGGGAGCTGGTCAGAATACCGGTATTGC -3’ primer was used to make AcrB$^{F615A, F617A, R620A}$ triple mutant. pET22b-*acrb* was used as a template to amplify these constructs, and the PCR product was further digested using the DpnI enzyme. The products were transformed into *E.coli* Top10 cells and sequenced to confirm the mutation. The positive clone was further transformed into *E.coli* C43 cell for protein purification. The colonies thus obtained were inoculated in a 10 mL primary LB media, which was used to inoculate a 1 liter secondary culture and incubated at 37ºC for four hours at 200 rpm to obtain an OD_{600} of 0.8. The cultures were induced using 0.3 mM IPTG and grown overnight at 16 ºC. Cells were harvested by centrifugation at 9000 ×*g* for 10 min at 4 ˚C. The cell pellet was then dissolved in 20 mM HEPES pH 8.0, 150 mM NaCl (buffer 1), lysed by sonication, and then centrifuged at 18000 ×*g* for 1 h. The pellet obtained was then solubilized in buffer 1 supplemented with 0.2 % (w/v) DDM (buffer 2) for 16 h at 4 ˚C and centrifuged again at 18000 ×*g* for 1 h. The supernatant obtained was then mixed with Ni-NTA resins pre-equilibrated with buffer 2. After incubation with Ni-NTA for 1 h, the supernatant was removed, and Ni-NTA resins were washed with Buffer 1 supplemented with 0.02% DDM (Buffer 3). The AcrB was then eluted using buffer 3 supplemented with an increasing concentration of Imidazole (10 mM, 20 mM, 200 mM, and 500 mM). SDS-PAGE was performed to check the protein quality, and fractions containing AcrB were pooled together for further use.

S6 Method. Persister assay for *P. aeruginosa*

The persister assay was performed by following a method described earlier [13], with some modifications.

Biphasic killing curves: The *P. aeruginosa* ATCC 27853 was grown to the stationary phase in LB for 48 h. After 48 h, the culture was diluted 1:20 in fresh LB, and then split into four separate tubes. The tubes were incubated in the presence or absence of ciprofloxacin (2 × MIC, 4 × MIC, and 8 × MIC) at 37 ºC for 8 h to determine the biphasic killing curves of the bacteria. The 100 µL volume of the sample from each tube was withdrawn at different time intervals (0 h, 4 h, and 8 h), appropriately diluted, and spotted onto LB agar plates to determine the viable bacteria. The concentration of ciprofloxacin at which a stable plateau phase is reached after the antibiotic killing was used in the subsequent assay.

Persister killing assay: For the persister killing assay, the stationary phase culture (grown for 48 h) of *P. aeruginosa* was diluted 1:20 in fresh LB. The culture was divided into two separate tubes, and one tube was incubated in the presence of ciprofloxacin (8 × MIC) for 3 h at 37 ºC. The untreated culture tube was included as a control. The samples were consecutively withdrawn at 1-hour intervals for 3 hours, diluted appropriately, and spotted onto LB agar plates to determine viable counts. After 3 h, the bacterial suspension was centrifuged at 4000 ×*g* for 5 min, washed twice in 1X PBS, resuspended in fresh media, and again incubated for 20 h at 37 ºC to evaluate that persistence is transient and reversible. The killing and re-growth procedure was repeated for 3 consecutive days. The MIC of ciprofloxacin was determined every day to confirm that the tolerance in bacterial cells is due to persistence and not resistance. On the fourth day, the culture tube having ciprofloxacin treatment for 3 days was incubated in the presence of ciprofloxacin (8 × MIC) or ciprofloxacin (8 × MIC) combined with Ar5 (16 µg/mL) for 3 h at 37 ºC. The samples were withdrawn at 1 h intervals for 3 h, appropriately diluted, spotted onto LB agar plates, and incubated for 48 h to determine the viable persister cells.

S7 Method. Biofilm eradication assay

P. aeruginosa ATCC BAA-2795 was grown overnight in LB media at 37 ºC. Then, the culture was diluted at 1:200 in fresh M63 medium (12 g/L KH_2PO_4, 28 g/L K_2HPO_4, 8 g/L NH_4SO_4, 1 mM $MgSO_4$, 0.5% Casamino acids, 0.2% Citrate) [14]. The bacterial culture was loaded into a 96-well flat-bottom plate and incubated for 48 h at 37 ºC under static conditions to allow biofilm formation. After incubation, the wells were washed with 1X PBS to remove non-adherent bacteria and treated with compounds alone (Ar1, Ar5, Ar11, Ar18) at sub-inhibitory concentrations (1/4 × MICs), levofloxacin (1 × MIC) alone or a combination of levofloxacin and compounds. The plates were incubated for 24 h, washed with 1X PBS, and fixed with 99% methanol for 15 min. The plate was left to dry for a few minutes in laminar airflow, and the adherent bacteria were stained with filtered crystal violet (CV; 0.5% w/v) for 5 minutes at room temperature. The wells were washed with water to remove the surplus stain until negative control (without biofilm) wells appeared colorless. Further, the bacterial-bound stain was solubilized with 33% acetic acid, and the absorbance of the solubilized stain was measured at 570 nm. The results represent the percentage of biofilm formation compared to the untreated control.

Further, the quantification of bacterial viability within biofilm was assessed. The plates were incubated similarly to the CV assay for biofilm formation, washed and treated with compounds (Ar1, Ar5, Ar11, Ar18) alone, combined with levofloxacin for 24 h, and then washed with 1X PBS to remove the planktonic cells. The biofilm was stained with MTT (1 mg/mL), and plates were incubated for 4 h at 37 ºC. After incubation, wells were washed once with water to remove the surplus dye and solubilized using a solubilization solution (40% (v/v) dimethylformamide in 2% (v/v) glacial acetic acid added with 16% (w/v) sodium dodecyl sulfate) [15]. The absorbance (OD_{570nm}) of the released stain was measured, and the results represent the percentage of live adherent bacteria within biofilm compared to untreated control.

S8 Method. Outer membrane permeabilization assay

The assay was performed by measuring the fluorescence of 8-anilino-1-napthylenesulfonic acid (ANS, Sigma-Aldrich) incorporation into the outer membrane of *E. coli*, as described previously with some modifications [16]. The overnight grown bacterial culture of *E. coli* $AG100_{tet}$ was diluted 1:20 in CA-MHB and grown to log phase at 37 ˚C, 200 rpm. The bacterial culture was centrifuged at 2300 ×*g* for 5 min, washed twice, and resuspended to OD_{600nm} 0.5 in 5 mM HEPES buffer (pH 7.2). The bacterial suspension was added to 96-well white, clear-bottom plates containing compounds (Ar1-Ar24) at 256 µg/mL, 128 µg/mL, and 64 µg/mL. PAβN [16], colistin, and polymyxin B at three different concentrations (256 µg/mL, 128 µg/mL, and 64 µg/mL) were included as a control for comparison. 2 µL of 3 mM ANS was added to each well and fluorescence was measured in a microplate reader at the excitation and emission wavelength of 375 nm and 510 nm, respectively.

S9 Method. Membrane depolarization assay

The membrane depolarization induced by compounds was determined by measuring the fluorescence of 3, 3′-Dipropylthiadicarbocyanine iodide ($DiSC_3(5)$), as described previously [17, 18]. The bacterial culture of *E. coli* $AG100_{tet}$ was grown to log phase and washed twice in 5 mM HEPES buffer (containing 5 mM glucose, 100 mM KCl, pH 7.2). The OD_{600nm} of bacterial suspension was adjusted to 0.5 and further diluted 10 times to obtain the final culture density (10^7 CFU/mL). Next, the bacterial suspension was incubated with 0.4 µM $DISC_3(5)$ for 60 min at 37 ºC. Afterward, the $DISC_3(5)$ treated culture was dispensed into a 96-well black flat-bottom plate containing compounds (Ar1-Ar24) at 1 × MIC (64 µg/mL) and ½ × MIC (32 µg/mL). Polymyxin B (8 µg/mL and 4 µg/mL) was included as a positive control. The leakage in fluorescence was measured for 30 min in a microplate reader at an excitation and emission wavelength of 622 nm and 670 nm, respectively.

S10 Method. ATP determination assay

According to the manufacturer's instructions, the bacterial intracellular ATP levels were measured using an ATP determination kit (Invitrogen, Life Technologies, U.S.). As described earlier [19], the bacterial culture *E. coli* AG100$_{tet}$ was grown to OD$_{600nm}$ of 0.3 and treated with sub-inhibitory concentrations (1/4 × MIC) of compounds (Ar1-Ar24) and incubated for 4 h at 37 ºC. After 4 h, the bacterial cells were lysed in an ultrasonication water bath, followed by alternative heat and cold shock. Further, 10 µL of the lysate was transferred to a 96-well black flat-bottom plate and then dispensed with a 90 µL standard reaction solution. The persistent ATP from cell lysate was measured and plotted as relative luminescence units. The culture treated with CCCP (16 µg/mL) was included as a positive control.

S11 Method. Membrane fluidity assay

The effect of compounds on membrane fluidity was determined by a method described earlier [20], with some modifications. The overnight culture of *E. coli* AG100$_{tet}$ was diluted 1:100 in fresh LB and grown to OD$_{600nm}$ 0.5 at 37 ˚C, 200 rpm. Further, bacterial culture was centrifuged using pre-warmed microtubes (30 ˚C) in a pre-warmed centrifuge at 16,000 ×*g* for 30 s at 30 ˚C. The harvested cells were washed four times in pre-warmed (30 ˚C) laurdan buffer (137 mM NaCl, 2.7 mM KCl, 10 mM Na_2HPO_4, 1.8 mM KH_2PO_4, 0.2% glucose, 1% DMF). The cells were resuspended by vortexing shortly to a final OD$_{600nm}$ 0.4 in laurdan buffer. 100 µL of the cell suspension was added to a 96-well black plate containing compounds at 1 × MIC, and the fluorescence was measured (for baseline fluorescence) for excitation wavelength 350 nm at emission wavelengths 440 nm and 490 nm for 10 min (2 min interval) in a microplate reader pre-warmed at 30 ˚C. Further, 100 µL of cell suspension containing 10 µM laurdan was added to the same plate, and fluorescence was measured for 20 min (2 min time interval). PAβN (1 × MIC), valinomycin (50 µM) [21], and benzyl alcohol (50 mM; a known membrane fluidizer) were included for comparison. The baseline fluorescence for each well was subtracted, and

laurdan generalized polarization (GP) was calculated according to the formula: $(I_{440} - I_{490}) / (I_{440} + I_{490})$, where I_{440}, I_{490} refer to fluorescence intensities at 440 nm and 490 nm respectively.

S12 Method. Growth kinetics determination

The OD_{600nm} of freshly grown *P. aeruginosa* PAO1 was adjusted to 0.1 in CA-MHB. The sub-inhibitory (1/4 × MIC) concentrations of Ar1 (16 µg/mL), Ar5 (16 µg/mL), Ar11 (16 µg/mL), and Ar18 (16 µg/mL) were mixed with the bacterial culture into 96-well microplate. The untreated bacterial culture was included as a control. Growth curves were established under the wavelength of OD_{600nm} with an interval of 1 h at 37 °C by a microplate reader (BioTek, U.S.).

S13 Method. Motility assays

The effect of compounds on *P. aeruginosa* motility was assessed by a method described earlier [22]. The overnight grown culture of *P. aeruginosa* PAO1 and PAO750 in LB broth were spotted on to swimming (0.1% w/v tryptone, 0.05% w/v yeast extract, 0.5% w/v NaCl, 0.3% w/v bacteriological agar) and swarming plates (0.8% w/v nutrient broth, 0.5% w/v glucose, 0.5% w/v bacteriological agar). The twitching motility was assessed according to a protocol described earlier [23]; briefly, *P. aeruginosa* PAO1 and PAO750 was grown to the mid-log phase in LB media. The 1.5 µL culture was then stab inoculated through a thin overnight-dried LB agar (1%) layer to the bottom of the petri plates (10 mL media per plate). For treatment conditions, the plates were supplemented with Ar1, Ar5, Ar11, and Ar18 at sub-inhibitory concentrations (16 µg/mL) and incubated for 24 h (swimming motility), 48 h (swarming motility), and 36 h (twitching motility) at 37 ºC. The control plates were supplemented with an equal volume of DMSO as in treatment plates. The ability of bacteria to strongly adhere and twitch on the polystyrene surface was then examined by carefully removing the agar after incubation, and staining the attached cells with crystal violet (1% w/v) solution for 5 min, and washing to remove the excess stain.

S14 Method. RNA isolation, RNA sequencing, and qRT-PCR

The overnight culture of *P. aeruginosa* PAO1 propagated in LB was diluted 1:1000 in fresh media and incubated in the presence of Ar5 (16 µg/mL) and PAβN (16 µg/mL) for 17 h at 37 ºC. After incubation, the 5 mL culture was centrifuged, and the pellet was stored overnight at -80 ºC. The pellet was removed from the freezer, mixed with 1 mL TRIzol™ reagent (Invitrogen), and immediately placed on ice. The solution was subject to bead beating for 45 sec, immediately placed on ice, and centrifuged at 13,000 ×*g* for 10 min at 4 ºC. The supernatant was transferred to another tube and gently mixed with an equal volume of absolute ethanol (molecular biology grade). Further, the total RNA was extracted using the RNeasy Mini Kit (QIAGEN).

RNA Sample QC: All Direct RNA samples were quantified using Qubit RNA BR Assay (Invitrogen, Cat# Q10211). RNA purity was checked using QIAxpert, and RNA integrity was assessed on TapeStation using RNA screenTapes (Agilent, Cat# 5067-5576). The QC passed samples were taken for further processing.

RNA Library Prep Protocol: NEB Ultra II directional RNA-Seq Library Prep kit (NEB, Cat# E7760L) protocol was used to prepare libraries for total RNA sequencing. An initial Concentration of 100 ng of total RNA was taken for the assay. First, the total RNA was hybridized with QIAseq® FastSelect™ –5S/16S/23S reagent mixture (Qiagen, Cat# 335927) by heat-fragmenting and cooling the reaction from 75°C to 25°C, followed by Bead clean-up. The cleaned-up ribo-depleted RNA fragments were copied into first-strand cDNA using reverse transcriptase. Second strand cDNA synthesis was performed using DNA Polymerase I and RNase H enzyme. The cDNA fragments were then subjected to a series of enzymatic steps that repair the ends, tails the 3' end with a single 'A' base, followed by ligation of the adapters. The adapter-ligated products were then purified and enriched using the following thermal conditions: initial denaturation of 98°C for 30 sec; 13 cycles of - 98°C for 10 sec, 65°C for 75 sec; final extension of 65°C for 5 mins. The amplified libraries were purified

and checked for fragment size distribution on Fragment Analyzer using HS NGS Fragment Kit (1-6000bp) (Agilent, Cat# DNF-474-1000).

Sequencing Protocol: Prepared libraries were quantified using Qubit High Sensitivity Assay (Invitrogen, Cat# Q32851). The obtained libraries were pooled and diluted to the final optimal loading concentration before cluster amplification on the Illumina Novaseq 6000 instrument to generate 40M, 150 bp paired-end reads.

Contamination removal: For the RNA-Seq analysis, we begin by removing the unwanted sequences, especially non-polyA-tailed RNAs, from the sample (assuming that poly-A tailed RNAs are sequenced). The unwanted sequences include - ribosomal RNAs, transfer RNAs, adapter sequences, and others. Contamination removal was performed using Bowtie2 (2.2.4).

Read alignment: The paired-end reads are aligned to the reference *Pseudomonas aeruginosa* genome (https://ftp.ncbi.nlm.nih.gov/genomes/all/GCF/000/006/765/GCF_000006765.1_A M676v1/). Alignment was performed using HISAT2 (2.1.0).

Expression estimation: The aligned reads are used to estimate the gene expression. The raw read counts were estimated using FeatureCount (1.5.2). Read count data were normalized using DESeq2.

Differential expression analysis: The raw read counts were normalized using DESeq2.The ratio of normalized read counts for treated over control was taken as the fold change. Genes were first filtered based on the p-value (<= 0.05). The distribution of these log2 (foldchange) values was found to be normally distributed. Those genes which were found to have -1 <= log2(foldchange) >= 1 were considered as statistically significant.

qRT-PCR: The transcript levels of the *P. aeruginosa* PAO1 quorum sensing genes were determined by quantitative real-time polymerase chain reaction (qRT-PCR). Following a method described earlier, we isolated RNA from *P. aeruginosa* after treatment and quantified the expression of lasI, lasR (lasI-lasR QS system), rhlI,

rhlR (rhlI-rhlR QS system), pqsA, pqsB, pqsC, pqsD, pqsE, pqsH, pqsR (PQS QS system), and qscR (regulator of las QS). Gene transcript levels were normalized against the *P. aeruginosa* 16s rRNA gene. The real-time quantification of RNA templates was performed using SuperScript III Platinum SYBR Green One-step qRT-PCR kit (InvitrogenTM, US) in StepOne TM Real-Time PCR System (Applied Biosystems, US). The relative fold expression levels were calculated using the $2^{-\Delta\Delta CT}$ method.

S15 Method. Determination of bile salts potentiation

P. aeruginosa PAO1 was grown overnight and subcultured 1/20 in CA-MHB containing PAβN (0, 25, 50 µg/mL) or Ar5 (16 µg/mL). The cultures were further grown to OD_{600nm} 0.6 at 37 ˚C, serially diluted in CA-MHB, and spread 50 µL on MHA plates containing appropriate concentrations of Ar5 and PAβN with or without 0.15% bile salts. Further, plates were incubated for 24 h at 37 ˚C, colonies were counted, and CFU/mL was determined.

S16 Method. *In silico* absorption, distribution, metabolism, and excretion (ADME) analysis

In silico ADME study was performed for compounds using physiological descriptors for pharmacokinetic properties such as lipophilicity, aqueous solubility, human gastrointestinal absorption, blood-brain barrier penetration, cytochrome P450 inhibition, and drug-likeness. The well-known EPIs PAβN and reserpine were included for comparison. The study was performed using SwissADME (http://www.swissadme.ch/) web platform [24].

Supporting information references

1. Lerchen HG, Wittrock S, Stelte-Ludwig B, Sommer A, Berndt S, Griebenow N, et al. Antibody–Drug Conjugates with Pyrrole-Based KSP Inhibitors as the Payload Class. Angewandte Chemie International Edition. 2018;57(46):15243-7.
2. Han X, Jiang M, Mayweg A, Wang L, Yang S. Novel pyrrole derivatives for the treatment of cancer. WO2014154723. 2014.
3. Kumar V, Awasthi A, Metya A, Khan T. A metal-free domino process for regioselective synthesis of 1, 2, 4-trisubstituted pyrroles: application toward the formal synthesis of ningalin B. The Journal of Organic Chemistry. 2019;84(18):11581-95.
4. Clinical, Institute LS. Performance standards for antimicrobial susceptibility testing. Clinical and Laboratory Standards Institute Wayne, PA; 2017.
5. Chen I-J, Foloppe N. Drug-like bioactive structures and conformational coverage with the LigPrep/ConfGen suite: comparison to programs MOE and catalyst. Journal of chemical information and modeling. 2010;50(5):822-39.
6. Nakashima R, Sakurai K, Yamasaki S, Hayashi K, Nagata C, Hoshino K, et al. Structural basis for the inhibition of bacterial multidrug exporters. Nature. 2013;500(7460):102-6.
7. Madhavi Sastry G, Adzhigirey M, Day T, Annabhimoju R, Sherman W. Protein and ligand preparation: parameters, protocols, and influence on virtual screening enrichments. Journal of computer-aided molecular design. 2013;27(3):221-34.
8. Shivakumar D, Harder E, Damm W, Friesner RA, Sherman W. Improving the prediction of absolute solvation free energies using the next generation OPLS force field. Journal of chemical theory and computation. 2012;8(8):2553-8.
9. Halgren T. New method for fast and accurate binding-site identification and analysis. Chemical biology & drug design. 2007;69(2):146-8.
10. DeLano WL. Pymol: An open-source molecular graphics tool. CCP4 Newsl Protein Crystallogr. 2002;40(1):82-92.
11. Tambat R, Mahey N, Chandal N, Verma DK, Jangra M, Thakur KG, et al. A Microbe-Derived Efflux Pump Inhibitor of the Resistance-Nodulation-Cell Division Protein Restores Antibiotic Susceptibility in Escherichia coli and Pseudomonas aeruginosa. ACS infectious diseases. 2022.
12. Angiulli G, Dhupar HS, Suzuki H, Wason IS, Van Hoa FD, Walz T. New approach for membrane protein reconstitution into peptidiscs and basis for their adaptability to different proteins. Elife. 2020;9.
13. Pu Y, Zhao Z, Li Y, Zou J, Ma Q, Zhao Y, et al. Enhanced efflux activity facilitates drug tolerance in dormant bacterial cells. Molecular cell. 2016;62(2):284-94.
14. Kim S, Li X-H, Hwang H-J, Lee J-H. Thermoregulation of Pseudomonas aeruginosa biofilm formation. Applied and Environmental Microbiology. 2020;86(22):e01584-20.
15. Riss TL, Moravec RA, Niles AL, Duellman S, Benink HA, Worzella TJ, et al. Cell viability assays. Assay Guidance Manual [Internet]. 2016.
16. Lamers RP, Cavallari JF, Burrows LL. The efflux inhibitor phenylalanine-arginine beta-naphthylamide (PAβN) permeabilizes the outer membrane of gram-negative bacteria. PloS one. 2013;8(3):e60666.
17. Cheng M, Huang JX, Ramu S, Butler MS, Cooper MA. Ramoplanin at bactericidal concentrations induces bacterial membrane depolarization in Staphylococcus aureus. Antimicrobial agents and chemotherapy. 2014;58(11):6819-27.
18. Lee D, Powers J, Pflegerl K, Vasil M, Hancock R, Hodges R. Effects of single d-amino acid substitutions on disruption of β-sheet structure and hydrophobicity in cyclic 14-residue antimicrobial peptide analogs related to gramicidin S. The Journal of peptide research. 2004;63(2):69-84.

19. Machado D, Fernandes L, Costa SS, Cannalire R, Manfroni G, Tabarrini O, et al. Mode of action of the 2-phenylquinoline efflux inhibitor PQQ4R against Escherichia coli. PeerJ. 2017;5:e3168.
20. Wenzel M, Vischer NO, Strahl H, Hamoen LW. Assessing membrane fluidity and visualizing fluid membrane domains in bacteria using fluorescent membrane dyes. Bio-protocol. 2018;8(20):e3063-e.
21. El Khoury M, Swain J, Sautrey G, Zimmermann L, Van Der Smissen P, Décout J-L, et al. Targeting bacterial cardiolipin enriched microdomains: an antimicrobial strategy used by amphiphilic aminoglycoside antibiotics. Scientific reports. 2017;7(1):10697.
22. Imperi F, Massai F, Ramachandran Pillai C, Longo F, Zennaro E, Rampioni G, et al. New life for an old drug: the anthelmintic drug niclosamide inhibits Pseudomonas aeruginosa quorum sensing. Antimicrobial agents and chemotherapy. 2013;57(2):996-1005.
23. Coleman SR, Bains M, Smith ML, Spicer V, Lao Y, Taylor PK, et al. The small RNAs PA2952. 1 and PrrH as regulators of virulence, motility, and iron metabolism in Pseudomonas aeruginosa. Applied and Environmental Microbiology. 2021;87(3):e02182-20.
24. Daina A, Michielin O, Zoete V. SwissADME: a free web tool to evaluate pharmacokinetics, drug-likeness and medicinal chemistry friendliness of small molecules. Scientific reports. 2017;7(1):1-13.
